# Global burden of gynaecological cancers in 2022 and projections to 2050

**DOI:** 10.7189/jogh.14.04155

**Published:** 2024-08-16

**Authors:** Binhua Zhu, Hao Gu, Zhihan Mao, Narasimha M Beeraka, Xiang Zhao, Mahesh Padukudru Anand, Yufei Zheng, Ruiwen Zhao, Siting Li, Prasath Manogaran, Ruitai Fan, Vladimir N Nikolenko, Haixiao Wen, Basappa Basappa, Junqi Liu

**Affiliations:** 1 Department of Radiation Oncology, The First Affiliated Hospital of Zhengzhou University, Zhengzhou, China; 2 Cancer Center, The First Affiliated Hospital of Zhengzhou University, Zhengzhou, China; 3 Henan Medical College, Zhengzhou University, Zhengzhou, China; 4 Raghavendra Institute of Pharmaceutical Education and Research (RIPER), Anantapuramu, Chiyyedu, Andhra Pradesh, India; 5 Department of Pulmonary Medicine, JSS Medical College, JSS Academy of Higher Education & Research (JSS AHER), Mysuru, Karnataka, India; 6 Herman B. Wells Center for Pediatric Research, Department of Pediatrics, Indiana University School of Medicine, Indianapolis, Indiana, USA; 7 Department of Human Anatomy and Histology, I.M. Sechenov First Moscow State Medical University (Sechenov University), Moscow, Russian Federation; 8 Department of Biotechnology, Bharathiar University, Coimbatore, Tamil Nadu, India; 9 Department of Clinical and Translational Sciences, Joan C. Edwards School of Medicine, Marshall University, Huntington, West Virginia, USA; 10 Department of Gynecologic Oncology, The First Affiliated Hospital of Zhengzhou University, Zhengzhou, China; 11 Laboratory of Chemical Biology, Department of Studies in Organic Chemistry, University of Mysore, Mysore, Karnataka, India

## Abstract

**Background:**

The incidence and mortality of gynaecological cancers can significantly impact women's quality of life and increase the health care burden for organisations globally. The objective of this study was to evaluate global inequalities in the incidence and mortality of gynaecological cancers in 2022, based on The Global Cancer Observatory (GLOBOCAN) 2022 estimates. The future burden of gynaecological cancers (GCs) in 2050 was also projected.

**Methods:**

Data regarding to the total cases and deaths related to gynaecological cancer, as well as cases and deaths pertaining to different subtypes of GCs, gathered from the GLOBOCAN database for the year 2022. Predictions for the number of cases and deaths in the year 2050 were derived from global demographic projections, categorised by world region and Human Development Index (HDI).

**Results:**

In 2022, there were 1 473 427 new cases of GCs and 680 372 deaths. The incidence of gynecological cancer reached 30.3 per 100 000, and the mortality rate hit 13.2 per 100 000. The age-standardised incidence of GCs in Eastern Africa is higher than 50 per 100 000, whereas the age-standardised incidence in Northern Africa is 17.1 per 100 000. The highest mortality rates were found in East Africa (ASMR (age-standardised mortality rates) of 35.3 per 100 000) and the lowest in Australia and New Zealand (ASMR of 8.1 per 100 000). These are related to the endemic areas of HIV and HPV. Very High HDI countries had the highest incidence of GCs, with ASIR (age-standardised incidence rates) of 34.8 per 100 000, and low HDI countries had the second highest incidence rate, with an ASIR of 33.0 per 100 000. Eswatini had the highest incidence and mortality (105.4 per 100 000; 71.1 per 100 000) and Yemen the lowest (5.8 per 100 000; 4.4 per 100 000). If the current trends in morbidity and mortality are maintained, number of new cases and deaths from female reproductive tract tumours is projected to increase over the next two decades.

**Conclusions:**

In 2022, gynaecological cancers accounted for 1 473 427 new cases and 680 372 deaths globally, with significant regional disparities in incidence and mortality rates. The highest rates were observed in Eastern Africa and countries with very high and low HDI, with Eswatini recording the most severe statistics. If current trends continue, the number of new cases and deaths from gynaecological cancers is expected to rise over the next two decades, highlighting the urgent need for effective interventions.

The incidence of gynaecological cancers (GCs) in the female reproductive system have been increasing due to improper lifestyle patterns, dietary habits, and genetic factors [1]. Gynaecological cancers (GCs) include vulvar cancer (ICD-10 C51), vaginal cancer (ICD-10 C52), cervical cancer (ICD-10 C53), uterine cancer (ICD-10 C54), ovarian cancer (ICD-10 C56), and fallopian tube cancer (ICD-10 C57.0) depending on the location of the tumour. Among these cancers, the incidence of fallopian tube tumours is very rare [2,3]. Endometrial cancer, ovarian cancer, and cervical cancer represent the highly occurring cancers and account for more than one-third of the newly diagnosed cancers globally in females [4,5].

Gynaecological cancers, encompassing ovarian, cervical, endometrial (uterine), vulvar, and vaginal cancers, present a multifaceted landscape of risk factors, spanning genetic predispositions, environmental exposures, and behavioural patterns. Analysing each cancer type reveals a nuanced interplay of these factors [6]. Ovarian cancer, for instance, shows potential links to environmental exposures like asbestos and talcum powder, alongside behavioural influences such as dietary habits and reproductive history. Conversely, cervical cancer's primary environmental risk factor is persistent infection with high-risk HPV types, compounded by behaviours like sexual activity and smoking, and influenced by dietary choices [6]. Endometrial cancer illustrates the significance of hormonal factors, with oestrogen exposure, obesity, and diet playing pivotal roles. Vulvar and vaginal cancers, while sharing HPV infection as a common environmental factor, also exhibit the impact of behaviours like smoking and sexual activity [7,8]. Despite variations among GCs, certain behavioural factors consistently emerge as influential across the spectrum. Lifestyle modifications, including healthy diet choices, avoidance of tobacco, safe sexual practices, and HPV vaccination, offer significant avenues for risk reduction. Regular medical screenings further augment prevention efforts by enabling early detection and intervention [6]. Comprehensive analysis underscores the complex interaction of genetic, environmental, and behavioural factors in shaping the risk landscape of GCs. By addressing modifiable risk factors through proactive lifestyle measures and medical interventions, individuals can substantially mitigate their susceptibility to these diseases, thereby enhancing overall well-being and longevity [6].

Incidence and mortality of GCs could affect the quality of life of women and cause a higher health care burden for health care organisations around the world [9,10]. GLOBOCAN reports provide comprehensive estimates of cancer incidence and mortality for 185 countries or geographical regions worldwide. These findings enable to describe the current global cancer burden, offering valuable insights for policymakers and researchers. Previously, the global burden of individual GCs is still no report on the world burden of corpus uteri and vaginal cancer in 2022, and there is no relevant analysis on the overall incidence and mortality of GCs. In this article, we presented the global burden of each common GC and its prediction in detail and summarised the overall incidence and mortality of GCs to enable a comprehensive understanding of cancers pertinent to the female reproductive system. In addition, the 2050 prediction rates for various types of GCs have not been reported previously. In this study, we aimed to assess the global epidemiological status of GCs in 2022 depending on factors such as geographical and socioeconomic developments, and age and provided different GCs that are commonly occurring among different age groups. In this study, specifically vulvar cancer, vaginal cancer, cervical cancer, uterine cancer, and ovarian cancer are discussed. This report demonstrates the need for early screening strategies for GCs in different ages. A global update on the current incidence and mortality pertinent to GCs by using the latest cancer estimates was provided. The burden of morbidity and mortality related to these cancers by 2050 was predicted to provide evidence for local governments to formulate prevention and management policies.

## METHODS

### Data sources and methods

Data pertinent to this study was retrieved from GLOBOCAN 2022 estimates, encompassing data on incidence, mortality, and prevalence for the year 2022. The study covered 185 countries or territories and included 36 distinct cancer types, classified based on sex and age group [11]. GLOBOCAN 2022 examines cancer incidence and mortality rates with a minimum unit of five years of age, hence this article also discusses cancer-related data in stages of five years. Data on new cases and deaths of GCs were obtained from the GLOBOCAN 2022 database for the individuals of age groups (0–4, 5–9, 80–84, 85 years, and above 85 years) from 185 countries and world regions [11]. The incidence and mortality rate of each category of gynaecological cancer including vulvar cancer, vaginal cancer, cervical cancer, uterine corpus cancer, and ovarian cancer in 2022 were also obtained from the database. The population data related to these cancers reported in 2022 were extracted from the United Nations (UN) website. Health information for some countries was obtained from the WHO website [12]. Data related to five GCs was ascertained to obtain the incidence and mortality of GCs and described the proportion of each GC around the world in 2022. The number of new cases, deaths, incidence and mortality of all kinds of GCs and each category of GC for different world regions and countries with HDI levels was estimated and subsequently predicted for 2050 for various possibilities including if the trends remained the same or if it increased or decreased over the next two decades. Annualised rate of change was projected for seven scenarios (standardised rate of change ranged from –3% to +3% per year). Age-standardised (incidence or mortality) rate per 100 000 person-years based on the 1966 Segi whereas World standard population (18 age groups) was taken into consideration for this study [13,14].

The findings were represented on a country-by-country basis and consolidated based on the 20 world regions defined by the United Nations [15] and subsequently described and ascertained the results depending on the UN’s four-tier HDI in 2022 [16]. The predictions regarding future incidence and mortality rates for cases and deaths attributed to GCs worldwide in the year 2050 rely on demographic projections assuming the continuity of current trends from the baseline year, 2022. Predictions were ascertained by applying the observed incidence or mortality rates from the base year, 2022, to the projected population data provided by the United Nations Development Program (UNDP).

## RESULTS

### Incidence and mortality: Worldwide

**Figure 1:**
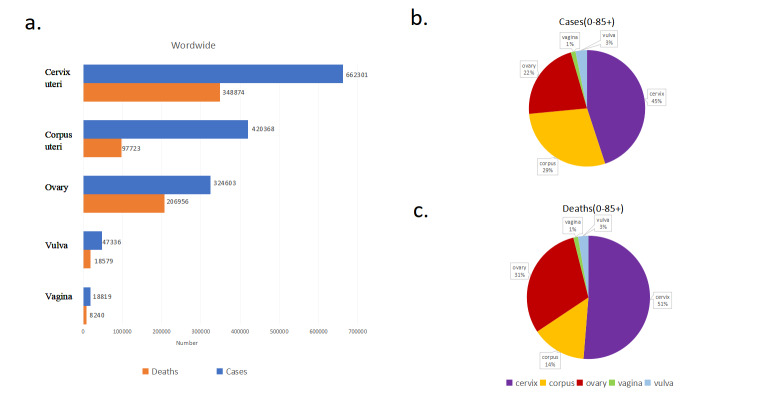
Incidence and mortality of various gynaecological cancers (GCs) and Their Proportions in 2022. **Panel A.** The incidence and mortality rates of different types of GCs in 2022, illustrating the overall burden of each cancer type on the population. **Panel B** and **Panel C.** The proportion of each type of gynaecological cancer cases, deaths relative to the total number of GC cases in age groups 0 to over 85 years. These charts provide a visual breakdown of the distribution of various GCs, highlighting the most prevalent forms and their impact on public health.

In 2022, the number of new cancer cases that occurred due to GCs accounted for 15.25% of all the cases of women with cancer and the number of new cancer deaths that occurred due to GCs accounted for 15.77% of all the deaths of women with cancer. A total of 1 473 427 women were diagnosed with gynaecological cancer in 2022, overall, 44.95% of them reported cervical cancer. A total of 680 372 women died of gynaecological cancer, and among these women, specifically, 51.28% died of cervical cancer. The burden of various gynaecological cancer cases is described in **Figure 1**, panel A. The incidence of GCs was 30.3 per 100 000 individuals and the mortality rate was 13.2 per 100 000 individuals (**Figure 1**, panels B–C). The incidence of corpus uterine cancer attained second place among GCs, but the mortality rate was minimal when compared to ovarian cancer. Vulvar and vaginal cancers were rare GCs. Both incidence and mortality were lower than one per 100 000. During 2022, gynaecologic genital tract cancers accounted for three of the top ten cancers in women. The incidence of cervical cancer, corpus uteri cancer, and ovarian cancer in women ranked fourth, sixth, and seventh among all the cancers respectively. Detailed data are described in **Table 1**.

**Table 1:** The incidence and mortality rates for patients diagnosed with cervical cancer, corpus uteri cancer, vaginal cancer, vulvar cancer, and other types of cancer in women were detailed respectively

	All				Cervix uteri				Corpus uteri				Ovary				Vagina				Vulva			
**Population**	**Incidence**		**Mortality**		**Incidence**		**Mortality**		**Incidence**		**Mortality**		**Incidence**		**Mortality**		**Incidence**		**Mortality**		**Incidence**		**Mortality**	
	**No.**	**ASR**	**No.**	**ASR**	**No.**	**ASR**	**No.**	**ASR**	**No.**	**ASR**	**No.**	**ASR**	**No.**	**ASR**	**No.**	**ASR**	**No.**	**ASR**	**No.**	**ASR**	**No.**	**ASR**	**No.**	**ASR**
Northern America	122966	38.5	38136	9.6	15654	6.4	6692	2.2	73977	22.3	13543	3.2	24484	7.5	15554	3.8	1664	0.43	471	0.10	7187	1.90	1876	0.37
Eastern Asia	352498	27.5	123834	8.3	167528	13.4	62094	4.3	100275	7.5	17818	1.1	75773	6.0	40264	2.7	3198	0.22	1287	0.08	5724	0.35	2371	0.13
Eastern Africa	72566	50.7	47940	35.3	58145	40.4	39476	28.9	3809	3.0	1272	1.1	7690	5.3	5518	4.2	787	0.55	467	0.35	2135	1.30	1207	0.83
Middle Africa	20718	39.4	13975	28.1	16268	31.1	11293	22.9	1164	2.4	381	0.9	2458	4.3	1794	3.5	277	0.50	166	0.31	551	1.00	341	0.65
Northern Africa	20194	17.1	10565	9.0	7686	6.5	4425	3.8	4302	3.7	962	0.8	7145	6.0	4687	4.0	284	0.24	125	0.10	777	0.65	366	0.30
Southern Africa	17016	48.5	9775	28.2	12351	34.9	7114	20.4	1957	5.9	828	2.5	1677	4.9	1424	4.2	255	0.73	84	0.25	776	2.10	325	0.89
Western Africa	43552	36.8	25158	22.1	31249	26.7	18306	16.3	3672	3.4	1219	1.2	6790	5.1	4601	3.8	451	0.35	277	0.22	1390	1.20	755	0.63
Caribbean	9699	32.1	4757	14.6	4012	14.0	2397	7.7	3855	12.0	1210	3.4	1450	4.9	1012	3.2	190	0.62	79	0.25	192	0.55	59	0.13
Central America	28952	27.8	13717	13.0	15119	14.3	7646	7.2	6725	6.7	1689	1.6	6175	6.0	4033	3.9	328	0.31	107	0.09	605	0.53	242	0.20
South-Eastern Asia	131807	32.9	68407	16.8	69886	17.4	38703	9.5	26601	6.6	7936	1.9	32113	8.1	20514	5.1	968	0.22	441	0.10	2239	0.54	813	0.19
South Central Asia	252898	24.9	153768	15.3	153944	15.1	95962	9.5	27172	2.7	10143	1.0	61931	6.1	42839	4.3	5765	0.58	2827	0.28	4086	0.40	1997	0.20
Western Asia	28420	20.9	12470	9.0	5724	4.1	3036	2.2	13382	10.1	3079	2.2	8406	6.1	5930	4.3	240	0.18	121	0.09	668	0.46	304	0.20
Eastern Europe	127647	47.5	52175	16.5	35052	15.7	16669	6.3	57095	19.2	13388	3.5	29416	11.0	19165	6.1	1111	0.34	510	0.13	4973	1.30	2443	0.55
Northern Europe	34971	34.3	14037	10.2	5659	8.2	2145	2.2	16736	14.8	4218	2.6	9787	9.1	6586	4.8	388	0.34	188	0.12	2401	1.90	900	0.47
Southern Europe	48925	30.4	19686	9.1	7792	6.4	3740	2.2	23786	14.0	5824	2.3	13265	8.4	8398	4.1	646	0.28	278	0.10	3436	1.30	1446	0.43
Western Europe	61837	27.8	25631	8.9	9716	6.6	4396	2.1	27257	11.2	6842	2.0	17004	7.1	12083	4.1	1019	0.38	365	0.10	6841	2.40	1945	0.50
Australia-New Zealand	7714	29.3	2770	8.1	1047	5.2	386	1.4	3884	14.2	833	2.3	2177	8.0	1384	4.0	126	0.43	41	0.11	480	1.50	126	0.28
Melanesia	2204	46.6	1280	28.6	1340	27.6	873	19.3	466	10.7	121	2.9	359	7.5	262	5.8	22	0.51	19	0.44	17	0.35	5	0.12
South America	88596	30.5	42171	13.7	44040	15.6	23471	7.8	24151	8.2	6389	2.0	16447	5.6	10866	3.5	1100	0.35	387	0.11	2858	0.85	1058	0.28
Micronesia	120	40.4	60	19.8	54	18.6	33	10.9	44	14.4	8	2.5	22	7.3	19	6.4	0	0.00	0	0.00	0	0.00	0	0.00
Polynesia	127	34.1	60	16.1	35	9.6	17	4.6	58	15.5	20	5.4	34	9.0	23	6.0	0	0.00	0	0.00	0	0.00	0	0.00
Very HDI country	499048	34.8	189998	10.7	107148	9.3	48363	3.3	237561	15.5	52465	2.6	120904	8.2	77625	4.3	5877	0.33	2238	0.10	27558	1.40	9307	0.37
High HDI country	532724	27.8	221848	10.8	265503	14.1	118418	5.9	138032	7.0	29862	1.4	113283	6.0	66974	3.3	5188	0.25	2021	0.09	10718	0.50	4573	0.19
Medium HDI country	317324	28.7	189450	17.3	200389	18.0	123222	11.2	34664	3.2	11932	1.1	70820	6.4	48669	4.5	6238	0.57	3086	0.28	5213	0.47	2541	0.23
Low HDI country	123705	33.0	78745	21.9	89004	23.8	58706	16.3	9871	2.9	3396	1.1	19477	4.9	13590	3.7	1514	0.40	894	0.25	3839	1.00	2159	0.57
Total	1473427	30.3	680372	13.2	662301	14.1	348874	7.1	420368	8.4	97723	1.7	324603	6.7	206956	4.0	18819	0.36	8240	0.15	47336	0.83	18579	0.30

*ASR – age-standardised rates, HDI – human development index

### Incidence and mortality by world region and country

**Figure 2:**
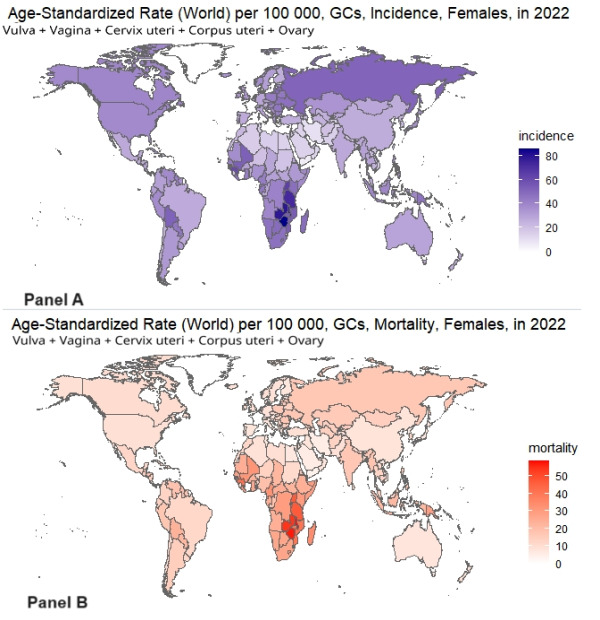
Country-wise age-standardised rates (ASRs) of gynaecological cancer incidence and mortality in 2022. **Panel A** and **Panel B** depict the incidence and mortality rates, respectively, for gynaecological cancers among children across different countries, expressed per 100 000 people. The data highlights significant geographical variations, with certain regions showing markedly higher rates. Understanding these patterns is crucial for tailoring public health interventions to reduce the burden of GCs globally.

The world region with the highest incidence of GCs was Eastern Africa (ASR of 50.7 per 100 000). This was followed by Southern Africa (ASR of 48.5 per 100 000). These two regions have the first and third highest mortality rates respectively, with a mortality rate of 35.3 per 100 000 people in Eastern Africa. The world regions with the lowest incidence and mortality rates were Northern Africa (ASR of 17.1 per 100 000) and Australia and New Zealand (ASR of 8.1 per 100 000) respectively. The high incidence and mortality that occurred due to GCs in some regions was mainly due to the substantial disease manifestations by cervical cancer, such as in Southern Africa and Eastern Africa. **Figure 2** depicts the global incidence (panel A) and mortality rates (panel B) by the GCs in terms of age-standardised rates according to the world standard population per 100 000.

GC incidence and mortality are shown in **Table 1**. The incidence and mortality of GCs in Eswatini (105.4 per 100 000; 71.1 per 100 000) ranked first among all the countries, and Eswatini has the highest incidence of cervical cancer and vulva cancer among all the countries. Yemen has the lowest incidence (5.8 per 100 000) and mortality (4.4 per 100 000) of GCs, and Yemen also has the lowest incidence of cervical cancer (2.1 per 100 000). Switzerland has the lowest mortality rate from cervical cancer (1.1 per 100 000). The countries with the highest and lowest incidence rates of corpus uteri cancer were Samoa (26.2 per 100 000) and Sierra Leone (0.06 per 100 000), respectively.

**Figure 3:**
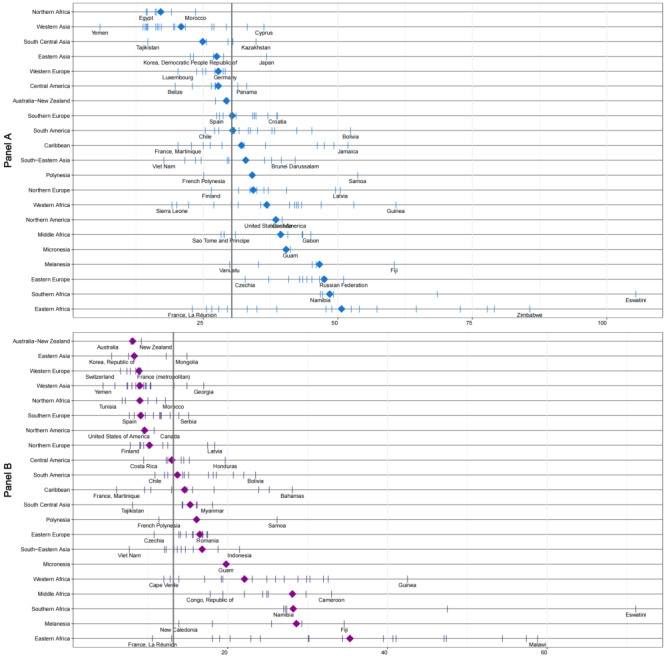
Age-standardised incidence and mortality rates of gynaecological cancers (GCs) in female individuals by world region. **Panel A** and **Panel B**. The top section illustrates the age-standardised incidence rates, while the bottom section presents the mortality rates of GCs among female individuals across different world regions. Diamonds represent incidence (sky blue) and mortality (purple) rates within these regions. Blue vertical bars indicate the incidence rates, and purple vertical bars represent the mortality rates. This figure highlights regional disparities in GC burden, emphasising areas with particularly high incidence and mortality.

Samoa reports the highest mortality rate for uterine body cancer, at 9.5 per 100 000. For ovarian cancer, Latvia has the highest incidence rate at 15.3 per 100 000, while Samoa records the highest mortality rate at 9.3 per 100 000. In contrast, Belize, in Central America, exhibits the lowest incidence (0.69 per 100 000) and mortality (0.78 per 100 000) rates for ovarian cancer. Eswatini shows the highest incidence (5.1 per 100 000) and mortality (3.6 per 100 000) rates for vulvar cancer. Malawi has the highest incidence (1.5 per 100 000) and mortality (0.92 per 100 000) rates for vaginal cancer. These statistics highlight significant regional variations in the age-standardised incidence (**Figure 3**, panel A) and mortality rates (**Figure 3**, panel B) of GCs worldwide.

**Figure 4:**
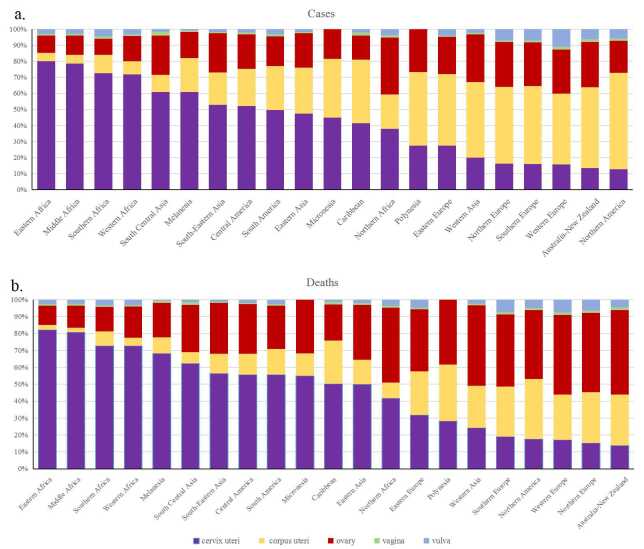
Proportion of different types of gynaecological cancers (GCs) in new Cases and deaths. **Panel A.** The proportion of cervical cancer, corpus uteri cancer, ovarian cancer, vaginal cancer, and vulvar cancer among the total number of new GC cases in 2022. **Panel B.** Proportion of these cancer types in the total number of GC-related deaths. These visual representations highlight the relative prevalence and lethality of each type of gynaecological cancer, providing insight into which cancers contribute most significantly to the overall burden of GCs.

**Figure 5:**
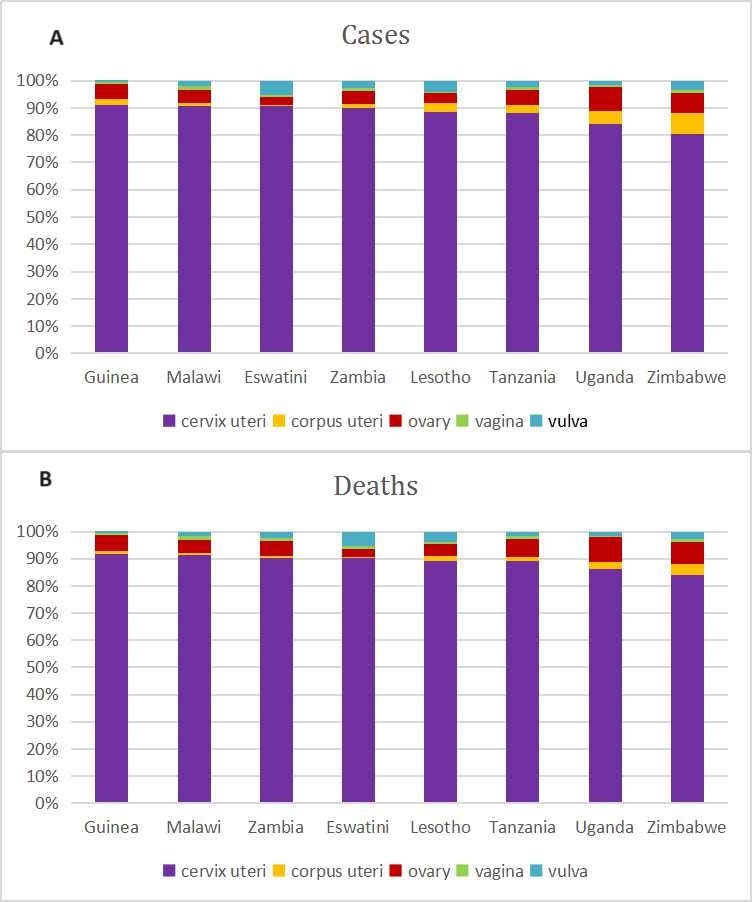
Proportions of various GCs in new cases and deaths in the eight countries with the highest burden. **Panel A**. The proportions of cervical cancer, corpus uteri cancer, ovarian cancer, vaginal cancer, and vulvar cancer among the total number of new GC cases in 2022 for the eight countries with the highest incidence of GCs. **Panel B.** The proportions of these cancer types in the total number of GC-related deaths in the same countries. These visualisations highlight the distribution and impact of different GCs in regions with the highest burden, offering insights into the prevalence and lethality of each cancer type. Understanding these patterns is crucial for directing resources, improving diagnostic and treatment approaches, and formulating public health strategies to combat the most significant contributors to the GC burden in these high-incidence regions.

Regions and countries with a high incidence and mortality of cervical cancer often report a relatively low incidence of corpus uteri cancer (**Figure 4**). The distribution of new cases (**Figure 4**, panel A) and deaths (**Figure 4**, panel B) from GCs – including cervical cancer, corpus uteri cancer, ovarian cancer, vaginal cancer, and vulvar cancer varies significantly across different populations and geographical regions. Cervical cancer consistently shows higher incidence and mortality rates compared to other GCs worldwide. In regions and countries where cervical cancer ranks high in incidence and mortality, corpus uteri cancer ranks comparatively low. Additionally, in the eight countries with the highest number of new cases (**Figure 5**, panel A) and deaths (**Figure 3**, panel B) from GCs, cervical cancer accounts for over 80% of the total cases and deaths.

### Incidence and mortality by HDI

**Figure 6:**
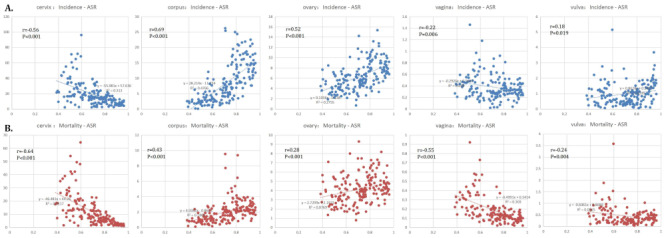
Results of linear correlation analysis between incidence and mortality of each gynaecological cancer and human development index (HDI). **Panel A.** Scatter plot illustrating the linear correlation between the incidence rates of various gynaecological cancers (cervical, corpus uteri, ovarian, vaginal, and vulvar) and the HDI across different countries. Each data point represents a country, showing how HDI is associated with the incidence of these cancers. **Panel B.** Scatter plot depicting the linear correlation between mortality rates of the same GCs and HDI. This analysis provides insights into how socioeconomic factors, as reflected by HDI, influence both the occurrence and lethality of GCs. The findings can help identify patterns and disparities in cancer burden relative to development levels, highlighting areas where public health interventions and resource allocations could be most effective in reducing the incidence and mortality of GCs globally.

Typically, the countries with very high HDI reported the highest incidence rate (ASR of 34.8 per 100 000), and low HDI countries were associated with the second highest incidence rate (ASR of 33.0 per 100 000). The relationship between the incidence and mortality for various GCs and HDI is described in **Figure 6**, panels A–B. The incidence and mortality of cervical cancer are moderately negatively correlated with HDI. Corpus uteri cancer incidence and mortality were positively associated with HDI. The incidence of ovarian cancer shows a moderate positive correlation with HDI. A moderately inverse association between vaginal cancer mortality and HDI was found. There was no significant linear relationship between HDI and morbidity or mortality of vulvar cancer. Furthermore, supplementary materials describe the individual patterns of incidence or mortality of GCs (cervix cancer, Figures S1–4 in the **Online Supplementary Document**), corpus cancer (Figures S5–8 in the **Online Supplementary Document**), ovarian cancer (Figures S9–12 in the **Online Supplementary Document**), vagina cancer (Figures S13–16 in the **Online Supplementary Document**), and vulva cancer (Figures S17–20 in the **Online Supplementary Document**) in different world regions.

**Figure 8:**
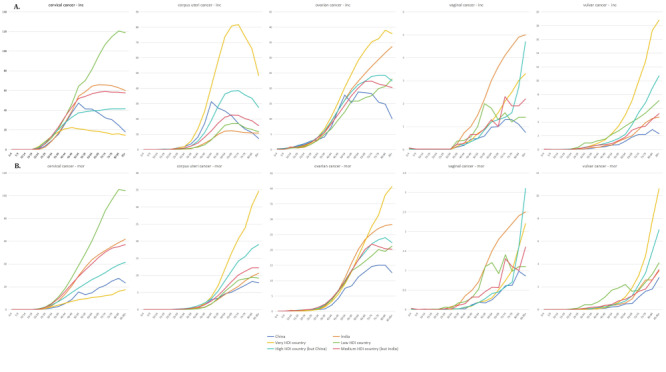
Incidence and mortality rates of gynaecological cancer (GCs) by age-standardized rates (ASR) across different age groups. **Panel A.** The incidence rates of GCs, represented as age-standardised rates (ASR), across various age groups. This chart highlights the distribution of new GC cases among different age brackets, providing insight into which age groups are most affected by these cancers. **Panel B**. Mortality rates associated with GCs, also represented as ASR, across the same age groups. This chart shows the distribution of GC-related deaths among different age brackets, indicating which age groups have the highest mortality risk from these cancers. This information aids for tailoring public health interventions, screening programmes, and treatment strategies to the most affected age groups. Inc – incidence, mor – mortality

We also analysed the incidence and mortality of GCs related to age for different world regions with different levels of HDI (**Figure 8**). The relationship between the incidence or mortality of several GCs depending on HDI grade and age groups was described (**Table 2****,** **Table 3**).

**Table 2:** Incidence rates of gynaecological cancers among patients of different age groups and HDI grades are presented, with each region's highest incidence age group

Age in years	Cervical cancer inc						Corpus carcinoma inc						Ovarian cancer inc						Vaginal carcinoma inc						Vulvar cancer inc					
	**China**	**India**	**Very HDI country**	**Low HDI country**	**High HDI country (but China)**	**Medium HDI country (but India)**	**China**	**India**	**Very HDI country**	**Low HDI country**	**High HDI country (but China)**	**Medium HDI country (but India)**	**China**	**India**	**Very HDI country**	**Low HDI country**	**High HDI country (but China)**	**Medium HDI country (but India)**	**China**	**India**	**Very HDI country**	**Low HDI country**	**High HDI country (but China)**	**Medium HDI country (but India)**	**China**	**India**	**Very HDI country**	**Low HDI country**	**High HDI country (but China)**	**Medium HDI country (but India)**
**0–4**	0.03	0.00	0.00	0.05	0.01	0.00	0.01	0.00	0.00	0.00	0.00	0.00	0.10	0.08	0.14	0.02	0.25	0.14	0.05	0.02	0.05	0.03	0.02	0.07	0.00	0.01	0.01	0.03	0.03	0.05
**5–9**	0.02	0.01	0.00	0.00	0.00	0.00	0.00	0.00	0.00	0.00	0.00	0.00	0.25	0.10	0.39	0.09	0.40	0.13	0.01	0.00	0.00	0.00	0.01	0.02	0.00	0.00	0.00	0.01	0.01	0.00
**10–14**	0.02	0.02	0.01	0.01	0.01	0.00	0.04	0.00	0.00	0.00	0.00	0.00	0.58	0.87	0.70	0.46	0.66	0.70	0.01	0.00	0.00	0.00	0.01	0.02	0.01	0.01	0.00	0.00	0.00	0.00
**15–19**	0.07	0.03	0.05	0.10	0.07	0.08	0.03	0.01	0.04	0.03	0.32	0.04	1.30	0.48	0.87	0.60	1.10	0.88	0.01	0.00	0.00	0.00	0.01	0.02	0.03	0.01	0.01	0.01	0.04	0.01
**20–24**	0.56	0.00	2.90	1.50	2.10	0.97	0.25	0.05	0.25	0.15	0.14	0.11	1.90	0.76	1.20	0.99	1.60	1.40	0.01	0.00	0.00	0.00	0.01	0.02	0.03	0.01	0.05	0.10	0.07	0.03
**25–29**	3.70	2.50	8.20	5.80	7.60	6.20	1.10	0.16	0.87	0.18	0.62	0.52	2.80	1.70	2.10	1.70	2.50	2.30	0.01	0.00	0.00	0.00	0.01	0.02	0.07	0.07	0.14	0.32	0.11	0.17
**30–34**	9.10	8.70	13.70	12.10	13.50	13.90	1.90	0.36	1.90	0.55	0.86	1.00	3.20	3.30	4.00	3.00	4.00	3.90	0.01	0.00	0.00	0.00	0.01	0.02	0.09	0.11	0.30	0.96	0.33	0.27
**35–39**	15.60	16.70	18.00	21.30	20.50	23.00	3.70	0.57	5.60	0.88	2.30	2.30	4.10	5.60	6.70	4.90	6.10	5.50	0.11	0.25	0.14	0.37	0.24	0.23	0.17	0.28	0.58	0.97	0.48	0.48
**40–44**	26.50	27.50	21.20	33.20	27.40	33.60	8.10	2.00	13.50	1.60	5.50	4.20	7.40	9.00	10.70	6.70	9.20	8.40	0.18	0.72	0.28	0.40	0.20	0.26	0.33	0.31	0.95	1.30	0.66	0.64
**45–49**	37.50	39.10	22.20*	46.50	32.80	43.20	17.00	4.10	24.90	3.10	11.00	8.00	12.80	12.70	15.30	9.60	12.80	11.40	0.45	1.00	0.39	0.55	0.34	0.66	0.42	0.55	1.40	1.50	0.89	0.73
**50–54**	47.20*	54.20	21.10	64.30	37.30	51.90	31.30*	6.60	41.40	6.30	19.00	12.20	17.80	16.20	20.30	12.30	16.10	14.50	0.48	1.50	0.70	1.00	0.48	0.66	0.64	0.66	2.30	2.20	1.20	0.86
**55–59**	41.30	58.70	20.10	70.10	38.30	54.00	27.10	9.90	58.00	11.10	28.30	17.20	15.30	19.70	24.70	15.80	18.90	17.60	0.59	2.20	0.89	2.00*	0.85	0.90	0.71	1.20	3.40	2.70	1.60	1.30
**60–64**	41.10	64.30	19.00	81.30	39.30	56.60	25.20	12.00*	73.70	15.80	36.20	21.10	18.80*	22.30	29.10	15.80	21.20	20.10	0.97	3.00	1.20	1.80	1.20	1.30	1.20	1.60	5.00	3.40	2.20	1.80
**65–69**	37.00	65.80*	18.30	95.00	39.70	58.30	22.00	12.30	80.70	16.90*	38.10	22.50*	18.60	24.80	32.40	16.90	22.30	22.20	1.00	3.60	1.60	1.30	1.30	1.00	1.80	2.00	7.10	4.00	3.60	2.80
**70–74**	32.20	65.60	17.20	107.00	40.60	58.90*	17.00	11.60	81.70*	16.90*	38.50*	22.30	18.20	27.20	35.20	17.60	23.90	22.30*	1.30*	4.10	2.10	1.60	1.40	2.30*	2.20	2.90	10.00	4.60	5.40	3.20
**75–79**	30.30	64.80	15.50	114.80	41.40	58.30	13.30	11.10	73.70	14.20	35.60	20.20	15.50	29.40	36.20	19.90	24.20*	21.40	1.30*	4.50	2.50	1.20	1.60	1.90	2.20	3.90	12.80	5.30	6.80	3.40
**80–84**	24.60	62.70	16.00	120.50*	41.30	58.20	11.10	10.80	65.90	13.10	33.50	18.80	14.80	31.60	39.00*	20.60	24.20*	20.90	1.10	4.90	3.00	1.40	2.70	1.90	2.90*	4.50	17.30	6.20	8.90	4.40
**85–85+**	17.90	60.00	14.40	118.90	41.50*	57.60	7.20	10.70	48.30	11.60	27.50	15.60	10.10	33.60*	37.90	23.00*	22.40	20.20	0.75	5.00*	3.30*	1.40	4.70*	2.20	2.30	4.70*	18.80*	7.10*	10.70*	5.20*
**0–85+**	13.80	17.70	9.30	23.80	14.20	18.40	6.80	2.50	15.50	2.90	7.40	4.60	5.70	6.60	8.20	4.90	6.50	6.00	0.23	0.70	0.33	0.40	0.29	0.33	0.32	0.43	1.40	1.00	0.75	0.55

HDI – human development index, inc. – incidence

*The age group with the highest incidence in each region.

**Table 3:** Mortality rates of gynaecological cancers among patients of different age groups and HDI grades are presented, with each region's highest mortality age group

	Cervical cancer inc						Corpus carcinoma inc						Ovarian cancer inc						Vaginal carcinoma inc						Vulvar cancer inc					
	**China**	**India**	**Very HDI country**	**Low HDI country**	**High HDI country (but China)**	**Medium HDI country (but India)**	**China**	**India**	**Very HDI country**	**Low HDI country**	**High HDI country (but China)**	**Medium HDI country (but India)**	**China**	**India**	**Very HDI country**	**Low HDI country**	**High HDI country (but China)**	**Medium HDI country (but India)**	**China**	**India**	**Very HDI country**	**Low HDI country**	**High HDI country (but China)**	**Medium HDI country (but India)**	**China**	**India**	**Very HDI country**	**Low HDI country**	**High HDI country (but China)**	**Medium HDI country (but India)**
**0–4**	0.00	0.00	0.00	0.00	0.00	0.00	0.00	0.00	0.00	0.00	0.00	0.00	0.01	0.03	0.00	0.00	0.07	0.03	0.00	0.01	0.00	0.01	0.00	0.03	0.00	0.01	0.00	0.01	0.00	0.00
**5–9**	0.00	0.01	0.00	0.01	0.00	0.00	0.00	0.00	0.00	0.00	0.00	0.00	0.00	0.04	0.02	0.04	0.08	0.09	0.00	0.00	0.00	0.00	0.00	0.00	0.00	0.00	0.00	0.01	0.00	0.00
**10–14**	0.00	0.01	0.00	0.00	0.01	0.00	0.00	0.00	0.00	0.00	0.00	0.00	0.01	0.33	0.04	0.21	0.17	0.20	0.00	0.00	0.00	0.00	0.00	0.01	0.00	0.01	0.00	0.00	0.00	0.00
**15–19**	0.00	0.02	0.02	0.06	0.08	0.05	0.00	0.00	0.00	0.00	0.00	0.00	0.18	0.18	0.12	0.26	0.37	0.26	0.00	0.00	0.00	0.01	0.00	0.00	0.00	0.00	0.00	0.00	0.00	0.00
**20–24**	0.14	0.01	0.12	0.69	0.31	0.19	0.02	0.01	0.02	0.02	0.03	0.01	0.17	0.29	0.16	0.39	0.48	0.47	0.00	0.01	0.00	0.01	0.00	0.00	0.01	0.00	0.00	0.05	0.01	0.01
**25–29**	0.57	0.87	0.88	2.20	1.90	1.70	0.09	0.04	0.05	0.02	0.17	0.07	0.41	0.65	0.29	0.65	0.53	0.63	0.00	0.02	0.01	0.06	0.01	0.02	0.02	0.01	0.02	0.13	0.04	0.04
**30–34**	1.40	3.40	2.40	5.60	4.10	5.10	0.24	0.08	0.17	0.06	0.26	0.18	0.56	0.59	0.56	0.83	0.96	1.30	0.02	0.08	0.01	0.05	0.02	0.02	0.02	0.02	0.04	0.43	0.09	0.11
**35–39**	2.70	7.50	3.90	11.10	7.10	9.80	0.29	0.13	0.29	0.12	0.53	0.38	0.77	1.70	1.40	2.00	2.10	2.30	0.02	0.12	0.02	0.17	0.02	0.11	0.04	0.06	0.07	0.43	0.10	0.19
**40–44**	5.30	13.70	5.60	18.60	10.80	15.90	0.59	0.46	0.64	0.26	1.10	0.72	2.00	3.70	3.10	3.60	4.10	4.20	0.02	0.35	0.05	0.20	0.06	0.15	0.09	0.07	0.10	0.68	0.17	0.24
**45–49**	8.90	21.00	7.20	28.20	15.00	22.60	1.40	0.69	1.70	0.61	1.90	1.40	4.20	6.80	5.60	6.50	6.80	6.70	0.12	0.49	0.09	0.30	0.10	0.32	0.11	0.38	0.16	1.10	0.24	0.36
**50–54**	15.50	29.60	8.60	38.60	18.90	29.00	2.90	1.70	3.70	1.60	3.20	2.50	7.50	10.90	9.00	9.30	10.00	9.80	0.19	0.71	0.19	0.52	0.18	0.32	0.23	0.36	0.36	1.70	0.38	0.45
**55–59**	13.10	37.10	9.50	49.00	22.60	34.70	3.30	3.00	7.00	3.30	5.40	4.10	8.30	15.80	13.00	13.20	13.40	13.30	0.17	1.10	0.22	1.10	0.28	0.46	0.28	0.44	0.64	1.90	0.53	0.76
**60–64**	14.70	43.70	10.50	60.20	26.30	40.10	4.60	4.40	11.80	5.30	8.40	6.10	11.20	20.20	18.00	14.60	16.70	16.80	0.25	1.50	0.30	1.20	0.41	0.57	0.49	0.57	1.10	2.20	0.74	0.90
**65–69**	18.90	48.10	11.20	73.10	29.10	45.70	5.10	5.60	16.60	6.90	11.30	8.20	12.90	23.40	22.90	16.30	19.30	20.20	0.41	1.80	0.51	0.92	0.45	0.56	0.52	0.93	1.90	1.60	1.20	1.20
**70–74**	21.20	51.80	12.10	86.70	32.40	50.40	6.10	6.80	20.70	8.60	14.20	10.10	14.60	25.40	27.70	18.10	22.00	21.90*	0.61	2.00	0.74	1.40*	0.58	1.30	1.10	1.80	3.00	1.70	2.20	1.60
**75–79**	25.00	55.40	13.00	96.40	36.20	53.90	7.10	8.40	23.90	9.00	15.50	11.20	15.00*	27.00	31.30	20.10	23.30	21.10	0.62	2.20	1.00	0.98	0.70	1.10	1.60	2.60	4.70	2.40	3.20	1.80
**80–84**	27.40*	58.70	16.00	105.30*	39.20	55.20	8.20*	9.70	30.40	9.40*	17.80	12.20*	15.00*	27.90	37.90	19.50	24.00*	20.40	0.98*	2.40	1.60	1.10	1.60	1.00	1.80	2.60	7.80	3.10	5.10	2.60
**85–85+**	23.50	61.70*	17.40*	104.50	41.50*	57.00*	7.90	10.60*	34.60*	9.20	19.00*	12.20*	12.60	28.30*	40.60*	21.20*	22.40	20.20	0.86	2.50*	2.20*	1.10	3.10*	1.60*	2.80*	3.40*	10.60*	4.10*	7.00*	3.50*
**0–85+**	4.50	11.20	3.30	16.30	7.60	11.30	1.10	0.96	2.60	1.10	1.90	1.40	2.60	4.60	4.30	3.70	4.20	4.20	0.08	0.35	0.10	0.25	0.11	0.16	0.14	0.21	0.37	0.57	0.28	0.26

HDI – human development index, inc – incidence

*The age group with the highest incidence in each region.

In countries with a Very HDI, the incidence and mortality rates of uterine corpus cancer, ovarian cancer, and vulvar cancer were generally the highest across most age groups compared to other HDI levels, with uterine corpus cancer being particularly elevated. Conversely, cervical cancer incidence and mortality rates in very high HDI countries were significantly lower than those in other HDI categories, though there is a slight increasing trend in mortality rates with age. Among middle-aged and older women over 45 years old, the incidence and mortality rates of cervical cancer in low HDI countries were notably higher compared to other HDI categories. Additionally, the incidence and mortality rates of vulvar cancer in India were relatively higher compared to countries with varying levels of HDI.

### Incidence and mortality by age

Incidence of GCs was the highest in the age group of 70–74 years (ASR of 114.8 per 100 000). The age category exhibiting the highest mortality was 85 and above (ASR of 91.4 per 100 000). Trends in the age-related changes related to the incidence and mortality of various types of GCs are described in **Figure 8****,** panels A–B, respectively. The incidence of vulva cancer, vagina cancer, and ovary cancer increased with age. The highest incidence of cervix uteri cancer and corpus uteri cancer were reported in the age groups of 60–64 years and 70–74 years respectively. The mortality rate of the individuals diagnosed with GCs attained the highest in the age group of >85 years old except for cervical cancer, which had the highest mortality rate for the age group between 80–84 years.

**Figure 7:**
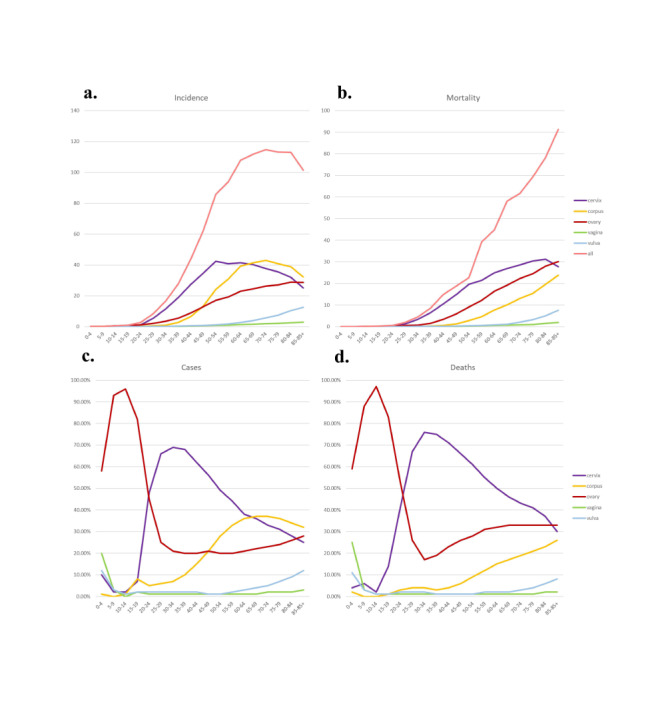
Trends in age-related changes in the Incidence and mortality of gynaecological cancer (GCs) and their various types. **Panel A** and **Panel C.** Line graphs showing the age-related trends in the incidence rates of GCs and their specific types (cervical, corpus uteri, ovarian, vaginal, and vulvar cancers) across different age groups. **Panel B** and **Panel D**. Line graphs illustrating the age-related trends in mortality rates for the same set of GCs. These graphs reveal how the incidence and mortality of each cancer type vary with age, providing a comprehensive view of the age-specific burden of GCs. This detailed analysis highlights which age groups are most affected by each type of GC, offering critical insights for age-targeted prevention, screening, and treatment strategies.

Among all types of GCs, cervical cancer accounted for the largest number of cases and deaths, accounting for 45% of incidence and 51% of mortality **(****Figure 1**, panels B–C). Among women aged 0–19 years, ovarian cancer accounts for the largest number of cases and deaths of all GCs (**Figure 7**). In the age group of 10–14 years, 96% of new GC cases were reported with ovarian cancer whereas 97% of deaths were caused by ovarian cancer among GCs. Among the 20–64 years age group, the number of cervical cancer cases accounted for the largest proportion (**Figure 7****,** panels C–D).

Among women aged 65 years and older, the type of GCs with the highest incidence is cancer of the corpus uteri. In the 25–84 age group, the GCs with the highest mortality rate is cervical cancer. Ovarian cancer was reported for the largest proportion of deaths among the patients >85 years of age group. The incidence and mortality of vaginal cancer were not higher when compared to other types of GCs. However, the proportion of patients with vaginal cancer accounted for 20.00% of all GCs cases among the 0–4 years of age group whereas the proportion of women who died of vaginal cancer in all GCs cases was 25.00% (**Figure 7**, panels A–B).

In North America, Northern Europe, Western Europe, and Australia and New Zealand, the highest incidence of cervical cancer is in the age group before 45 years, indicating that cervical cancer is a cancer of younger age in these regions. With the exception of Northern Africa, the incidence and mortality of cervical cancer among women of all ages is significantly higher in Africa than in other regions. The incidence of cervical cancer in all age groups was significantly lower in Europe than in other regions. In North America area, each age stage uterine body carcinoma incidence of female was obviously higher than that of other areas, with a rate of 117 women aged 65 to 69 years. The incidence of ovarian cancer in all age groups is relatively higher in northern and Western Europe than in other world regions. In Western Europe, the incidence of vulvar cancer among people 85 years of age or older reaches 34.2 per 100 000. Vulvar and vaginal cancer are exceptionally uncommon in Micronesia and Polynesia, whereas corpus uteri and ovarian cancer exhibit elevated age-standardised incidence and mortality rates in these regions. Except for Eastern Europe, mortality rates from cervical cancer are very low in the rest of Europe and in Australia and New Zealand. Interestingly, except for Eastern Europe, the European Region and Australia and New Zealand had significantly higher mortality rates for women of all ages with ovarian cancer. The mortality rate from ovarian cancer in South Africa is 61.7 per 100 000 women older than 85. Except for Micronesia and Polynesia, the mortality rate for corpus uterine cancer in all age groups is less than 50 per 100 000. Except for Melanisa, the mortality of vaginal and vulvar cancer increases with age. Vulvar cancer death rates in Europe and Australia and New Zealand higher relative to other areas. (**Table 4**, **Table 5**)

**Table 4:** Incidence rates of gynaecological cancers among patients of different age groups across various world regions are presented, with the highest incidence age group for each region

	Age	Northern America	Eastern Asia	Eastern Africa	Middle Africa	Northern Africa	Southern Africa	Western Africa	Caribbean	Central America	South-Eastern Asia	South Central Asia	Western Asia	Eastern Europe	Northern Europe	Southern Europe	Western Europe	Australia-New Zealand	Melanesia	South America	Micronesia	Polynesia
Cervix	0–4	0.00	0.03	0.08	0.06	0.00	0.00	0.00	0.24	0.00	0.01	0.00	0.00	0.00	0.00	0.00	0.00	0.00	0.00	0.01	0.00	0.00
	5–9	0.00	0.01	0.00	0.00	0.00	0.00	0.00	0.00	0.00	0.00	0.01	0.01	0.00	0.00	0.00	0.00	0.00	0.00	0.00	0.00	0.00
	10–14	0.03	0.02	0.00	0.00	0.00	0.00	0.00	0.00	0.00	0.01	0.02	0.00	0.00	0.00	0.05	0.00	0.00	0.00	0.00	0.00	0.00
	15–19	0.09	0.07	0.10	0.40	0.04	0.07	0.07	0.12	0.13	0.03	0.03	0.00	0.07	0.07	0.03	0.04	0.00	0.00	0.10	0.00	0.00
	20–24	2.70	0.93	2.60	0.67	0.08	1.50	0.96	4.30	2.00	2.50	0.04	0.08	6.10	6.90	1.20	0.74	0.64	8.00	3.20	0.00	0.00
	25–29	6.30	4.50	10.80	5.30	0.42	9.30	5.10	9.70	9.10	9.20	2.40	0.95	15.10	13.00	5.00	6.10	6.60	20.30	10.40	0.00	4.10
	30–34	9.80	9.90	23.60	14.00	1.60	27.40	8.90	15.50	15.50	17.00	8.10	2.70	23.60	17.10	8.90	10.90	10.70	31.50	17.00	30.90	13.10
	35–39	12.70	16.50	40.40	27.50	4.10	50.70	16.60	21.10	21.60	26.30	15.00	4.80	31.30	18.40*	12.00	13.60	13.30	44.60	23.00	58.40	18.00
	40–44	15.20*	26.80	61.80	44.30	7.70	76.20	28.50	26.30	26.80	35.70	24.10	6.80	35.50	18.30	14.40	15.90*	13.50*	55.60	28.30	39.30	19.90
	45–49	14.80	36.50	84.30	61.30	12.60	91.40	43.60	29.30	30.60	42.40	33.90	9.30	36.90	16.10	15.90*	15.80	12.20	63.20	32.70	40.60	30.10*
	50–54	14.80	49.20*	106.00	78.70	18.00	96.30	60.70	31.00	33.40	46.60	43.00	11.40	37.40*	13.80	15.80	15.20	9.80	66.80	36.30	62.20*	20.80
	55–59	13.40	39.30	123.60	94.70	22.50	97.20	76.30	32.00	35.40	47.50*	49.20	13.00	33.20	12.00	15.10	14.10	8.50	58.80	39.10	37.60	23.00
	60–64	12.20	37.60	138.00	111.80	25.60	95.00	95.40	34.60	37.30	46.70	53.40	13.40	32.20	11.00	14.00	13.00	7.40	53.30	41.90	43.20	28.50
	65–69	11.20	34.20	146.80	124.40	28.20	94.90	120.80	39.00	40.20	45.60	55.30*	14.20	29.80	10.90	13.80	12.60	7.10	58.00	45.10	32.40	27.40
	70–74	10.00	29.00	154.90	135.70	29.50	96.00	149.50	43.60	43.90	44.60	54.80	13.00	29.60	10.80	13.10	12.70	7.10	73.60	47.90	30.40	13.50
	75–79	9.30	26.60	166.50	147.60	31.20	98.10	175.50	49.30	49.70	43.30	52.90	13.40	23.70	11.40	12.20	11.80	7.50	103.40	50.40	53.70	20.40
	80–84	8.50	22.10	179.30*	159.30	32.80*	100.50	200.20	56.20	57.40	40.80	50.90	14.50	23.40	12.00	11.60	12.70	8.20	133.10	52.80	0.00	0.00
	85–85+	7.60	17.60	178.90	171.10*	32.70	105.60*	215.00*	58.30*	65.80*	37.30	48.50	16.20*	17.30	12.10	9.90	11.40	9.00	180.10*	54.00*	0.00	0.00
	0–85+	6.40	13.40	40.40	31.10	6.50	34.90	26.70	14.00	14.30	17.40	15.10	4.10	15.70	8.20	6.40	6.60	5.20	27.60	15.60	18.60	9.60
Corpus	0–4	0.01	0.01	0.00	0.01	0.00	0.00	0.00	0.00	0.00	0.00	0.00	0.00	0.01	0.00	0.03	0.00	0.00	0.00	0.00	0.00	0.00
	5–9	0.00	0.00	0.00	0.00	0.00	0.00	0.00	0.00	0.00	0.00	0.00	0.00	0.00	0.00	0.00	0.00	0.00	0.00	0.00	0.00	0.00
	10–14	0.01	0.04	0.00	0.00	0.00	0.06	0.00	0.00	0.00	0.00	0.00	0.00	0.00	0.00	0.03	0.00	0.00	0.00	0.00	0.00	0.00
	15–19	0.06	0.04	0.00	0.06	0.00	0.00	0.08	0.06	0.05	0.03	0.02	0.02	0.04	0.03	0.00	0.00	0.00	0.00	0.93	0.00	0.00
	20–24	0.39	0.24	0.17	0.08	0.01	0.11	0.17	0.96	0.23	0.19	0.05	0.16	0.23	0.07	0.16	0.08	1.50	0.00	0.06	0.00	0.00
	25–29	0.97	1.10	0.24	0.13	0.11	0.41	0.02	0.61	0.38	1.30	0.23	0.86	0.84	0.48	0.64	0.18	0.69	1.50	0.33	0.00	0.00
	30–34	3.40	2.20	0.58	0.45	0.14	0.43	0.86	1.60	0.77	2.00	0.38	0.86	0.51	1.50	1.20	0.56	1.30	2.30	0.46	15.40	4.40
	35–39	9.60	4.40	0.92	0.63	0.68	0.71	0.67	1.60	1.90	4.30	0.87	2.80	5.20	2.10	3.20	0.74	4.50	4.40	2.20	17.50	13.50
	40–44	20.80	9.60	1.10	1.30	2.30	1.40	0.81	4.20	5.10	7.80	2.40	7.70	15.00	8.20	10.80	4.00	10.20	8.80	5.90	19.60	19.90
	45–49	34.60	18.70	2.40	2.10	4.80	3.00	2.30	12.10	10.20	12.90	4.50	15.40	31.60	17.60	22.50	11.20	19.00	15.50	13.40	20.30	35.10
	50–54	59.40	32.00*	5.50	6.00	8.70	7.30	5.60	26.00	17.80	20.40	7.30	27.00	55.60	34.70	39.80	23.30	33.40	27.50	20.40	41.50	41.60
	55–59	83.80	28.60	10.00	9.50	14.10	15.00	12.50	45.20	27.60	26.50	10.90	39.60	76.40	53.30	55.80	39.00	51.20	42.40	30.00	37.60	57.60
	60–64	106.50	27.50	15.50	11.90	19.60	31.60	19.10	62.60	34.80	28.50*	13.50*	49.70	97.40	70.60	65.00	54.70	67.80	54.60	38.50	43.20	71.40
	65–69	117.00*	24.20	19.30	12.40	21.80*	37.90	21.40	71.20	36.00*	25.90	14.00	53.70*	97.70	85.40	70.00	68.50	79.40	57.10*	42.50	53.90	54.80
	70–74	114.90	20.60	20.50	15.00	21.80*	48.80	24.80*	75.40*	31.20	23.10	12.80	52.00	99.60*	93.30	71.90*	81.50	84.90*	55.60	43.40*	106.40*	80.90*
	75–79	105.40	18.20	21.00	17.20*	20.50	57.40	19.50	75.20	26.60	21.00	11.60	46.90	75.70	98.70*	69.60	80.50	80.80	46.50	42.40	53.70	61.30
	80–84	83.30	16.30	21.50*	15.70	17.90	61.10	21.00	73.70	20.60	18.80	10.70	39.00	68.40	92.70	65.00	84.00*	68.90	27.70	41.30	96.80	0.00
	85–85+	50.70	14.60	20.10	11.40	16.40	63.40*	21.70	66.40	13.60	16.60	9.60	28.80	35.10	79.60	52.90	70.10	49.00	0.00	37.50	0.00	0.00
	0–85+	22.30	7.50	3.00	2.40	3.70	5.90	3.40	12.00	6.70	6.60	2.70	10.10	19.20	14.80	14.00	11.20	14.20	10.70	8.20	14.40	15.50
Ovary	0–4	0.06	0.10	0.05	0.12	0.07	0.15	0.02	0.12	0.00	0.53	0.06	0.14	0.23	0.10	0.07	0.06	0.00	0.00	0.09	0.00	0.00
	5–9	0.23	0.34	0.07	0.16	0.21	0.15	0.09	0.18	0.39	0.69	0.10	0.14	0.22	0.23	0.21	0.20	0.21	0.00	0.20	0.00	0.00
	10–14	0.81	0.58	0.50	0.51	0.65	0.64	0.49	0.47	0.38	1.10	0.70	0.75	0.74	0.58	0.63	0.40	1.50	0.00	0.54	0.00	0.00
	15–19	0.95	1.20	0.62	0.76	0.75	1.10	0.63	0.53	0.68	1.50	0.51	1.20	0.79	0.75	0.86	0.79	0.76	1.10	0.79	0.00	0.00
	20–24	1.20	1.80	0.95	0.88	1.10	1.40	1.20	1.10	1.40	2.40	0.86	1.20	1.40	0.77	1.30	0.78	0.64	2.90	0.98	0.00	0.00
	25–29	2.10	2.80	1.60	1.00	2.00	0.97	1.90	1.40	2.50	3.70	1.80	1.60	3.20	1.50	2.30	0.85	1.50	4.90	1.70	0.00	0.00
	30–34	3.70	3.40	2.60	3.70	3.10	1.80	3.20	4.40	4.20	5.60	3.40	2.60	6.30	3.00	4.40	1.60	2.90	7.30	2.90	15.40	0.00
	35–39	6.00	4.50	4.20	3.70	6.10	2.20	4.50	6.00	6.20	8.30	5.50	4.30	10.70	5.40	6.90	3.30	5.10	10.30	4.60	0.00	4.50
	40–44	9.60	8.10	6.30	7.00	7.80	4.00	6.60	7.10	9.00	12.70	8.40	6.70	16.40	9.00	10.80	6.20	8.40	12.40	6.80	0.00	14.90
	45–49	13.00	13.50	8.80	7.40	11.50	6.00	10.50	8.90	11.90	17.10	11.80	10.70	23.00	13.60	15.60	9.80	12.60	14.80	9.70	20.30	15.00
	50–54	18.10	18.10	12.10	8.70	14.30	8.90	14.70	10.80	14.60	20.80	15.00	15.40	30.30	20.50	20.50	15.30	17.90	16.50	12.90	20.70	31.20
	55–59	22.00	16.10	16.30	11.40	18.90	14.40	19.60*	12.50	17.00	23.50	17.90	19.00	34.30	27.40	25.40	20.90	23.80	17.40	16.30	37.60*	34.60
	60–64	26.30	19.50*	20.70	11.80	19.30	18.40	17.90	14.50	18.80	24.30	20.30	21.80	39.40	35.30	29.10	27.20	29.50	19.90	19.60	25.90	42.80*
	65–69	30.20	19.30	23.40*	15.10	24.90*	21.60	12.90	17.30	20.20	24.30	22.70	22.70	39.60	43.30	32.80	34.10	35.10	22.00	22.40	32.40	36.50
	70–74	32.80	19.40	22.90	17.60	22.30	28.90	14.30	20.40	21.20	24.70*	24.60	23.80	41.30*	47.80	35.50	42.00	39.90	25.00	24.80	30.40	40.40
	75–79	36.00	17.60	22.40	21.80	21.70	34.50	18.00	21.50	21.90	24.40	26.00	27.40	33.90	53.60	37.00	46.80	44.50	28.40	27.10	0.00	20.40
	80–84	37.70*	17.10	23.20	26.60	19.80	37.40	14.30	23.40*	22.00	22.80	27.50	32.00	32.70	55.90	39.40*	58.90	48.40	27.70	29.20	0.00	32.20
	85–85+	37.70*	14.30	23.00	34.20*	17.20	39.80*	17.10	23.40*	22.10*	19.10	28.70*	40.50*	22.10	57.00*	37.00	62.30*	52.00*	36.00*	31.20*	0.00	0.00
	0–85+	7.50	6.00	5.30	4.30	6.00	4.90	5.10	4.90	6.00	8.10	6.10	6.10	11.00	9.10	8.40	7.10	8.00	7.50	5.60	7.30	9.00
Vagina	0–4	0.06	0.05	0.06	0.15	0.03	0.06	0.00	0.00	0.00	0.01	0.03	0.03	0.03	0.10	0.03	0.10	0.10	0.00	0.03	0.00	0.00
	5–9	0.00	0.01	0.01	0.01	0.00	0.00	0.02	0.00	0.00	0.01	0.00	0.00	0.01	0.00	0.00	0.00	0.00	0.00	0.01	0.00	0.00
	10–14	0.01	0.01	0.01	0.01	0.02	0.00	0.00	0.00	0.00	0.00	0.00	0.01	0.00	0.00	0.00	0.00	0.00	0.00	0.00	0.00	0.00
	15–19	0.01	0.02	0.08	0.03	0.03	0.00	0.00	0.00	0.04	0.00	0.00	0.00	0.00	0.00	0.00	0.00	0.00	0.00	0.09	0.00	0.00
	20–24	0.02	0.01	0.04	0.01	0.01	0.00	0.03	0.06	0.03	0.00	0.02	0.00	0.02	0.00	0.00	0.02	0.00	0.00	0.03	0.00	0.00
	25–29	0.03	0.04	0.13	0.06	0.02	0.24	0.04	0.18	0.08	0.01	0.07	0.07	0.01	0.03	0.03	0.02	0.00	0.00	0.05	0.00	0.00
	30–34	0.08	0.07	0.24	0.08	0.02	0.43	0.11	0.31	0.11	0.11	0.13	0.02	0.10	0.06	0.05	0.10	0.09	0.00	0.16	0.00	0.00
	35–39	0.18	0.11	0.55	0.77	0.04	0.96	0.25	0.26	0.31	0.27	0.23	0.03	0.13	0.22	0.04	0.13	0.09	0.00	0.17	0.00	0.00
	40–44	0.35	0.20	0.53	0.32	0.19	0.95	0.63	0.89	0.14	0.13	0.54	0.11	0.28	0.35	0.11	0.26	0.40	0.00	0.27	0.00	0.00
	45–49	0.52	0.41	0.81	1.20	0.31	2.10	0.42	0.98	0.28	0.17	0.87	0.18	0.45	0.44	0.39	0.47	0.60	4.60	0.35	0.00	0.00
	50–54	0.87	0.51	1.40	0.69	0.33	1.60	0.98	1.10	0.55	0.48	1.20	0.24	0.76	0.70	0.54	0.79	1.00	0.00	0.52	0.00	0.00
	55–59	1.20	0.56	1.30	2.40*	1.50	2.10	2.20*	1.80	0.84	0.87	1.80	0.67	0.99	0.88	0.62	0.95	1.30	0.00	0.96	0.00	0.00
	60–64	1.70	0.89	2.40	2.10	1.00	1.90	1.90	2.90	1.20	0.81	2.40	0.76	1.40	1.20	0.82	1.20	1.80	5.80*	1.50	0.00	0.00
	65–69	2.20	1.00	1.80	1.60	1.10	3.00	0.88	3.10	1.80	0.48	2.90	1.10	1.70	1.60	1.50	1.70	2.30	0.00	1.60	0.00	0.00
	70–74	2.70	1.30*	4.00*	1.10	1.10	2.60	0.37	2.20	1.50	1.20	3.40	0.85	2.20	2.00	2.30	2.50	2.50	0.00	2.10	0.00	0.00
	75–79	3.30	1.30*	2.10	2.30	0.98	3.30*	0.55	2.40	2.50	0.78	3.80	1.10	2.10	2.30	3.00	3.30	2.60	0.00	2.10	0.00	0.00
	80–84	3.80	1.20	2.60	1.90	1.60*	2.50	0.62	3.30	2.20	2.60	4.00	1.40	2.50*	2.70	3.60*	4.80*	3.10	0.00	2.70	0.00	0.00
	85–85+	4.10*	1.20	2.40	2.30	1.40	2.40	0.57	5.60*	2.70*	6.10*	4.20*	1.70*	2.00	3.00*	3.50	4.60	3.80*	0.00	4.90*	0.00	0.00
	0–85+	0.43	0.22	0.55	0.50	0.24	0.73	0.35	0.62	0.31	0.22	0.58	0.18	0.34	0.34	0.28	0.38	0.43	0.51	0.35	0.00	0.00
Vulva	0–4	0.01	0.00	0.02	0.12	0.00	0.00	0.06	0.00	0.00	0.02	0.01	0.01	0.00	0.00	0.00	0.00	0.00	0.00	0.06	0.00	0.00
	5–9	0.00	0.00	0.03	0.00	0.00	0.00	0.00	0.00	0.00	0.00	0.00	0.00	0.00	0.00	0.00	0.00	0.00	0.00	0.04	0.00	0.00
	10–14	0.01	0.01	0.00	0.00	0.00	0.00	0.00	0.00	0.00	0.00	0.01	0.00	0.00	0.00	0.03	0.00	0.00	0.00	0.01	0.00	0.00
	15–19	0.03	0.03	0.01	0.01	0.00	0.00	0.00	0.00	0.06	0.00	0.01	0.00	0.00	0.03	0.00	0.00	0.00	0.00	0.09	0.00	0.00
	20–24	0.08	0.03	0.24	0.04	0.00	0.18	0.03	0.00	0.15	0.07	0.01	0.00	0.02	0.10	0.03	0.08	0.11	0.00	0.03	0.00	0.00
	25–29	0.16	0.07	0.71	0.31	0.05	0.66	0.11	0.12	0.04	0.11	0.08	0.06	0.09	0.09	0.03	0.45	0.29	0.00	0.09	0.00	0.00
	30–34	0.42	0.09	1.90	0.45	0.14	2.90	0.68	0.18	0.05	0.21	0.10	0.08	0.21	0.42	0.07	0.81	0.55	0.75	0.28	0.00	0.00
	35–39	1.00	0.16	2.00	0.61	0.07	6.10	0.78	0.26	0.13	0.09	0.27	0.13	0.52	0.78	0.19	1.20	0.99	0.00	0.36	0.00	0.00
	40–44	1.80	0.32	2.50	1.60	0.19	6.30*	0.94	0.22	0.14	0.45	0.28	0.24	0.71	1.60	0.24	2.00	1.50	0.91	0.54	0.00	0.00
	45–49	2.70	0.42	2.50	1.50	0.45	5.40	1.20	0.75	0.31	0.74	0.51	0.45	1.00	2.60	0.59	2.80	2.10	2.10	0.81	0.00	0.00
	50–54	4.00	0.64	3.50	2.40	0.70	3.00	1.90	1.30	0.67	1.20	0.61	0.49	1.80	3.90	1.50	4.20	2.80	0.85	1.10	0.00	0.00
	55–59	5.30	0.73	3.60	3.20	1.40	4.00	3.20	1.40	0.88	1.50	1.10	0.89	2.80	5.20	2.70	5.80	3.60	0.00	1.60	0.00	0.00
	60–64	6.90	1.20	3.50	2.70	2.30	3.30	4.40	1.70	1.70	1.90	1.50	1.50	4.80	6.80	4.80	7.80	4.70	0.00	2.50	0.00	0.00
	65–69	8.80	1.80	3.40	3.80	3.70	3.80	5.70	2.50	3.50	2.80	1.90	2.50	7.40	9.10	8.40	10.90	6.50	2.60*	4.50	0.00	0.00
	70–74	10.90	2.40	2.90	7.70	5.80	4.00	8.10	3.10	4.50	3.20	2.70	3.90	11.00	11.30	12.60	16.80	8.90	0.00	6.70	0.00	0.00
	75–79	13.70	3.00	3.50	5.40	8.30	4.80	10.40	5.00	4.80	4.20	3.60	4.70	12.30	15.00	17.50	21.80	12.20	0.00	7.70	0.00	0.00
	80–84	16.60	3.90	4.80	6.40	9.30	5.40	13.50	6.50	7.80	4.80*	4.20	6.50	16.00*	19.40	20.90*	33.20	16.30	0.00	10.80	0.00	0.00
	85–85+	19.20*	4.20*	7.80*	8.40*	10.70*	4.90	17.10*	8.40*	11.80*	4.80*	4.30*	10.10*	14.70	24.40*	20.60	34.20*	21.10*	0.00	15.70*	0.00	0.00
	0–85+	1.90	0.35	1.30	1.00	0.65	2.10	1.20	0.55	0.53	0.54	0.40	0.46	1.30	1.90	1.30	2.40	1.50	0.35	0.85	0.00	0.00

*The age group with the highest incidence in each region

**Table 5:** Mortality rates of gynaecological cancers among patients of different age groups across various world regions are presented, with the highest mortality age group for each region

	Age	Northern America	Eastern Asia	Eastern Africa	Middle Africa	Northern Africa	Southern Africa	Western Africa	Caribbean	Central America	South-Eastern Asia	South Central Asia	Western Asia	Eastern Europe	Northern Europe	Southern Europe	Western Europe	Australia-New Zealand	Melanesia	South America	Micronesia	Polynesia
Cervix	0–4	0.00	0.00	0.00	0.00	0.00	0.03	0.00	0.00	0.00	0.00	0.00	0.00	0.00	0.03	0.00	0.00	0.00	0.00	0.01	0.00	0.00
	5–9	0.00	0.00	0.00	0.00	0.00	0.00	0.00	0.47	0.00	0.00	0.00	0.00	0.00	0.00	0.00	0.00	0.00	0.00	0.00	0.00	0.00
	10–14	0.00	0.00	0.00	0.00	0.00	0.03	0.00	0.06	0.00	0.00	0.01	0.00	0.00	0.00	0.00	0.00	0.00	0.00	0.01	0.00	0.00
	15–19	0.01	0.00	0.14	0.07	0.00	0.10	0.04	0.00	0.01	0.12	0.01	0.00	0.00	0.14	0.00	0.00	0.00	0.00	0.05	0.00	0.00
	20–24	0.09	0.13	1.30	0.51	0.01	0.18	0.38	0.12	0.04	0.49	0.02	0.02	0.15	0.54	0.13	0.17	0.00	4.50	0.08	0.00	0.00
	25–29	0.60	0.60	4.10	0.90	0.12	5.20	1.80	2.30	1.60	2.00	0.83	0.10	1.90	1.10	0.51	0.58	0.39	9.70	2.70	0.00	0.00
	30–34	1.50	1.50	10.40	5.50	0.26	13.70	3.80	3.30	4.00	4.70	3.20	0.50	5.20	1.80	1.20	1.20	1.20	15.50	5.90	0.00	0.00
	35–39	2.60	2.80	20.70	13.30	0.45	22.80	7.80	6.30	6.80	8.50	6.80	1.60	8.50	2.60	2.10	2.00	1.80	21.70	9.20	0.00	0.00
	40–44	3.80	5.30	34.70	24.30	2.40	33.00	14.20	10.70	10.20	13.30	12.00	2.40	11.50	3.60	3.20	3.10	2.40	27.20	12.50	0.00	14.90
	45–49	5.00	8.70	52.80	38.10	5.30	42.60	22.70	15.30	13.70	18.50	18.30	3.70	14.80	4.20	4.40	4.30	2.90	31.10	15.70	40.60	20.10
	50–54	6.40	14.70	72.90	53.70	9.10	49.50	33.10	17.50	17.10	23.80	25.30	5.40	17.60	4.90	5.60	5.50	3.30	36.40	18.10	20.70	15.60
	55–59	6.70	12.60	92.40	70.90	13.30	55.60	45.00	22.00	20.10	29.00	31.20	7.50	17.60	5.20	6.50	6.40	3.70	41.90	20.40	37.60	17.30
	60–64	6.70	13.70	109.40	89.20	17.40	61.80	59.50	27.20	23.60	34.10	36.60	8.30	19.00	5.90	7.20	6.90	4.20	53.30	23.20	43.20	21.40*
	65–69	6.50	17.50	123.70	107.10	20.80	65.40	79.10	26.10	27.90	38.60	40.60	9.80	19.10	6.90	8.30	7.70	4.80	70.30	27.00	53.90	0.00
	70–74	6.50	19.00	136.70	125.00	23.00	75.30	104.80	34.60	32.70	42.60	43.50	10.40	21.00	8.10	9.50	9.00	5.50	90.30	32.20	76.00	0.00
	75–79	7.70	21.70	153.80	142.00	25.20	98.80	129.90	41.00	38.80	45.00	45.60	12.00	18.70	10.00	10.50	9.70	7.00	121.50	39.50	107.40*	20.40
	80–84	9.30	24.10*	163.70*	157.80	26.90*	132.50	153.10	58.90*	45.80	44.60*	47.90	14.80	20.80*	12.40	13.00	12.60	9.00	149.80	47.70	0.00	0.00
	85–85+	11.20*	22.30	159.60	173.40*	26.90*	179.50*	167.70*	54.90	52.70*	41.30	50.20*	18.40*	17.90	14.50*	14.40*	13.10*	11.60*	192.20*	56.60*	0.00	0.00
	0–85+	2.20	4.30	28.90	22.90	3.80	20.40	16.30	7.70	7.20	9.50	9.50	2.20	6.30	2.20	2.20	2.10	1.40	19.30	7.80	10.90	4.60
Corpus	0–4	0.00	0.00	0.00	0.00	0.00	0.00	0.00	0.00	0.00	0.00	0.00	0.02	0.00	0.00	0.00	0.00	0.00	0.00	0.00	0.00	0.00
	5–9	0.00	0.00	0.00	0.00	0.00	0.00	0.00	0.00	0.00	0.00	0.00	0.00	0.00	0.00	0.00	0.00	0.00	0.00	0.00	0.00	0.00
	10–14	0.00	0.00	0.00	0.00	0.00	0.00	0.00	0.00	0.01	0.00	0.00	0.00	0.00	0.00	0.00	0.00	0.00	0.00	0.00	0.00	0.00
	15–19	0.00	0.00	0.00	0.00	0.00	0.00	0.01	0.00	0.00	0.00	0.00	0.00	0.00	0.00	0.00	0.00	0.00	0.00	0.01	0.00	0.00
	20–24	0.02	0.02	0.02	0.00	0.00	0.00	0.01	0.06	0.01	0.03	0.01	0.03	0.03	0.00	0.00	0.00	0.00	0.00	0.02	0.00	0.00
	25–29	0.05	0.10	0.02	0.00	0.00	0.00	0.00	0.06	0.05	0.31	0.04	0.11	0.04	0.00	0.00	0.00	0.00	0.00	0.07	0.00	0.00
	30–34	0.18	0.24	0.06	0.00	0.01	0.10	0.08	0.37	0.18	0.48	0.09	0.15	0.30	0.00	0.02	0.03	0.18	0.00	0.17	0.00	0.00
	35–39	0.42	0.31	0.10	0.10	0.03	0.11	0.05	0.52	0.49	0.91	0.20	0.26	0.40	0.00	0.08	0.03	0.18	0.81	0.34	0.00	0.00
	40–44	0.85	0.61	0.14	0.17	0.12	0.60	0.09	0.52	0.79	1.70	0.54	0.67	0.98	0.17	0.44	0.23	0.50	0.91	0.69	0.00	5.00
	45–49	1.80	1.50	0.44	0.37	0.30	0.73	0.32	1.50	1.70	2.80	0.86	1.30	2.70	0.85	1.40	0.70	1.00	2.80	1.40	0.00	15.00
	50–54	4.70	3.00	1.20	1.40	0.82	2.00	1.40	3.90	3.20	4.20	1.90	3.00	5.60	2.50	3.10	1.90	2.60	4.20	2.60	20.70	20.80
	55–59	9.00	3.50	2.70	2.70	2.00	5.50	3.40	8.90	5.50	6.00	3.20	5.50	10.10	5.60	5.90	4.00	5.00	10.20	5.10	0.00	5.80
	60–64	15.30	4.90	4.70	3.70	3.60	10.70	6.10	15.50	8.00	7.80	4.80	9.60	17.30	10.70	9.40	7.60	9.20	14.80	9.00	0.00	21.40
	65–69	20.80	5.50	7.10	5.00	5.60	17.30	8.20	22.60	9.80	9.40	6.10	14.30	22.50	17.00	14.00	12.50	15.20	22.00	12.80	32.40	27.40
	70–74	23.50	6.50	9.10	7.50	7.30	23.70	11.60	28.40	11.10*	11.50	7.20	18.60	29.40	24.10	19.30	18.90	20.60	25.00*	15.70	0.00	53.90*
	75–79	28.10	7.70	12.20	9.30	9.10	26.70	11.60	33.40	10.90	13.30	8.30	22.60	28.50	33.10	24.90	24.20	25.90	23.30	17.60	0.00	20.40
	80–84	33.70	9.20	14.10	9.40*	10.70	33.70	12.70	40.50	10.60	14.30*	9.10	26.30	36.40*	42.90	33.00	34.40	31.50	11.10	20.40	96.80*	0.00
	85–85+	39.50*	11.00*	14.60*	7.60	13.60*	47.10*	14.30*	45.50*	9.90	14.30*	9.50*	29.30*	33.20	52.80*	39.40*	41.80*	36.70*	0.00	23.50*	0.00	0.00
	0–85+	3.20	1.10	1.10	0.85	0.82	2.50	1.20	3.40	1.60	1.90	1.00	2.20	3.50	2.60	2.30	2.00	2.30	2.90	2.00	2.50	5.40
Ovary	0–4	0.00	0.01	0.01	0.04	0.01	0.00	0.00	0.00	0.01	0.14	0.02	0.01	0.00	0.00	0.00	0.00	0.00	0.15	0.01	0.00	0.00
	5–9	0.00	0.00	0.03	0.03	0.03	0.00	0.15	0.00	0.01	0.19	0.03	0.01	0.02	0.00	0.00	0.00	0.00	0.00	0.00	0.00	0.00
	10–14	0.02	0.01	0.22	0.25	0.25	0.03	0.15	0.00	0.08	0.33	0.26	0.15	0.05	0.00	0.00	0.02	0.00	0.00	0.09	0.00	0.00
	15–19	0.05	0.18	0.24	0.31	0.67	0.24	0.21	0.53	0.34	0.39	0.20	0.20	0.11	0.00	0.00	0.00	0.00	0.18	0.24	0.00	0.00
	20–24	0.14	0.18	0.36	0.33	0.51	0.36	0.33	0.66	0.64	0.61	0.34	0.27	0.18	0.00	0.00	0.00	0.32	0.19	0.33	0.00	0.00
	25–29	0.34	0.43	0.57	0.35	0.63	0.45	0.62	0.12	0.43	0.68	0.65	0.49	0.38	0.00	0.03	0.02	0.20	0.66	0.55	0.00	0.00
	30–34	0.54	0.59	0.71	1.60	0.69	0.90	0.85	1.80	1.00	1.40	0.66	0.60	0.84	0.31	0.50	0.38	0.36	3.50	0.75	0.00	0.00
	35–39	0.86	0.87	1.60	1.80	1.70	1.20	1.80	2.70	2.30	3.10	1.80	1.70	2.60	0.98	1.40	0.77	0.99	5.40	1.60	0.00	0.00
	40–44	1.90	2.20	3.10	4.00	3.20	2.50	3.70	2.00	4.10	5.70	3.70	3.20	5.40	2.30	2.90	1.80	1.60	8.50	3.00	0.00	0.00
	45–49	3.90	4.40	5.80	5.10	6.20	4.70	7.20	6.50	6.70	8.90	6.50	5.70	9.70	4.50	5.30	3.60	3.30	12.00	5.10	20.30	15.00
	50–54	7.20	7.50	9.50	6.80	9.40	7.80	11.00	6.50	9.20	12.50	10.10	9.20	15.30	7.90	8.30	6.50	6.40	13.10	7.70	20.70	31.20
	55–59	11.00	8.50	14.20	10.10	14.40	12.20	15.90	8.60	12.00	16.20	14.40	13.50	20.20	12.50	12.30	10.60	10.20	18.40	10.60	37.60*	17.30
	60–64	15.50	11.30	18.70	11.40	16.00	16.60	16.00	12.50	14.70	19.70	18.40	18.00	26.80	19.90	16.70	16.70	15.50	20.50	13.80	25.90	21.40
	65–69	20.70	12.90	22.00	15.50	21.80*	21.40	12.90	12.30	16.90	22.40	21.40	22.70	30.20	28.50	21.80	24.10	22.50	21.10	17.20	32.40	27.40
	70–74	26.50	14.40	23.40	19.80	20.40	26.30	15.30	18.50	20.10	24.90	23.30	26.40	35.00	36.30	26.50	32.70	30.60	25.00	20.40	30.40	40.40*
	75–79	33.30	15.30	23.50*	23.40	20.20	34.50	18.30*	20.70	21.60	26.20*	24.20	29.20	31.10	46.20	30.70	39.10	39.70	28.40*	23.60	0.00	20.40
	80–84	38.60	16.30	23.20	27.00	18.40	46.50	12.00	21.60*	22.00	25.20	24.60*	31.70	35.10*	55.40	37.50	53.50	49.50	27.70	26.50	0.00	32.20
	85–85+	42.10*	16.80*	23.20	33.50*	16.20	61.70*	13.70	20.90	22.90*	21.20	24.30	35.50*	28.10	64.50*	40.60*	58.50*	60.30*	36.00	29.10*	0.00	0.00
	0–85+	3.80	2.70	4.20	3.50	4.00	4.20	3.80	3.20	3.90	5.10	4.30	4.30	6.10	4.80	4.10	4.10	4.00	5.80	3.50	6.40	6.00
Vagina	0–4	0.00	0.00	0.03	0.08	0.00	0.00	0.00	0.00	0.00	0.00	0.01	0.00	0.00	0.00	0.00	0.00	0.00	0.00	0.01	0.00	0.00
	5–9	0.00	0.00	0.01	0.01	0.00	0.00	0.00	0.00	0.00	0.00	0.00	0.00	0.00	0.00	0.00	0.00	0.00	0.00	0.00	0.00	0.00
	10–14	0.00	0.00	0.01	0.00	0.01	0.00	0.00	0.00	0.00	0.00	0.00	0.00	0.00	0.00	0.00	0.00	0.00	0.00	0.00	0.00	0.00
	15–19	0.00	0.00	0.03	0.00	0.00	0.03	0.00	0.00	0.01	0.00	0.00	0.00	0.00	0.00	0.00	0.00	0.00	0.00	0.00	0.00	0.00
	20–24	0.00	0.00	0.00	0.00	0.00	0.00	0.02	0.18	0.00	0.00	0.01	0.00	0.00	0.00	0.00	0.00	0.00	0.00	0.00	0.00	0.00
	25–29	0.00	0.00	0.04	0.01	0.00	0.00	0.03	0.31	0.01	0.00	0.03	0.07	0.00	0.00	0.00	0.00	0.00	0.00	0.01	0.00	0.00
	30–34	0.02	0.02	0.07	0.03	0.00	0.20	0.07	0.06	0.00	0.01	0.06	0.00	0.01	0.00	0.00	0.00	0.09	0.00	0.02	0.00	0.00
	35–39	0.02	0.02	0.23	0.38	0.00	0.07	0.14	0.00	0.04	0.04	0.11	0.00	0.00	0.03	0.02	0.00	0.00	0.00	0.04	0.00	0.00
	40–44	0.06	0.03	0.25	0.22	0.05	0.43	0.37	0.15	0.03	0.04	0.27	0.01	0.04	0.06	0.04	0.03	0.10	0.00	0.08	0.00	0.00
	45–49	0.07	0.11	0.41	0.55	0.25	0.36	0.25	0.15	0.12	0.05	0.42	0.03	0.19	0.12	0.08	0.05	0.00	3.50	0.07	0.00	0.00
	50–54	0.14	0.19	0.70	0.31	0.17	0.49	0.52	0.22	0.18	0.17	0.59	0.12	0.42	0.17	0.15	0.14	0.20	0.00	0.24	0.00	0.00
	55–59	0.24	0.16	0.78	1.60	0.63	0.51	1.40*	0.31	0.23	0.39	0.89	0.31	0.30	0.30	0.15	0.19	0.21	0.00	0.22	0.00	0.00
	60–64	0.34	0.23	1.70	1.30	0.34	0.79	1.20	1.60	0.20	0.35	1.20	0.25	0.37	0.42	0.28	0.32	0.44	5.80*	0.34	0.00	0.00
	65–69	0.51	0.38	1.30	1.20	0.38	0.87	0.77	1.30	0.50	0.21	1.50	0.64	0.72	0.63	0.43	0.51	0.51	0.00	0.49	0.00	0.00
	70–74	0.65	0.57	3.40*	1.10	0.31	1.70	0.37	0.89	0.61	0.63	1.60	0.58	1.20	0.90	0.79	0.84	0.72	0.00	0.71	0.00	0.00
	75–79	0.99	0.59	1.80	2.30*	0.77	1.20	0.55	1.00	1.20	0.47	1.80	0.54	1.10	1.40	1.20	1.20	1.30	0.00	0.99	0.00	0.00
	80–84	1.50	0.96	2.20	1.90	1.30*	1.30	0.62	2.10	0.80	1.70	2.00*	1.20	1.40	2.00	1.90	2.20	1.10	0.00	1.60	0.00	0.00
	85–85+	2.50*	1.10*	2.10	2.30*	1.20	5.70*	0.57	4.10*	2.20*	4.20*	2.00*	1.60*	1.70*	2.50*	2.40*	2.60*	2.80*	0.00	2.90*	0.00	0.00
	0–85+	0.10	0.08	0.35	0.31	0.10	0.25	0.22	0.25	0.09	0.10	0.28	0.09	0.13	0.12	0.10	0.10	0.11	0.44	0.11	0.00	0.00
Vulva	0–4	0.00	0.00	0.00	0.00	0.00	0.00	0.03	0.00	0.00	0.00	0.00	0.00	0.00	0.00	0.00	0.00	0.00	0.00	0.01	0.00	0.00
	5–9	0.00	0.00	0.02	0.00	0.00	0.00	0.00	0.00	0.00	0.00	0.00	0.00	0.00	0.00	0.00	0.00	0.00	0.00	0.00	0.00	0.00
	10–14	0.00	0.00	0.00	0.00	0.00	0.00	0.00	0.00	0.00	0.00	0.00	0.00	0.00	0.00	0.00	0.00	0.00	0.00	0.00	0.00	0.00
	15–19	0.00	0.00	0.00	0.01	0.00	0.03	0.00	0.00	0.00	0.00	0.00	0.00	0.01	0.00	0.00	0.00	0.00	0.00	0.00	0.00	0.00
	20–24	0.00	0.01	0.12	0.02	0.00	0.07	0.01	0.00	0.00	0.01	0.00	0.00	0.00	0.00	0.00	0.00	0.00	0.00	0.01	0.00	0.00
	25–29	0.01	0.02	0.27	0.17	0.00	0.41	0.06	0.00	0.01	0.01	0.02	0.03	0.04	0.00	0.00	0.02	0.00	0.00	0.03	0.00	0.00
	30–34	0.03	0.02	0.71	0.28	0.08	1.00	0.40	0.00	0.01	0.08	0.03	0.00	0.10	0.00	0.00	0.05	0.09	0.00	0.03	0.00	0.00
	35–39	0.07	0.04	0.73	0.32	0.00	1.60	0.42	0.00	0.06	0.05	0.07	0.01	0.20	0.03	0.02	0.08	0.09	0.00	0.05	0.00	0.00
	40–44	0.14	0.08	1.20	1.00	0.00	1.80	0.49	0.00	0.05	0.18	0.08	0.00	0.18	0.06	0.04	0.15	0.00	0.30	0.08	0.00	0.00
	45–49	0.28	0.10	1.50	1.50	0.16	2.10	1.20	0.00	0.14	0.20	0.33	0.20	0.15	0.18	0.08	0.21	0.20	0.35	0.18	0.00	0.00
	50–54	0.55	0.23	2.10	2.20	0.30	1.80	1.80	0.22	0.20	0.35	0.36	0.17	0.54	0.48	0.25	0.48	0.20	0.00	0.29	0.00	0.00
	55–59	0.83	0.27	2.40	2.30	0.65	1.80	2.50	0.46	0.33	0.47	0.46	0.47	0.84	0.74	0.52	0.77	0.74	0.00	0.46	0.00	0.00
	60–64	1.20	0.46	2.70	2.10	1.30	1.70	2.50	0.38	0.57	0.62	0.59	0.42	1.90	1.10	0.99	1.20	0.55	0.00	0.77	0.00	0.00
	65–69	1.80	0.50	2.90	0.94	1.30	1.90	1.60	0.47	0.94	0.92	0.86	0.97	3.30	2.10	2.20	2.30	1.40	2.60*	1.20	0.00	0.00
	70–74	3.70	1.50	3.30	2.10	3.70	4.30	3.20	2.00	1.80	1.80	2.20	3.20	6.60	7.40	6.80	7.00	2.60	0.00	3.60	0.00	0.00
	75–79	3.70	1.50	3.30	2.10	3.70	4.30	3.20	2.00	1.80	1.80	2.20	3.20	6.60	7.40	6.80	7.00	2.60	0.00	3.60	0.00	0.00
	80–84	6.50	1.80	4.80	2.60	5.20	7.10	4.60	3.60	4.80	2.60	2.30	3.40	10.30	11.40	10.80	12.40	6.20	0.00	5.60	0.00	0.00
	85–85+	11.40*	3.00*	7.60*	3.80*	10.50*	8.90*	6.80*	4.70*	6.40*	3.30*	2.80*	6.30*	10.40*	16.00*	14.50*	14.50*	11.80*	0.00	8.90*	0.00	0.00
	0–85+	0.10	0.08	0.35	0.31	0.10	0.25	0.22	0.25	0.09	0.10	0.28	0.09	0.13	0.12	0.10	0.10	0.11	0.44	0.11	0.00	0.00

*The age group with the highest mortality in each region.

### Prediction of new cases and deaths in 2050

It is predicted that the number of new GC cases in 2050 will increase by 49.61% when compared to 2022; the number of new GC cases will reach 2 204 390. It is predicted that the number of deaths will increase to 77.23% by 2050, and the number of deaths will reach 1 205 836. Predictions of new incidence of GCs cases and deaths are projected to increase more than 100% by 2050 in Eastern Africa, Middle Africa, Northern Africa, Western Africa, Western Asia, Melanesia (cases: Eastern Africa +164.62.1%, Middle Africa +168.51%, Northern Africa +105.97%, Western Africa +147.11%, Western Asia +108.19%, Melanesia +117.33%; deaths: Eastern Africa +172.30%, Middle Africa +171.42%, Northern Africa +121.00%, Western Africa +151.36%, Western Asia +133.67%, Melanesia +135.08%). Eastern Europe is the only country with a projected decrease in gynaecologic cancer cases (by 3.23%) and the smallest increase in deaths (by 6.24%). The predicted new cases and deaths of GCs in the world regions are shown in **Table 6.**

**Table 6:** Projections of global gynaecological cancer cases and deaths by 2050

	2020		2025				2030				2035				2040				2045				2050			
**World region**	**Cases**	**Deaths**	**Cases**		**Deaths**		**Cases**		**Deaths**		**Cases**		**Deaths**		**Cases**		**Deaths**		**Cases**		**Deaths**		**Cases**		**Deaths**	
	**N**	**N**	**N**	**%**	**N**	**%**	**N**	**%**	**N**	**%**	**N**	**%**	**N**	**%**	**N**	**%**	**N**	**%**	**N**	**%**	**N**	**%**	**N**	**%**	**N**	**%**
Northern America	122 966	38 136	129 480	5.30	40 471	6.12	136 815	11.26	44 479	16.63	142 694	16.04	48 219	26.44	147 449	19.91	51 377	34.72	151 551	23.25	53 801	41.08	155 380	26.36	55 607	45.81
Eastern Asia	352 498	123 834	370 754	5.18	13 3200	7.56	384 866	9.18	145 292	17.33	396 169	12.39	157 143	26.90	399 815	13.42	166 259	34.26	393 553	11.65	171 191	38.24	383 224	8.72	173 441	40.06
Eastern Africa	72 566	47 940	80 615	11.09	53 148	10.86	96 797	33.39	64 053	33.61	115 904	59.72	77 058	60.74	138 109	90.32	92 384	92.71	163 481	125.29	110 177	129.82	192 018	164.61	130 543	172.30
Middle Africa	20 718	13 975	23 544	13.64	15 876	13.60	27 957	34.94	18 888	35.16	33 212	60.31	22 486	60.90	39 470	90.51	26 777	91.61	46 891	126.33	31 879	128.11	55 630	168.51	37 931	171.42
Northern Africa	20 194	10 565	21 993	8.91	11 475	8.61	25 600	26.77	13 578	28.52	29 441	45.79	15 880	50.31	33 435	65.57	18 313	73.34	37 506	85.73	20 819	97.06	41 593	105.97	23 349	121.00
Southern Africa	17 016	9775	18 621	9.43	10 981	12.34	20 796	22.21	12 390	26.75	22 819	34.10	13 808	41.26	24 764	45.53	15 236	55.87	26 697	56.89	16 690	70.74	286 09	68.13	18 143	85.61
Western Africa	43 552	25 158	48 627	11.65	28 174	11.99	57 090	31.08	33 138	31.72	67 098	54.06	39 036	55.16	78 750	80.82	45 935	82.59	92 187	111.67	53 946	114.43	107 622	147.11	63 237	151.36
Caribbean	9699	4757	10 197	5.13	5002	5.15	11 119	14.64	5546	16.59	11 967	23.38	6081	27.83	12 731	31.26	6581	38.34	13 401	38.17	7049	48.18	13 956	43.89	7431	56.21
Central America	28 952	13 717	30 539	5.48	14 593	6.39	34 546	19.32	16 822	22.64	38 605	33.34	19 189	39.89	42 579	47.07	21 615	57.58	46 310	59.95	23 999	74.96	49 674	71.57	26 262	91.46
South-Eastern Asia	13 1807	68 407	14 0421	6.54	74 375	8.72	153 628	16.56	83 549	22.14	165 837	25.82	92 477	35.19	176 732	34.08	100 806	47.36	186 067	41.17	108 274	58.28	193 814	47.04	114 786	67.80
South Central Asia	25 2898	15 3768	27 7726	9.82	170 356	10.79	316 498	25.15	196 477	27.77	356 951	41.14	224 381	45.92	397 958	57.36	253 436	64.82	43 8332	73.32	282 911	83.99	476 821	88.54	31 2010	102.91
Western Asia	28 420	12 470	30 955	8.92	13 545	8.62	36 236	27.50	16 153	29.53	41 876	47.35	19 102	53.18	47 701	67.84	22 312	78.93	53 507	88.27	25 686	105.98	59 168	108.19	29 139	133.67
Eastern Europe	12 7647	52 175	12 7438	-0.16	52 203	0.05	129 316	1.31	54 102	3.69	129 571	1.51	55 376	6.14	128 613	0.76	55 974	7.28	126 561	-0.85	55 928	7.19	123 519	-3.23	55 432	6.24
Northern Europe	34 971	14 037	36 018	2.99	14 661	4.45	37 794	8.07	15 799	12.55	39 359	12.55	16 863	20.13	40 679	16.32	17 761	26.53	41 756	19.40	18 576	32.34	42 635	21.92	19 297	37.47
Southern Europe	48 925	19 686	49 893	1.98	20 190	2.56	51 583	5.43	21 424	8.83	52 720	7.76	22 637	14.99	53 250	8.84	23 710	20.44	53 090	8.51	24 555	24.73	52 262	6.82	25 044	27.22
Western Europe	61 837	25 631	63 378	2.49	26 545	3.57	66 189	7.04	28 211	10.07	68 675	11.06	299 26	16.76	70 631	14.22	31 479	22.82	71 895	16.27	32 691	27.54	72 368	17.03	33 387	30.26
Australia/New Zealand	7714	2770	8288	7.44	3021	9.06	9046	17.27	3418	23.39	9758	26.50	3820	37.91	10 421	35.09	4195	51.44	11 032	43.01	4537	63.79	11 602	50.40	4855	75.27
Melanesia	2204	1280	2522	14.43	1447	13.05	2940	33.39	1718	34.22	3385	53.58	2016	57.50	3846	74.50	2333	82.27	4317	95.87	2667	108.36	4790	117.33	3009	135.08
South America	88 596	42 171	93 206	5.20	44 325	5.11	103 568	16.90	50 156	18.93	113 707	28.34	561 47	33.14	123 258	39.12	62 048	47.13	131 890	48.87	67 634	60.38	13 9347	57.28	72 751	72.51
Micronesia	120	60	124	3.33	65	8.33	134	11.67	71	18.33	143	19.17	77	28.33	150	25.00	81	35.00	159	32.50	84	40.00	165	37.50	87	45.00
Polynesia	127	60	141	11.02	67	11.67	155	22.05	75	25.00	167	31.50	82	36.67	178	40.16	88	46.67	186	46.46	92	53.33	193	51.97	95	58.33

**Figure 9:**
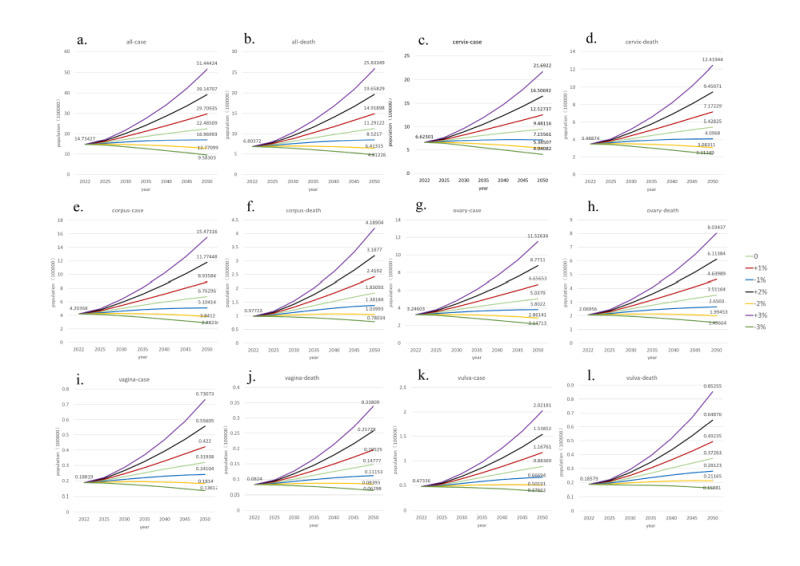
Projected new cases and deaths of different categories of gynaecological cancers in 2050. **Panels A**, **C**, **E**, **G**, **I**, and **K.** The forecasted number of new cases for various categories of gynaecological cancers in 2050. These categories include cervical cancer, corpus uteri cancer, ovarian cancer, vaginal cancer, and vulvar cancer. Each chart provides a detailed breakdown, highlighting the expected incidence rates and the distribution of new cases among these cancer types. **Panels B**, **D**, **F**, **H**, **J**, and **L.** The projected number of deaths related to the same categories of gynecological cancers in 2050. These charts offer a comprehensive overview of mortality rates, emphasising the predicted death toll and the relative contribution of each cancer type to the overall mortality burden.

**Figure 10:**
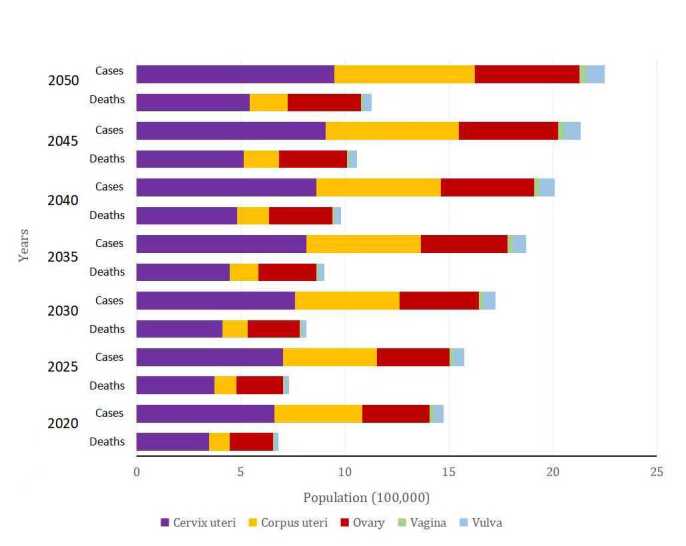
Projections of incidence and mortality rates of each gynaecological cancer (GC) in 2050. the projected incidence and mortality rates for various types of gynaecological cancers by 2050. The data highlights the anticipated significant increase in the burden of these cancers over the next three decades. The significant expected rise in both incidence and mortality rates underscores the need for enhanced research efforts, improved screening programmes, and innovative therapeutic approaches to mitigate the impact of gynaecological cancers on global health.

Number of new cases of GCs and related deaths is predicted to increase by 2050. Projections of the annual rates of change for seven scenarios (with annual rates of change for ASR ranging from −3% to +3%) by 2050 were reported; the annual number of GCs and deaths is likely to increase under the −1%,0%, +1%, +2% and +3% scenarios, and decrease in the remaining scenarios (**Figure 9**). Projections show that the world burden of GCs incidence (**Figure 9**, panels A, C, E, G, I, and K) and mortality (**Figure 9**, panels B, D, F, H, J, and L) will be reduced only when the incidence and mortality of GCs are reduced by 2% and 3%, respectively. The number of new cases and deaths of vulvar cancer has increased except for -3%. In 2050, the number of deaths from corpus uteri and vaginal cancers will decrease if the mortality rate were 3% lower than in 2022. The number of new cases and deaths of cervical cancer and ovarian cancer will decrease if the relevant rate were 3% lower than in 2022. And the number of new deaths of corpus uteri cancer and vaginal cancer all increased except −2 and −3%. (**Figure 9**) In the absence of any changes in the incidence and mortality rates of each GC in 2050, the burden is expected to significantly increase over the next three decades (**Figure 10**).

## DISCUSSION

In the year 2022, a total of 1 473 427 women were diagnosed with GCs and 680 372 died of GCs. The incidence of GCs was 30.3 per 100 000 and the mortality was 13.2 per 100 000. In 2022, the number of new cases and deaths related to the GCs accounted for more than 15% of all cancer cases and deaths in women. These data indicate that women with GCs represent a larger group. GCs not only severely affect women's physical and mental health, but also affect their overall quality of life [17]. This statistic highlights the significant subgroup of women with GCs, emphasising the critical need for primary prevention to mitigate incidence and improve morbidity outcomes, which has a profound impact on women's health and quality of life.

Obesity's relationship with GCs is multifaceted, with a substantial body of evidence supporting its association with an increased risk of several cancer types [18–20]. A recent survey by a Korea national health agency suggested that a high BMI and diabetes typically increased the risk of cervical cancer [21]. Increased BMI is associated with a higher risk of endometrial cancer; there is a positive association between proinflammatory biomarkers associated with obesity and an increased risk of endometrial cancer development [22]. Women who are overweight in midlife are more likely to develop uterine corpus cancer in later life [23]. Obesity is also a risk factor for vaginal cancer [24]. Vulvar cancer with obesity has a shorter time to recurrence [25]. Treatment of obesity and diabetes has been suggested as interventions to prevent ovarian cancer and improve its outcomes [26]. Moreover, the inflammatory milieu associated with obesity is implicated in the carcinogenesis of endometrial cancer, suggesting a direct relationship between obesity-driven inflammation and cancer risk [27]. However, a few studies have reported that women with obesity may have lower cervical cancer screening rates and obese women are less likely to attend cervical cancer screening and more likely to have sub-optimal test results than normal-weight women [28,29]. Therefore, it is important to enhance adherence to screening in obese women for diagnosing GCs at an early stage.

Among all GCs, cervical cancer has the highest morbidity (14.1 per 100 000) and mortality (7.1 per 100 000). In 2022, 662 301 women were suffering from cervical cancer, and 348 874 women died of cervical cancer. Human papillomavirus (HPV) is a significant cause of almost all cervical cancers. Women infected with HIV/AIDS are particularly at a high risk of developing cervical cancer due to an impaired immune response [30]. Genital tract HPV infection leading to cervical intraepithelial neoplasia (CIN) could induce the development of cervical cancer [31]. The vaginal microbiome can greatly influence the natural history of HPV infection and its clinical impact [32]. Smoking is also related with cervical cancer. One mechanism is a local immune suppression caused by tobacco metabolite, which may adversely affect the host's ability to develop an effective immune response against viral infections, increasing the risk of persistent cervical infections. Additionally, nicotine and its metabolites such as cotinine have been found in cervical mucus of female smokers, which can cause DNA damage in squamous epithelial cells [33]. Several factors, including hormone levels, hygiene practices, and sexually transmitted diseases (STDs), can disrupt the natural balance inside the vagina [32]. According to the risk factors displayed on the website of the MD Anderson Cancer Center, the development of vaginal and vulvar cancers shares some same risk factors with cervical cancer, with one of them being a history of cervical cancer. Vulvar cancer and vaginal cancer have also been reported to be associated with co-infection of HPV and HIV infection [34, 35]. The success of HPV vaccines in preventing cervical and other GCs highlights the importance of vaccination programmes, which have led to a 65% decrease in cervical cancer incidence among vaccinated cohorts in the United States [36]. Reports have proposed that HPV vaccines are highly effective in preventing the malignant diseases of the cervix, vulva, and vagina caused by vaccine-specific HPV types [37,38].

The risk factors for corpus uteri and ovarian cancers are closely associated with pregnancy and parity. Progesterone, which is produced in large quantities during pregnancy, exerts a protective effect on the endometrium. Parous women have a 40% lower incidence of corpus uteri cancer compared to nulliparous women [39]. Multiple studies indicate that pregnancy also has a protective effect against ovarian cancer. The risk of ovarian cancer is reduced in women who have experienced live births or induced abortions, with the degree of risk reduction being strongly correlated with parity [40,41].

The health care infrastructure in various regions significantly influences the incidence and mortality rates of GCs. Factors such as access to screening, specialised treatment, health care education, and research into new treatments collectively impact these rates. Regions with robust health care infrastructure typically exhibit lower incidence and mortality rates, attributable to a comprehensive approach encompassing prevention, early detection, and effective treatment of GCs [42]. Implementing comprehensive and effective preventive measures can significantly decrease the incidence of various GCs. For cervical cancer, administration of the HPV vaccine has been shown to markedly reduce its incidence. Additionally, HPV vaccination can lower the incidence of vaginal and vulvar cancers. Hence, HPV infection screening and vaccination are critical strategies for reducing the incidence of GCs [42].

Regarding ovarian cancer, risk-reducing salpingo-oophorectomy is recognised as the most clinically effective preventive method. Current evidence indicates that screening for ovarian cancer does not significantly reduce mortality rates associated with the disease. Nutritional factors also play a role in the risk of GCs. Studies suggest that higher relative intake levels of fats, vitamin C, and copper may increase the risk of endometrial cancer, whereas higher intake levels of carbohydrates or sugars may decrease this risk. These findings provide valuable dietary guidelines for individuals at high risk of endometrial cancer, potentially reducing the likelihood of cancer development [43,44].

Early diagnosis is pivotal for reducing mortality and improving prognosis in various types of GCs. In recent years, advancements in artificial intelligence (AI) technologies have made significant contributions to the diagnosis and treatment of these tumours. Studies indicate that deep learning and machine learning models enhance the accuracy of cervical cancer diagnosis and can be effectively utilised for screening purposes [45,46]. Furthermore, certain models can predict clinical benefits or the optimal time for cessation of treatment in ovarian cancer [47]. Researchers have developed AI-based diagnostic prediction tools that are low-cost, accessible, and accurate for diagnosing ovarian cancer. Additionally, AI has been clinically applied to digital pathology slides of gynaecologic tumours, where deep learning models demonstrate potential in accurately diagnosing these tumours, classifying histological subtypes, and predicting treatment response and prognosis. The role of AI in precise risk stratification and comprehensive management of gynaecologic cancer patients is increasingly important, providing robust support for improving patient survival rates and quality of life. Consequently, AI offers significant assistance in screening and diagnosing gynaecologic tumours, thereby aiding in reducing the global burden of these cancers [48–50].

Furthermore, in our study, South Africa, has consistently reported a higher incidence of GCs, including cervical, ovarian, and uterine cancers. This phenomenon can be attributed to several factors, including a high prevalence of HPV infection, limited access to health care services, and socioeconomic disparities. Southern African countries, like South Africa, have recognised the urgency of implementing comprehensive cervical cancer prevention programmes, such as HPV vaccination and cervical cancer screening, especially for women living with HIV. Eastern Africa has also shown a substantial burden of GCs, particularly cervical cancer. The prevalence of HPV and limited access to early detection and treatment services are among the key contributors to the high incidence in this region. Efforts to address these challenges, such as expanding HPV vaccination programmes and increasing access to screening, have been ongoing, but there is still work to be done to bridge the gap in gynaecological cancer outcomes. Conversely, Northern Africa stands out with relatively lower incidence rates for GCs, including cervical cancer. Factors contributing to this disparity may include differences in lifestyle, sexual behaviour, and regional variations in HPV prevalence. The lower incidence rates in Northern Africa provide an intriguing area for further investigation to uncover the specific factors that contribute to this phenomenon.

In Central Africa, the incidence of GCs, particularly cervical cancer, is notably higher. This region faces challenges such as limited access to health care services, lack of awareness about preventive measures, and a higher prevalence of HIV, which increases the risk of GCs among women living with the virus. Eswatini's exceptionally high incidence and mortality rates of GCs, especially cervical cancer, are closely linked to its severe HIV epidemic. HIV-positive women are at a significantly higher risk of developing cervical cancer, and this country's health care system faces challenges in providing comprehensive care and treatment for both HIV and GCs. Australia and New Zealand report relatively low gynaecological cancer incidence rates, which can be attributed to well-established health care systems, comprehensive cancer screening programmes, and public health initiatives. These countries have also made significant progress in HPV vaccination efforts, which have contributed to reducing the burden of cervical cancer.

In addition, we have analysed eight countries with the top ten age-standardised incidence and mortality rates for GCs worldwide. In these countries, which are classified as low and medium HDI, and low- and middle-income countries, cervical cancer-related cases and deaths constitute over 80% of all GCs. According to WHO statistics, there are significant disparities in HPV vaccine coverage, with some low-income countries reporting as low as 5% coverage among eligible girls, compared to approximately 90% coverage in many high-income countries [51]. This disparity in vaccine coverage exacerbates existing health and economic inequalities.

Women face particular barriers to accessing the HPV vaccine, despite bearing a higher burden of HPV-related diseases. Furthermore, women often serve as primary caregivers within families, dedicating more time to unpaid care work compared to men. The greater burden of community disease on women, combined with their caregiving responsibilities, can limit their employment opportunities, financial security, and educational attainment, thereby exacerbating existing social inequalities [52]. The WHO's advocacy for a single-dose HPV vaccine could help alleviate this disease burden and address inequalities rooted in gender norms. Unlike multiple-dose vaccines, single-dose vaccines reduce pressure on vaccine supply, lower production costs, and are easier to distribute. This can enhance the availability of HPV vaccines in low and medium HDI countries, facilitating vaccine delivery to hard-to-reach populations and increasing coverage. Improved HPV vaccine coverage can significantly reduce the disease burden caused by HPV infection and mitigate the social inequalities arising from gender norms [52].

In addition, we have selected eight countries with top ten age-standard incidence and mortality rates for GCs worldwide. Cervical cancer-related cases and deaths account for more than 80% among all GCs in these countries, which are classified as low and medium HDI countries and also low- and middle-income countries. According to statistics published by WHO, there are significant disparities in the coverage of HPV vaccines, with some low-income countries having as low as 5% coverage of HPV vaccine among eligible girls, while many high-income countries have approximately 90% coverage [51]. This vaccine inequality further exacerbates the existing health and economic disparities in these countries. The barrier of women to access HPV vaccine should be especially concerned, as they carry a higher burden of HPV-related diseases. Moreover, this is not the only way women are disadvantaged in the lack of HPV vaccine. In many communities, women are the primary caregivers within families, ‘spending more time on unpaid care work than men’. Under the greater burden of community disease compared with men, women's additional caregiving responsibilities may limit their work, financial security and education, exacerbating existing social inequalities [52].

The incidence and characteristics of GCs vary significantly across different age groups, reflecting distinct patterns and challenges within each age bracket as evidenced by our analysis of the GLOBOCAN data. In the 0–19 age group, ovarian cancer emerges as a prominent concern, constituting the largest proportion of GC cases in this demographic. Notably, the 10–14 age group reports a higher incidence of new ovarian cancer cases, highlighting ovarian cancer's predominance among female adolescents. Unfortunately, the mortality associated with ovarian cancer in the 0–19 age group is the highest among all GCs. Despite its relative rarity, ovarian cancer ranks seventh in incidence and fifth in mortality among women aged 0–19 years. This can be attributed, in part, to the limitations of medical technology, which often lead to late-stage diagnoses and subsequently poor prognosis for ovarian cancer patients [53,54]. As such, there is a critical need for the identification of appropriate tumour markers to enable early diagnosis and intervention in this vulnerable age group.

Cervical cancer is most prevalent among the 20–59 age group, highlighting its significance in young and middle-aged women. In contrast, corpus cancer takes precedence in the above 60 age group, underscoring the shift in the predominant GC type with advancing age. Cervical cancer also stands out as the leading cause of GC-related deaths in the 20–79 age group, ranking third among all cancer types in this demographic. In the 80 and above age group, ovarian cancer accounts for the highest proportion of GC-related deaths, presenting distinct age-specific challenges in terms of diagnosis and treatment. Endometrial cancer generally exhibits a relatively favourable prognosis; however, the outlook for patients with primary metastatic/recurrent endometrial cancer is notably poor [53,55]. This underscores the importance of early detection and intervention for this subset of patients. Vulvar and vaginal cancers are considered rare GCs in the broader context [56,57]. Nonetheless, among children aged 0–14 years, these two cancers occupy the second and third positions in terms of the number of cases among all GCs, respectively. Rhabdomyosarcomas originating from the vagina stand out as the most common vaginal cancer in paediatric and adolescent patients, emphasising the unique nature of GCs in this age group [58].

These important clinical variations in the incidence, type, and prognosis of GCs across age groups underscore the importance of tailoring screening, prevention, and treatment strategies to the specific needs of different age cohorts. Moreover, ongoing research efforts are crucial to further elucidate the underlying factors contributing to these age-related disparities and to improve outcomes for all women affected by GCs.

Low HDI regions have been consistently reported to exhibit a higher incidence rate of GCs, as evidenced by data from the GLOBOCAN database. In these regions, the ASR reaches 33.0 per 100 000, which is the second-highest incidence rates, underscoring the profound burden of GCs on populations with limited access to health care and socioeconomic resources. Intriguingly, very high HDI regions, although generally characterised by superior economic conditions and health care infrastructure, demonstrate the highest incidence rates, with an ASR of 34.8 per 100 000 for GCs. This finding suggests that the relationship between HDI and GC incidence is complex, and influenced by a multitude of factors beyond economic prosperity [59]. Countries classified as having very high HDI levels exhibit a unique juxtaposition of relatively high incidence rates and notably low mortality rates for GCs. This phenomenon can be attributed, in part, to their robust economies and well-developed health care systems, which enable earlier detection and access to advanced treatment options. The interplay between HDI and GC outcomes highlights the critical role of socio-economic factors in shaping the epidemiology of these cancers [59].

Conversely, low HDI countries face a starkly different scenario, marked by the highest incidence rates of GCs. Notably, this disparity is closely linked to a higher prevalence and incidence of HIV/AIDS and HPV infections with poor uptake of vaccinations within these regions when compared to medium, high, and very high HDI countries. The synergistic impact of HIV/AIDS and HPV infections on the incidence of GCs further accentuates the pressing need for targeted interventions and health care infrastructure development in low HDI settings to mitigate the disproportionate burden of these diseases [60]. It is important to note that the relationship between HDI and the incidence and mortality of each specific type of gynaecological cancer varies considerably. Factors such as access to health care, awareness of preventive measures, and regional variations in risk factors contribute to this variability. Consequently, a nuanced understanding of the unique dynamics driving GCs within different HDI contexts is essential for tailoring effective public health strategies and interventions to address the specific challenges faced by each region. Further research and analysis are warranted to explore these intricate relationships and inform targeted approaches to reduce the incidence and mortality of GCs worldwide.

Future projections of the GCs burden in the world were estimated and the prediction is that the incidence and mortality of GCs are assumed to be the same as in 2022, and only population structure affects the results. In 2050, the number of new cases and deaths in Eastern and Middle Africa is predicted to be higher. Therefore, it is necessary to examine the incidence of GCs in Eastern and Middle Africa. We also assumed six scenarios of change in the incidence and mortality, with annual rates of change for ASR ranging from −3 to +3%. New cases and deaths of GCs in 2050 can only be reduced if the incidence and mortality rates are reduced by 2 and 3%, respectively. The number of new cases and deaths of cervical cancer, and the number of new cases of corpus uteri cancer and ovarian cancer all increased except −2 and −3%. These indicators can suggest the necessary steps to be taken to mitigate morbidity and mortality associated with new GC incidence rates in the future on time. It is important to note that these estimates must be interpreted with caution given the current limited quality and coverage of cancer data worldwide. These updated estimates of cancer burden related to GCs can provide a crucial reference for setting health policy priorities subsequently advancing and expediting initiatives for cancer control to alleviate the existing and anticipated burden by the year 2050. Primary prevention is the key to reducing the incidence of GCs, while screening and early detection should be executed on time with utmost attention to high-risk groups depending on age and HDI.

## CONCLUSIONS

This study highlights significant global inequalities in the incidence and mortality of GCs in 2022, based on data from The Global Cancer Observatory (GLOBOCAN). With 1 473 427 new cases and 680 372 deaths reported, the incidence rate observed as 30.3 per 100 000, and the mortality rate was 13.2 per 100 000. The highest incidence rates observed in Eastern Africa, while the highest mortality rates found in East Africa, regions also heavily affected by HIV and HPV. Conversely, Australia and New Zealand had the lowest mortality rates. Countries with very high Human Development Index (HDI) exhibited the highest incidence of GCs, followed closely by low HDI countries. Notably, Eswatini recorded the highest incidence and mortality rates, whereas Yemen had the lowest. Projected trends indicate a rise in new cases and deaths from gynaecological cancers over the next two decades if current morbidity and mortality patterns persist. These findings conclude the urgent need for targeted public health interventions and equitable health care resources to address the disparities in gynaecological cancer outcomes worldwide.

## Additional material


Online Supplementary Document

**Correspondence to:** Prof Junqi Liu, MD, Ph.D. Cancer Center, The First Affiliated Hospital of Zhengzhou University No. 1, Jianshe East Road, Erqi District, Zhengzhou, Henan Province PR China fccliujq@zzu.edu.cn Prof Narasimha M Beeraka, MRes, Ph.D. Department of Human Anatomy and Histology, I.M. Sechenov First Moscow State Medical University (Sechenov University) 8/2 Trubetskaya Street, Moscow Russian Federation bnmurthy24@gmail.com biraka_n@staff.sechenov.ru
