# Supplementary material for: Global burden of gynaecological cancers in 2022 and projections to 2050
Source: J Glob Health. 2024 Aug 16;14:04155. doi: 10.7189/jogh.14.04155 (PMC11327849; doi:10.7189/jogh.14.04155)
Supplement: Online Supplementary Document [file jogh-14-04155-s001.pdf]

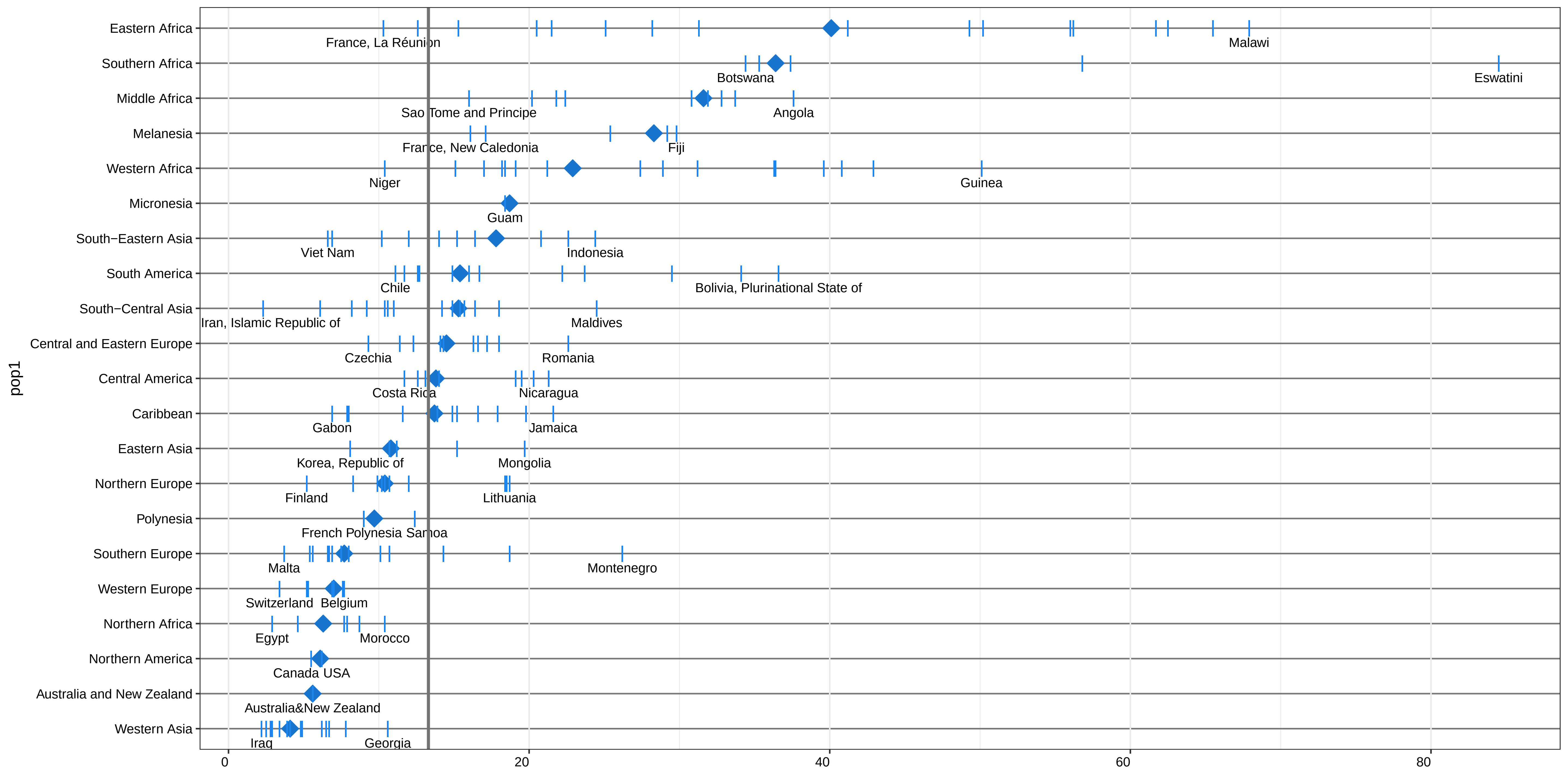

Eastern Africa
Southern Africa
Middle Africa
Melanesia
Western Africa
Micronesia
South−Eastern Asia
South America
South−Central Asia
Central and Eastern Europe
Central America
Caribbean
Eastern Asia
Northern Europe
Polynesia
Southern Europe
Western Europe
Northern Africa
Northern America
Australia and New Zealand
Western Asia
pop1
France, La Réunion
Malawi
Botswana
Eswatini
Sao Tome and Principe
Angola
France, New Caledonia
Fiji
Niger
Guinea
Guam
Viet Nam
Indonesia
Chile
Bolivia, Plurinational State of
Iran, Islamic Republic of
Maldives
Czechia
Romania
Costa Rica
Nicaragua
Gabon
Jamaica
Korea, Republic of
Mongolia
Finland
Lithuania
French Polynesia Samoa
Malta
Montenegro
Switzerland
Belgium
Egypt
Morocco
Canada USA
Australia&New Zealand
Iraq
Georgia
0
20
40
60
80

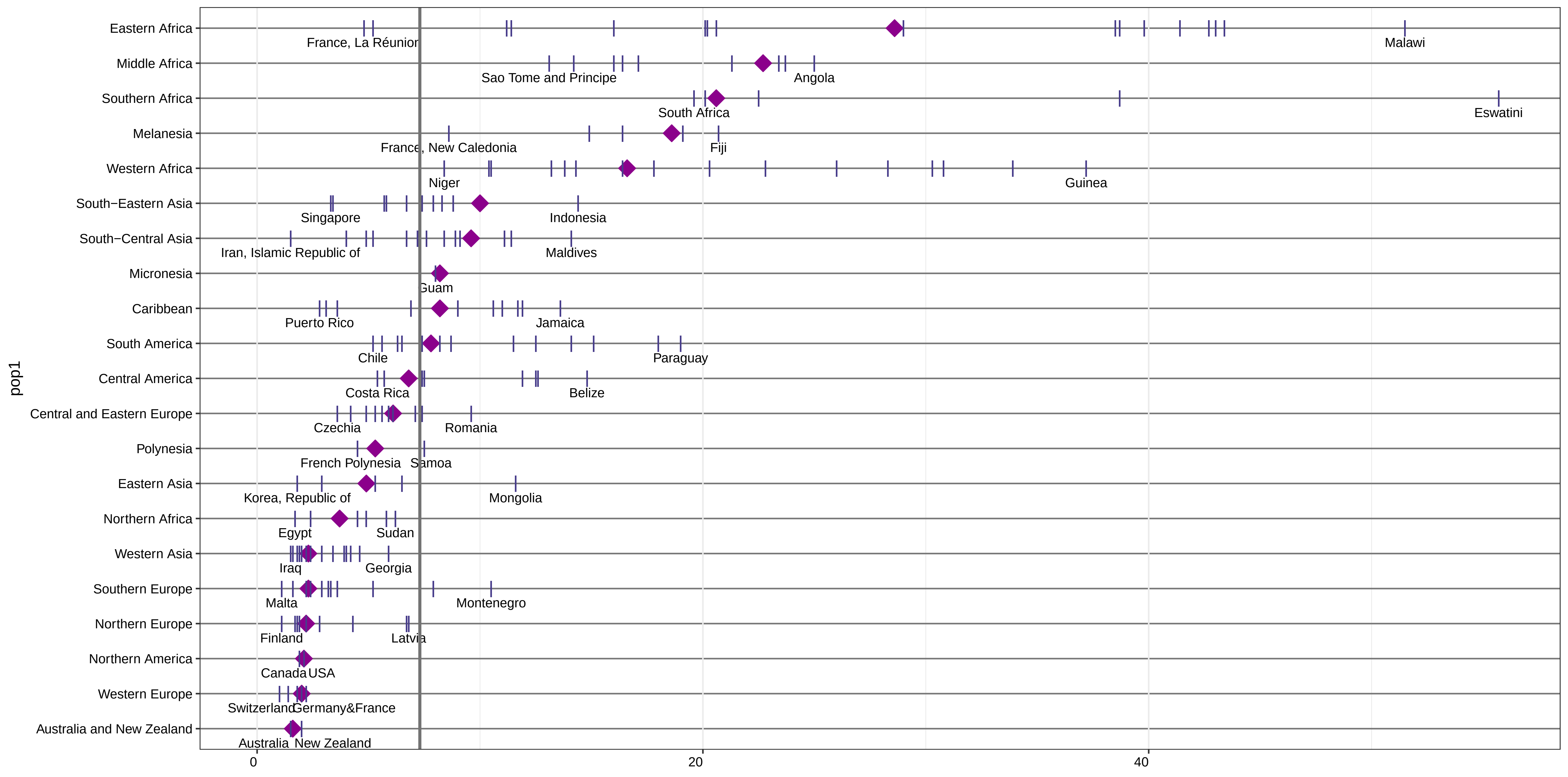

Eastern Africa
Middle Africa
Southern Africa
Melanesia
Western Africa
South−Eastern Asia
South−Central Asia
Micronesia
Caribbean
South America
Central America
Central and Eastern Europe
Polynesia
Eastern Asia
Northern Africa
Western Asia
Southern Europe
Northern Europe
Northern America
Western Europe
Australia and New Zealand
pop1
France, La Réunion
Malawi
Sao Tome and Principe
Angola
South Africa
Eswatini
France, New Caledonia
Fiji
Niger
Guinea
Singapore
Indonesia
Iran, Islamic Republic of
Maldives
Guam
Puerto Rico
Jamaica
Chile
Paraguay
Costa Rica
Belize
Czechia
Romania
French Polynesia
Samoa
Korea, Republic of
Mongolia
Egypt
Sudan
Iraq
Georgia
Malta
Montenegro
Finland
Latvia
Canada
USA
Switzerland
Germany&France
Australia
New Zealand
0
20
40

Estimated age-standardized incidence rates (World) in 2020, cervix uteri, females, all ages

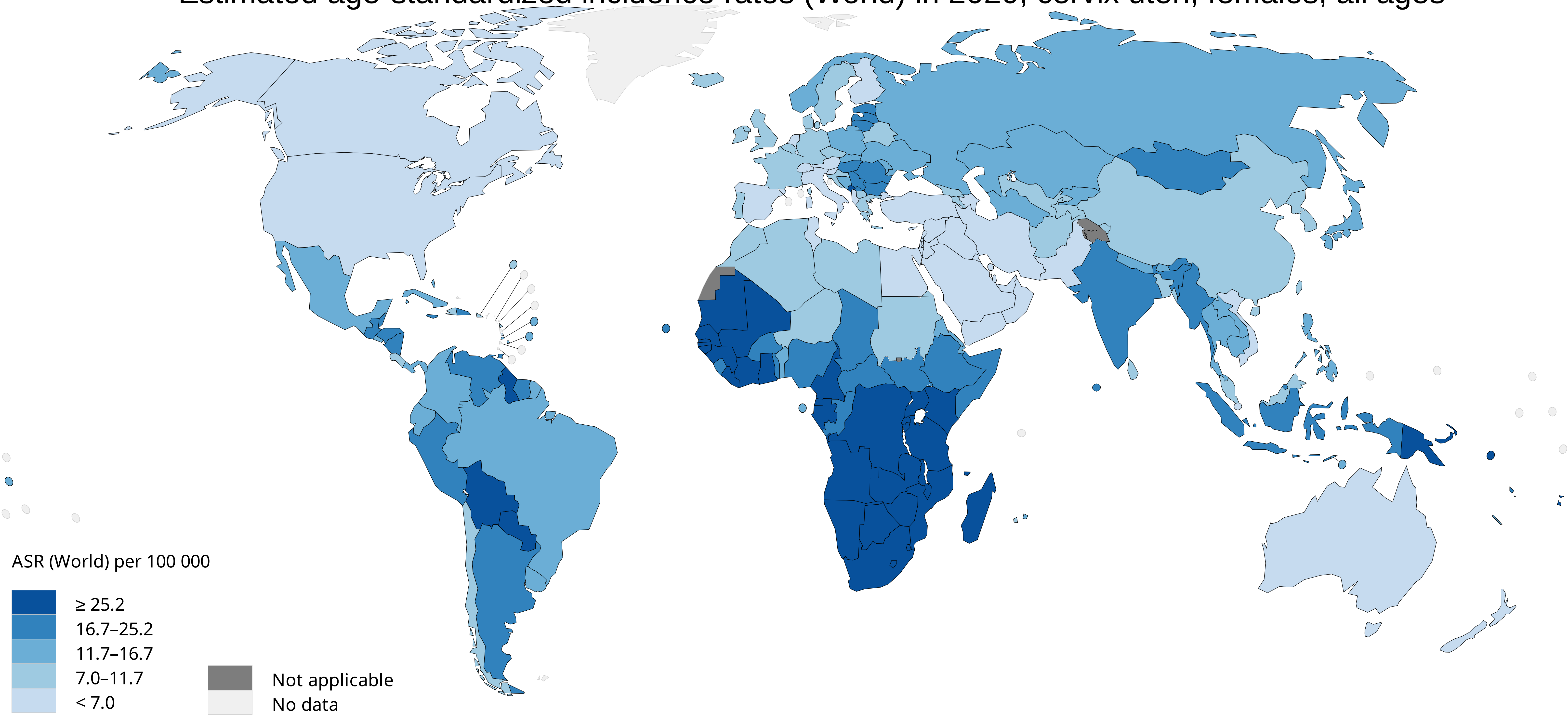

ASR (World) per 100 000

- ≥ 25.2
- 16.7–25.2
- 11.7–16.7
- 7.0–11.7
- < 7.0
- Not applicable
- No data

All rights reserved. The designations employed and the presentation of the material in this publication do not imply the expression of any opinion whatsoever on the part of the World Health Organization / International Agency for Research on Cancer concerning the legal status of any country, territory, city or area or of its authorities, or concerning the delimitation of its frontiers or boundaries. Dotted and dashed lines on maps represent approximate borderlines for which there may not yet be full agreement.

Data source: GLOBOCAN 2020
Map production: IARC
(http://gco.iarc.fr/today)
World Health Organization

World Health Organization

© International Agency for Research on Cancer 2020
All rights reserved

Estimated age-standardized mortality rates (World) in 2020, cervix uteri, females, all ages

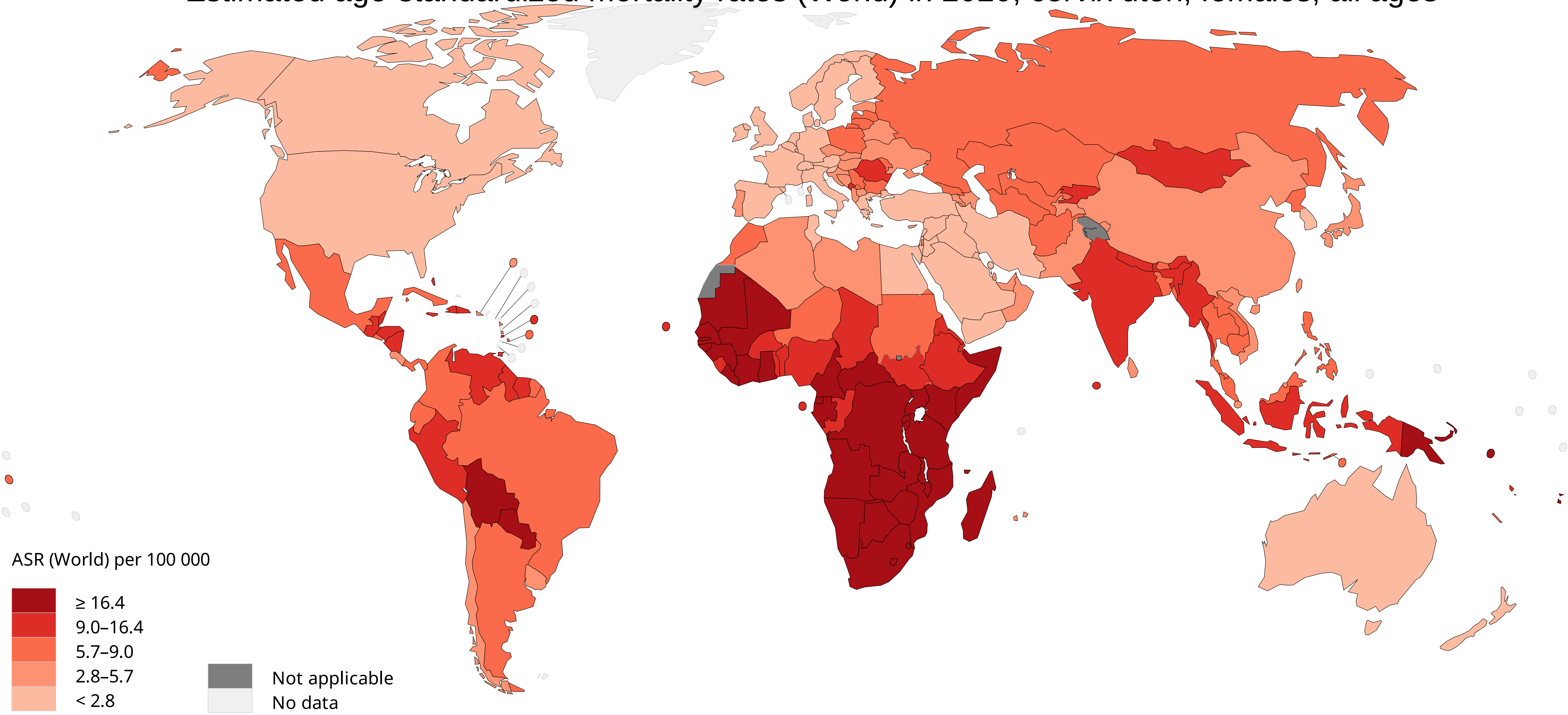

All rights reserved. The designations employed and the presentation of the material in this publication do not imply the expression of any opinion whatsoever on the part of the World Health Organization / International Agency for Research on Cancer concerning the legal status of any country, territory, city or area or of its authorities, or concerning the delimitation of its frontiers or boundaries. Dotted and dashed lines on maps represent approximate borderlines for which there may not yet be full agreement.

Data source: GLOBOCAN 2020
Map production: IARC
(http://gco.iarc.fr/today)
World Health Organization

World Health Organization

© International Agency for Research on Cancer 2020
All rights reserved

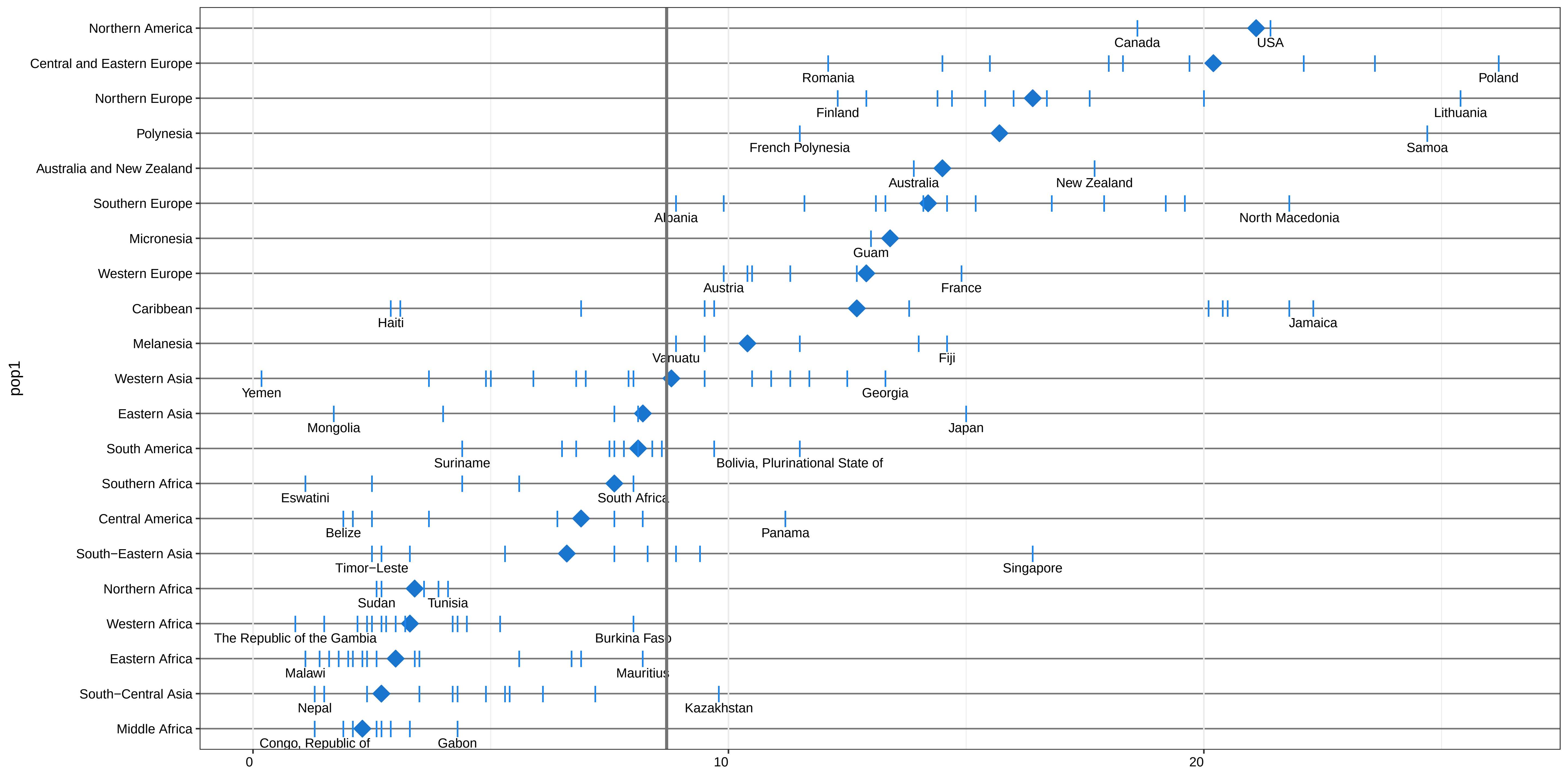

pop1
Northern America
Central and Eastern Europe
Northern Europe
Polynesia
Australia and New Zealand
Southern Europe
Micronesia
Western Europe
Caribbean
Melanesia
Western Asia
Eastern Asia
South America
Southern Africa
Central America
South−Eastern Asia
Northern Africa
Western Africa
Eastern Africa
South−Central Asia
Middle Africa
Canada
USA
Romania
Poland
Finland
Lithuania
French Polynesia
Samoa
Australia
New Zealand
Albania
North Macedonia
Guam
Austria
France
Haiti
Jamaica
Vanuatu
Fiji
Yemen
Georgia
Mongolia
Japan
Suriname
Bolivia, Plurinational State of
Eswatini
South Africa
Belize
Panama
Timor−Leste
Singapore
Sudan
Tunisia
The Republic of the Gambia
Burkina Faso
Malawi
Mauritius
Nepal
Kazakhstan
Congo, Republic of
Gabon
0
10
20

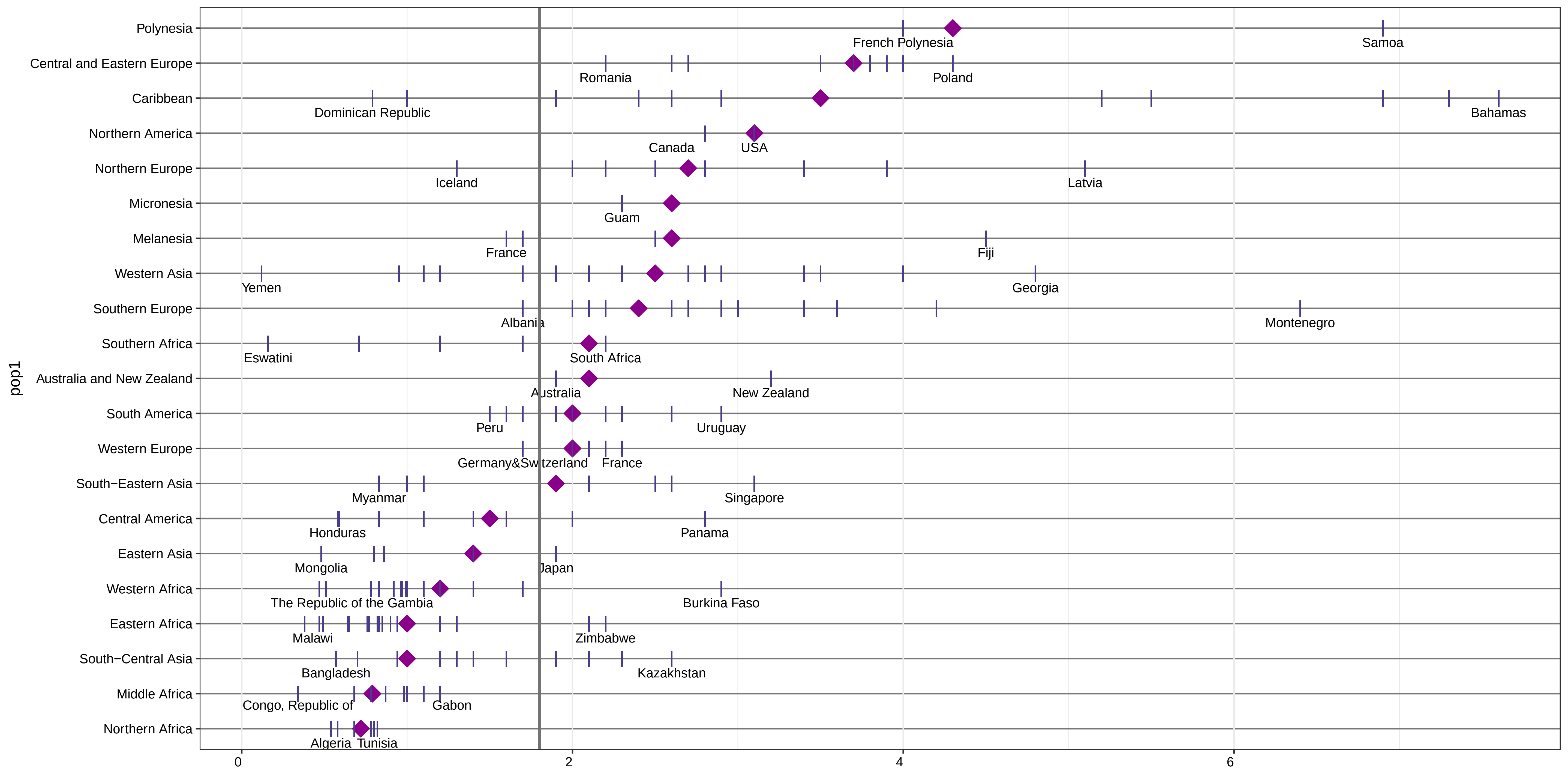

pop1
Polynesia
Central and Eastern Europe
Caribbean
Northern America
Northern Europe
Micronesia
Melanesia
Western Asia
Southern Europe
Southern Africa
Australia and New Zealand
South America
Western Europe
South−Eastern Asia
Central America
Eastern Asia
Western Africa
Eastern Africa
South−Central Asia
Middle Africa
Northern Africa
French Polynesia
Samoa
Romania
Poland
Dominican Republic
Bahamas
Canada
USA
Iceland
Latvia
Guam
France
Fiji
Yemen
Georgia
Albania
Montenegro
Eswatini
South Africa
Australia
New Zealand
Peru
Uruguay
Germany&Switzerland
France
Myanmar
Singapore
Honduras
Panama
Mongolia
Japan
The Republic of the Gambia
Burkina Faso
Malawi
Zimbabwe
Bangladesh
Kazakhstan
Congo, Republic of
Gabon
Algeria
Tunisia
0
2
4
6

Estimated age-standardized incidence rates (World) in 2020, corpus uteri, females, all ages

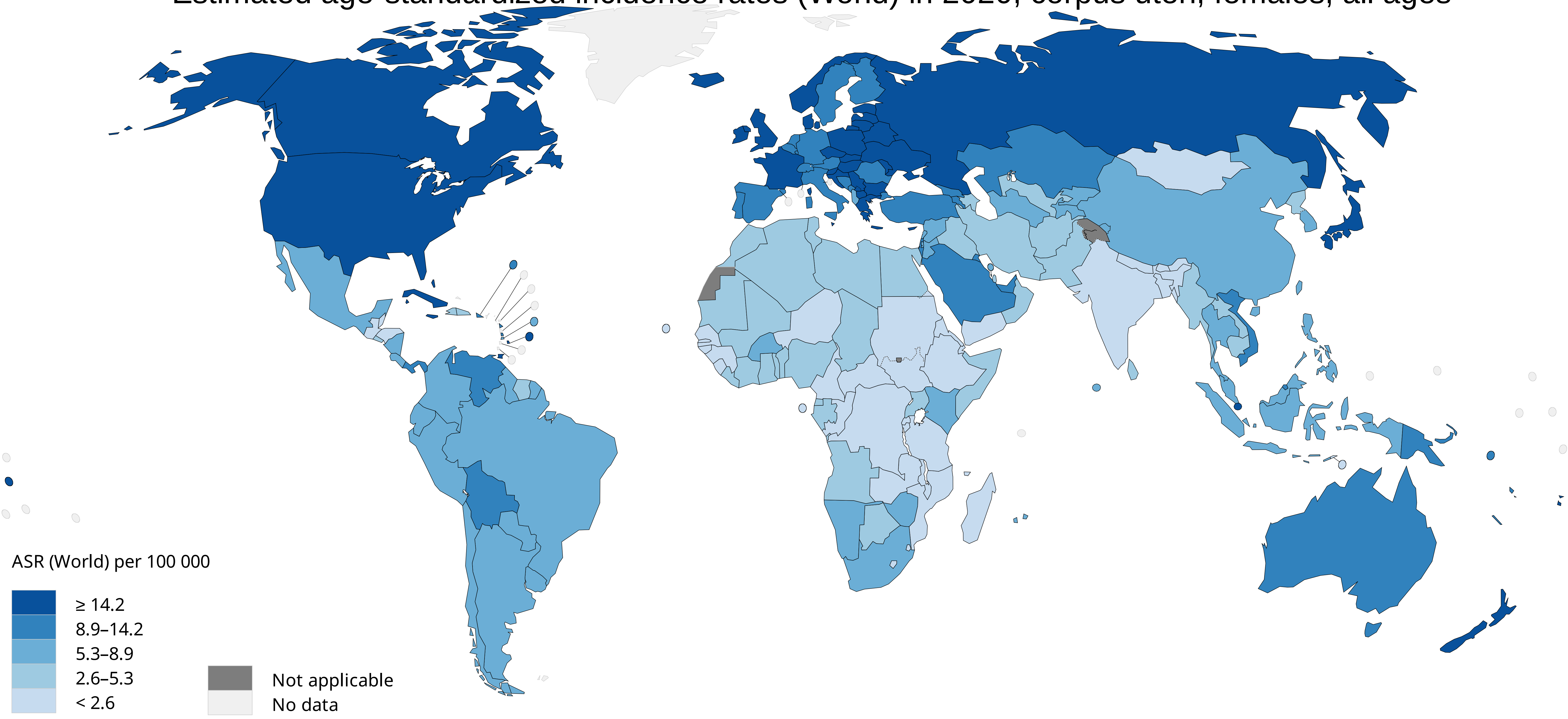

All rights reserved. The designations employed and the presentation of the material in this publication do not imply the expression of any opinion whatsoever on the part of the World Health Organization / International Agency for Research on Cancer concerning the legal status of any country, territory, city or area or of its authorities, or concerning the delimitation of its frontiers or boundaries. Dotted and dashed lines on maps represent approximate borderlines for which there may not yet be full agreement.

Data source: GLOBOCAN 2020
Map production: IARC
(http://gco.iarc.fr/today)
World Health Organization

World Health Organization

© International Agency for Research on Cancer 2020
All rights reserved

Estimated age-standardized mortality rates (World) in 2020, corpus uteri, females, all ages

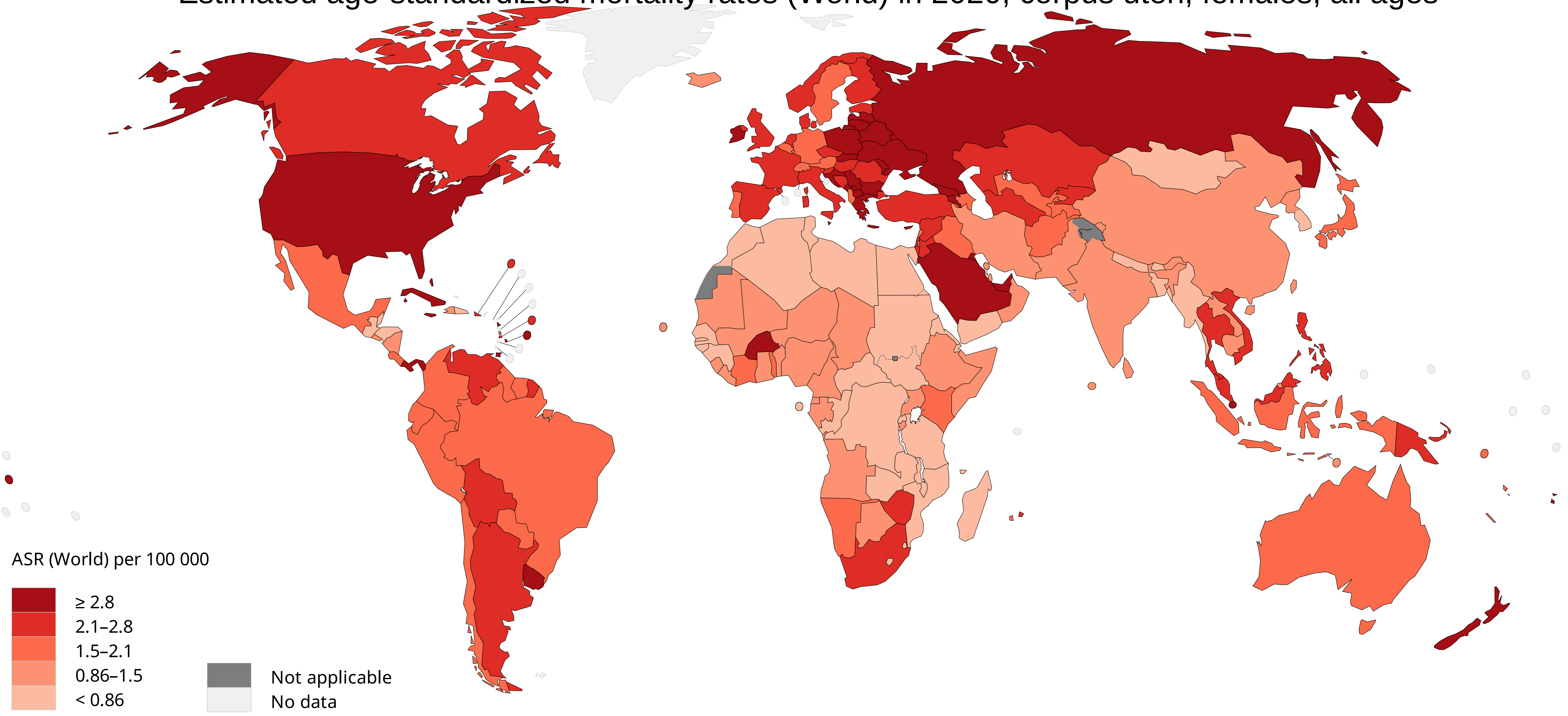

All rights reserved. The designations employed and the presentation of the material in this publication do not imply the expression of any opinion whatsoever on the part of the World Health Organization / International Agency for Research on Cancer concerning the legal status of any country, territory, city or area or of its authorities, or concerning the delimitation of its frontiers or boundaries. Dotted and dashed lines on maps represent approximate borderlines for which there may not yet be full agreement.

Data source: GLOBOCAN 2020
Map production: IARC
(http://gco.iarc.fr/today)
World Health Organization

© International Agency for Research on Cancer 2020
All rights reserved

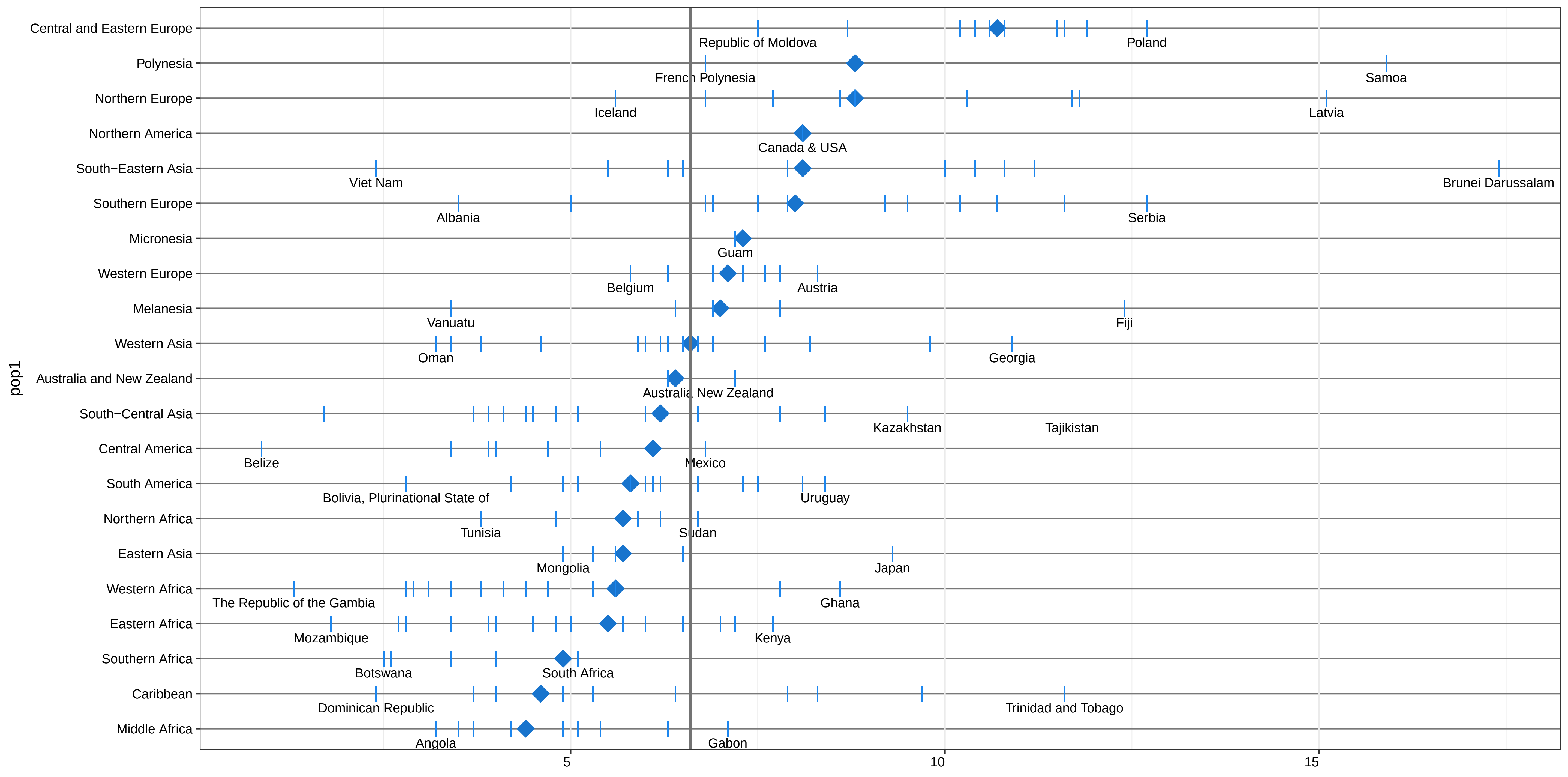
Central and Eastern Europe
Republic of Moldova
Poland
Polynesia
French Polynesia
Samoa
Northern Europe
Iceland
Latvia
Northern America
Canada & USA
South−Eastern Asia
Viet Nam
Brunei Darussalam
Southern Europe
Albania
Serbia
Micronesia
Guam
Western Europe
Belgium
Austria
Melanesia
Vanuatu
Fiji
Western Asia
Oman
Georgia
Australia and New Zealand
Australia
New Zealand
South−Central Asia
Kazakhstan
Tajikistan
Central America
Belize
Mexico
South America
Bolivia, Plurinational State of
Uruguay
Northern Africa
Tunisia
Sudan
Eastern Asia
Mongolia
Japan
Western Africa
The Republic of the Gambia
Ghana
Eastern Africa
Mozambique
Kenya
Southern Africa
Botswana
South Africa
Caribbean
Dominican Republic
Trinidad and Tobago
Middle Africa
Angola
Gabon
pop1
5
10
15

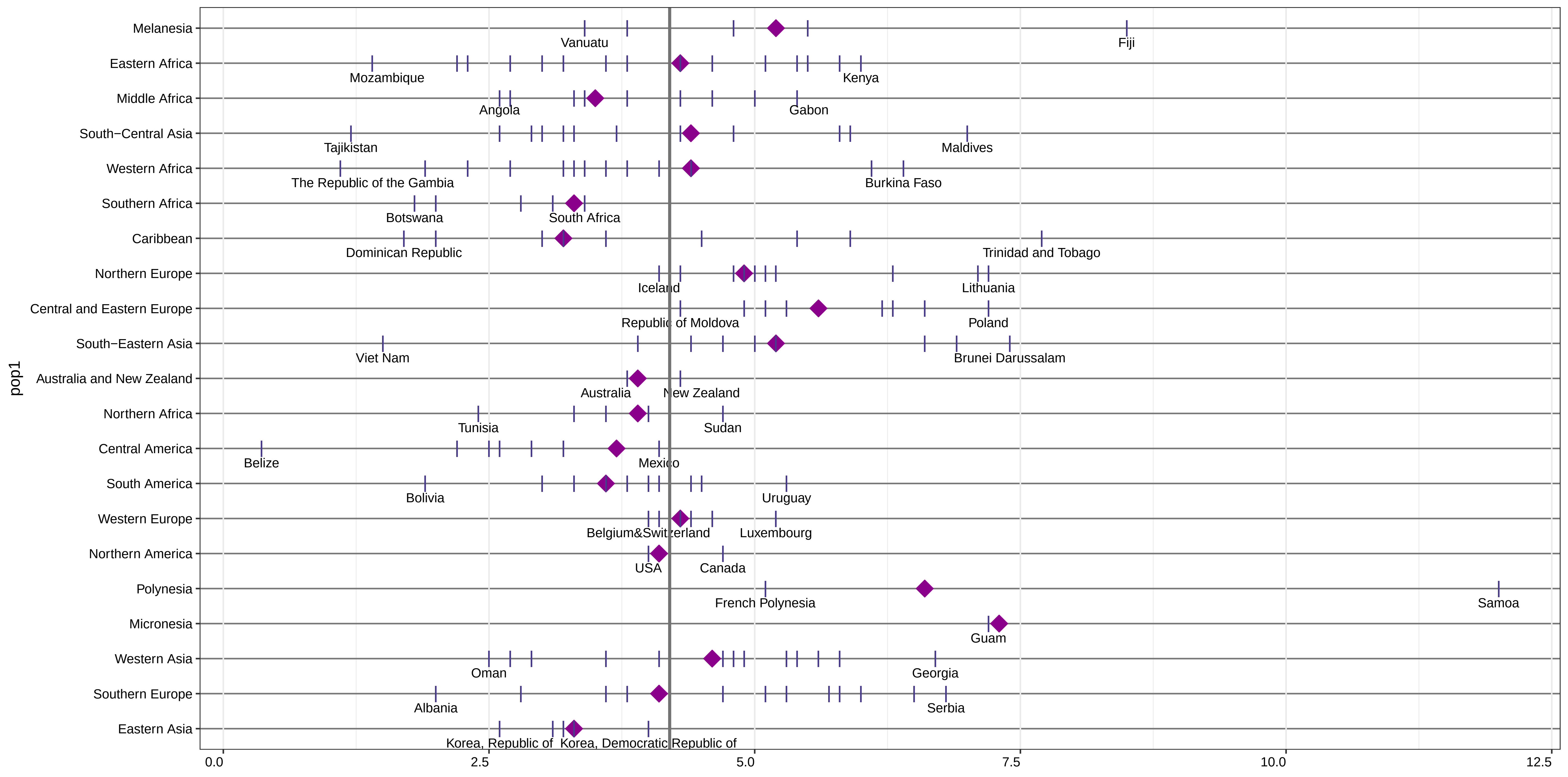
pop1
Melanesia
Eastern Africa
Middle Africa
South−Central Asia
Western Africa
Southern Africa
Caribbean
Northern Europe
Central and Eastern Europe
South−Eastern Asia
Australia and New Zealand
Northern Africa
Central America
South America
Western Europe
Northern America
Polynesia
Micronesia
Western Asia
Southern Europe
Eastern Asia
Vanuatu
Fiji
Mozambique
Kenya
Angola
Gabon
Tajikistan
Maldives
The Republic of the Gambia
Burkina Faso
Botswana
South Africa
Dominican Republic
Trinidad and Tobago
Iceland
Lithuania
Republic of Moldova
Poland
Viet Nam
Brunei Darussalam
Australia
New Zealand
Tunisia
Sudan
Belize
Mexico
Bolivia
Uruguay
Belgium&Switzerland
Luxembourg
USA
Canada
French Polynesia
Samoa
Guam
Oman
Georgia
Albania
Serbia
Korea, Republic of
Korea, Democratic Republic of
0.0
2.5
5.0
7.5
10.0
12.5

Estimated age-standardized incidence rates (World) in 2020, ovary, females, all ages

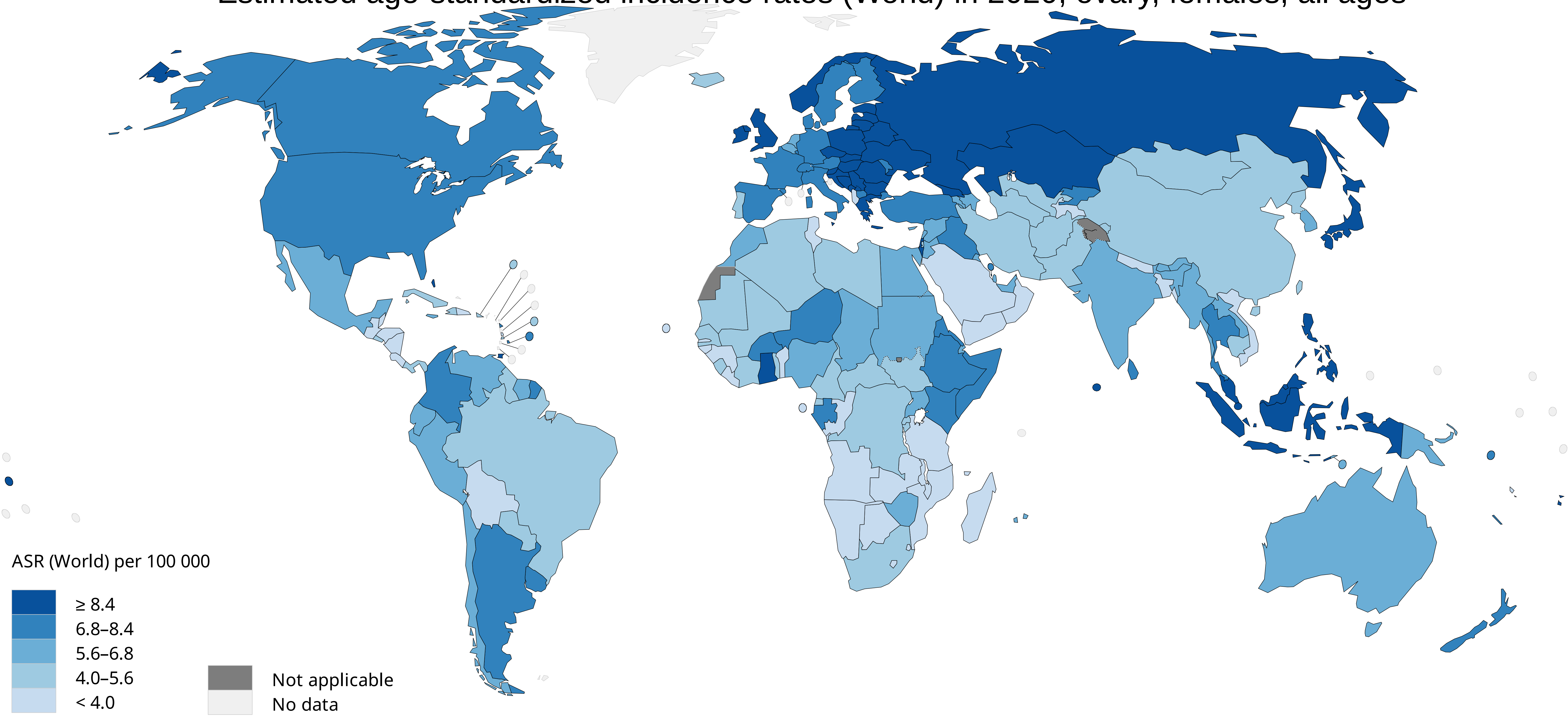

All rights reserved. The designations employed and the presentation of the material in this publication do not imply the expression of any opinion whatsoever on the part of the World Health Organization / International Agency for Research on Cancer concerning the legal status of any country, territory, city or area or of its authorities, or concerning the delimitation of its frontiers or boundaries. Dotted and dashed lines on maps represent approximate borderlines for which there may not yet be full agreement.

Data source: GLOBOCAN 2020
Map production: IARC
(http://gco.iarc.fr/today)
World Health Organization

World Health Organization

© International Agency for Research on Cancer 2020
All rights reserved

Estimated age-standardized mortality rates (World) in 2020, ovary, females, all ages

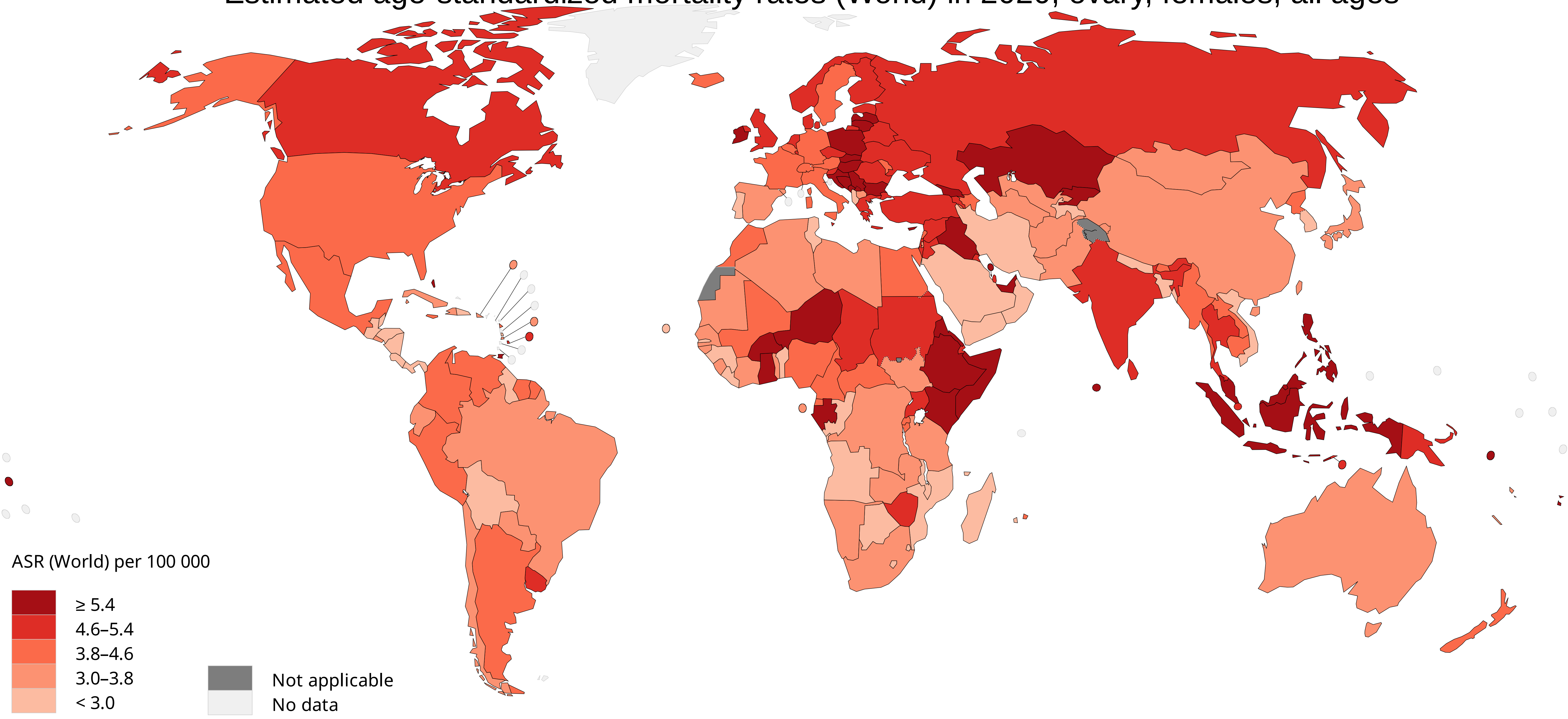

All rights reserved. The designations employed and the presentation of the material in this publication do not imply the expression of any opinion whatsoever on the part of the World Health Organization / International Agency for Research on Cancer concerning the legal status of any country, territory, city or area or of its authorities, or concerning the delimitation of its frontiers or boundaries. Dotted and dashed lines on maps represent approximate borderlines for which there may not yet be full agreement.

Data source: GLOBOCAN 2020
Map production: IARC
(http://gco.iarc.fr/today)
World Health Organization

World Health Organization

© International Agency for Research on Cancer 2020
All rights reserved

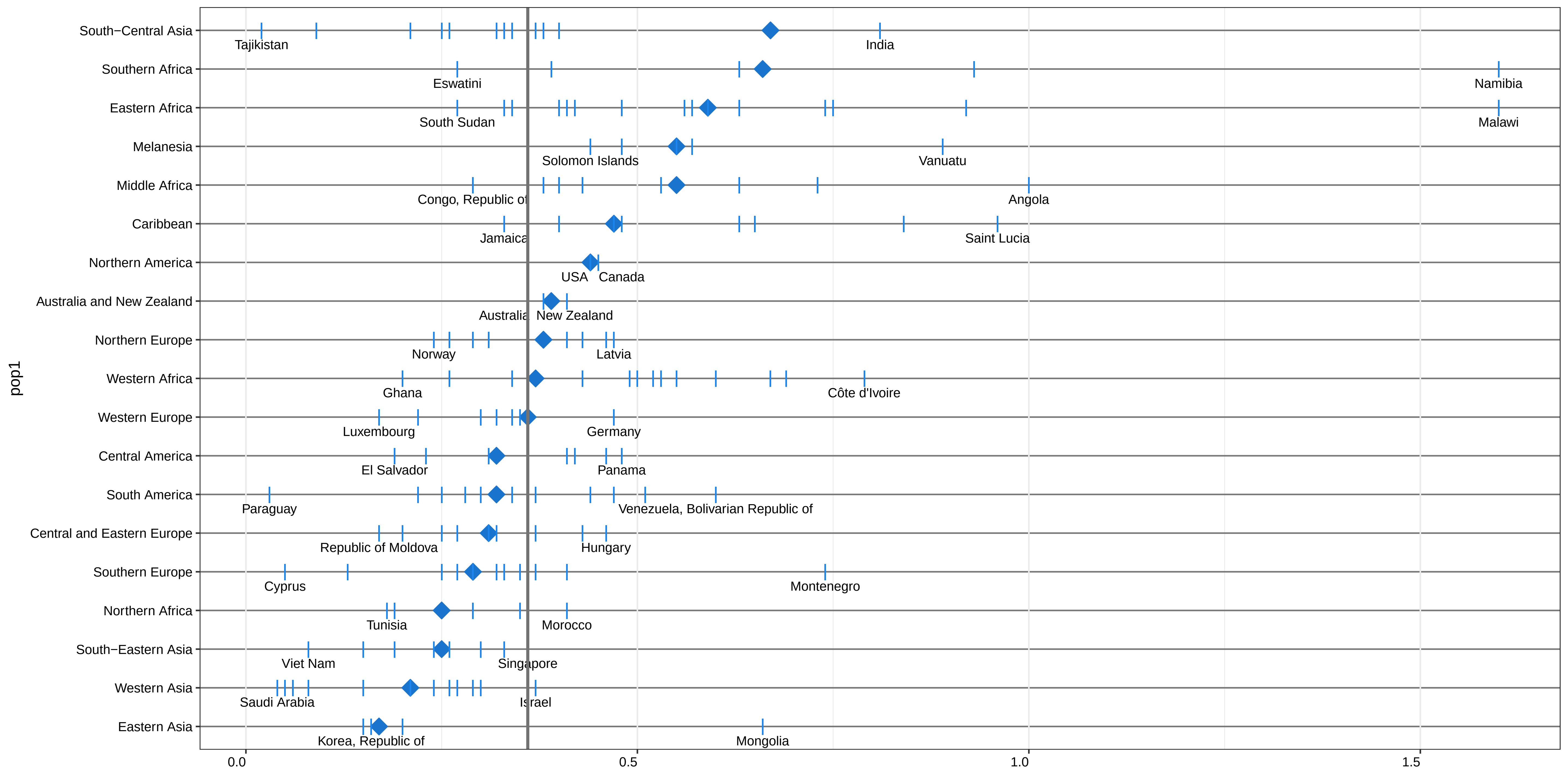
South−Central Asia
Southern Africa
Eastern Africa
Melanesia
Middle Africa
Caribbean
Northern America
Australia and New Zealand
Northern Europe
Western Africa
Western Europe
Central America
South America
Central and Eastern Europe
Southern Europe
Northern Africa
South−Eastern Asia
Western Asia
Eastern Asia
pop1
Tajikistan
India
Eswatini
Namibia
South Sudan
Malawi
Solomon Islands
Vanuatu
Congo, Republic of
Angola
Jamaica
Saint Lucia
USA
Canada
Australia
New Zealand
Norway
Latvia
Ghana
Côte d'Ivoire
Luxembourg
Germany
El Salvador
Panama
Paraguay
Venezuela, Bolivarian Republic of
Republic of Moldova
Hungary
Cyprus
Montenegro
Tunisia
Morocco
Viet Nam
Singapore
Saudi Arabia
Israel
Korea, Republic of
Mongolia
0.0
0.5
1.0
1.5

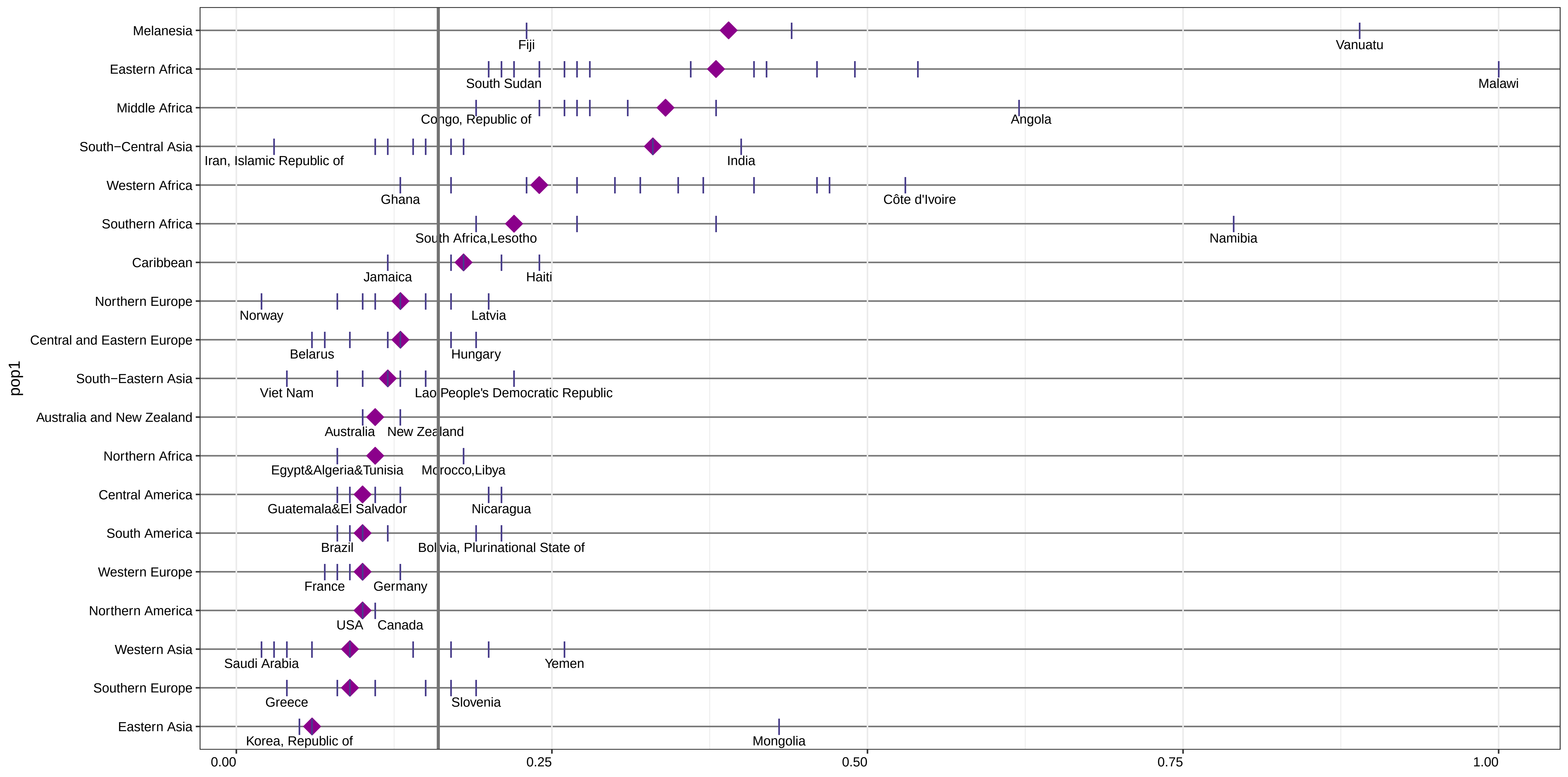

Melanesia
Eastern Africa
Middle Africa
South−Central Asia
Western Africa
Southern Africa
Caribbean
Northern Europe
Central and Eastern Europe
South−Eastern Asia
Australia and New Zealand
Northern Africa
Central America
South America
Western Europe
Northern America
Western Asia
Southern Europe
Eastern Asia
pop1
Fiji
Vanuatu
South Sudan
Malawi
Congo, Republic of
Angola
Iran, Islamic Republic of
India
Ghana
Côte d'Ivoire
South Africa,Lesotho
Namibia
Jamaica
Haiti
Norway
Latvia
Belarus
Hungary
Viet Nam
Lao People's Democratic Republic
Australia
New Zealand
Egypt&Algeria&Tunisia
Morocco,Libya
Guatemala&El Salvador
Nicaragua
Brazil
Bolivia, Plurinational State of
France
Germany
USA
Canada
Saudi Arabia
Yemen
Greece
Slovenia
Korea, Republic of
Mongolia
0.00
0.25
0.50
0.75
1.00

Estimated age-standardized incidence rates (World) in 2020, vagina, females, all ages

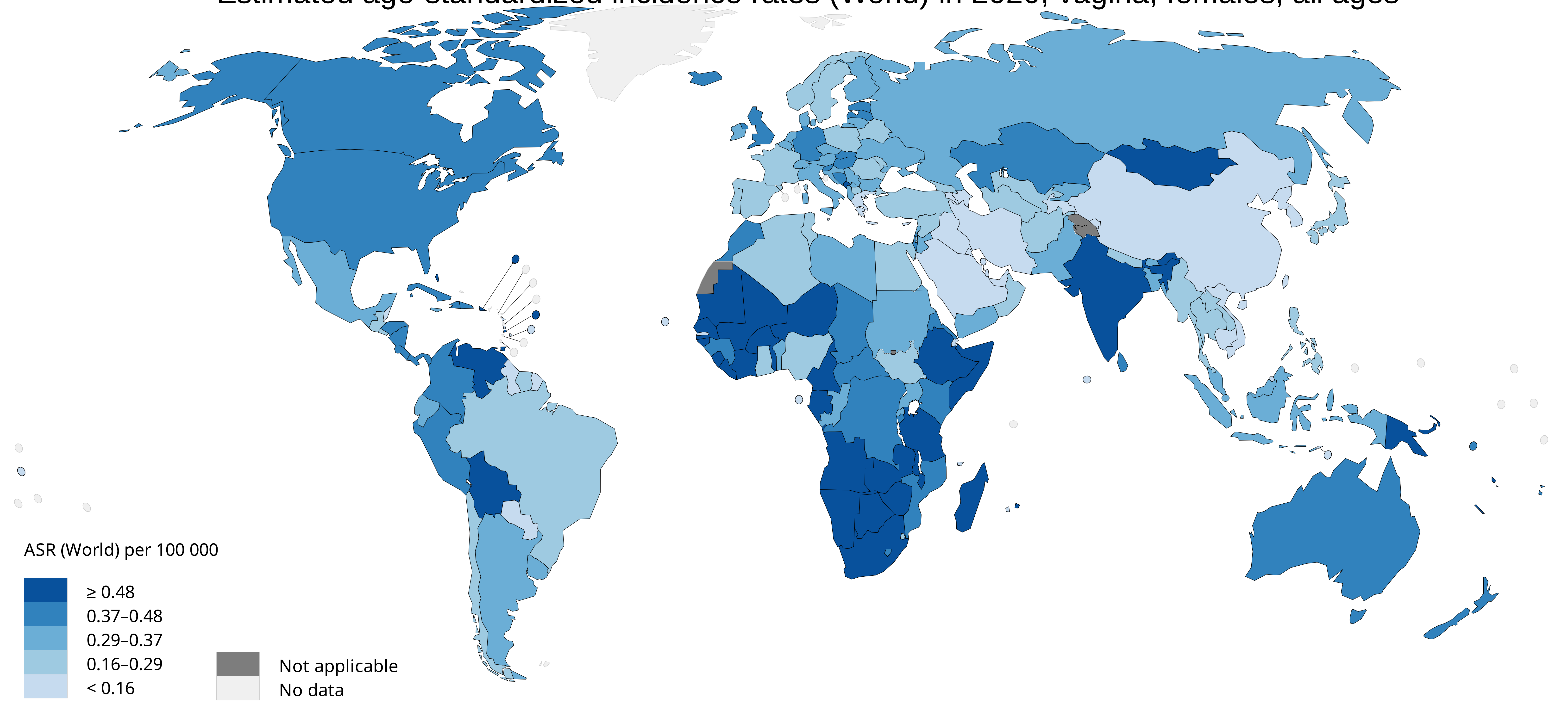

All rights reserved. The designations employed and the presentation of the material in this publication do not imply the expression of any opinion whatsoever on the part of the World Health Organization / International Agency for Research on Cancer concerning the legal status of any country, territory, city or area or of its authorities, or concerning the delimitation of its frontiers or boundaries. Dotted and dashed lines on maps represent approximate borderlines for which there may not yet be full agreement.

Data source: GLOBOCAN 2020
Map production: IARC
(http://gco.iarc.fr/today)
World Health Organization

World Health Organization

© International Agency for Research on Cancer 2020
All rights reserved

Estimated age-standardized mortality rates (World) in 2020, vagina, females, all ages

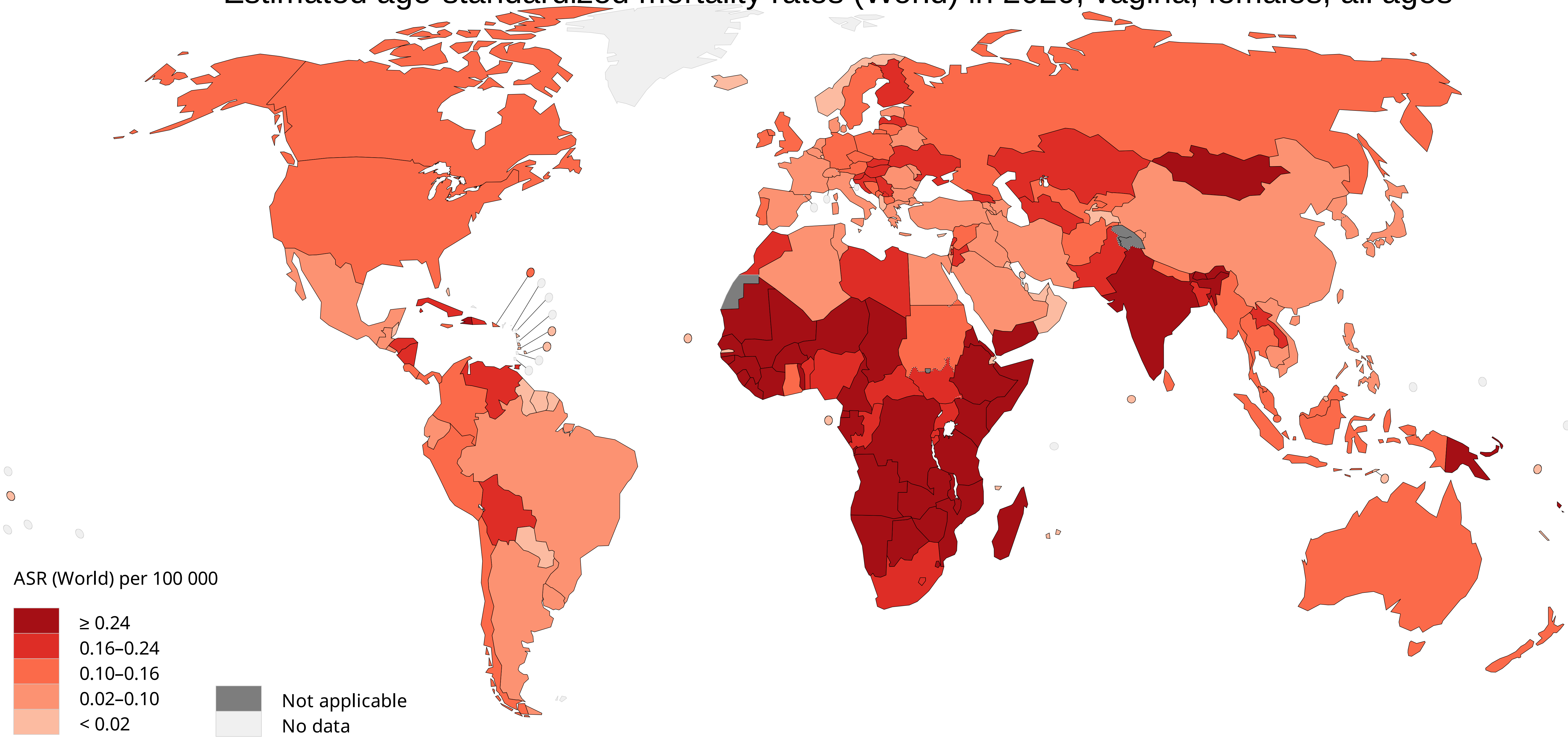

All rights reserved. The designations employed and the presentation of the material in this publication do not imply the expression of any opinion whatsoever on the part of the World Health Organization / International Agency for Research on Cancer concerning the legal status of any country, territory, city or area or of its authorities, or concerning the delimitation of its frontiers or boundaries. Dotted and dashed lines on maps represent approximate borderlines for which there may not yet be full agreement.

Data source: GLOBOCAN 2020
Map production: IARC
(http://gco.iarc.fr/today)
World Health Organization

© International Agency for Research on Cancer 2020
All rights reserved

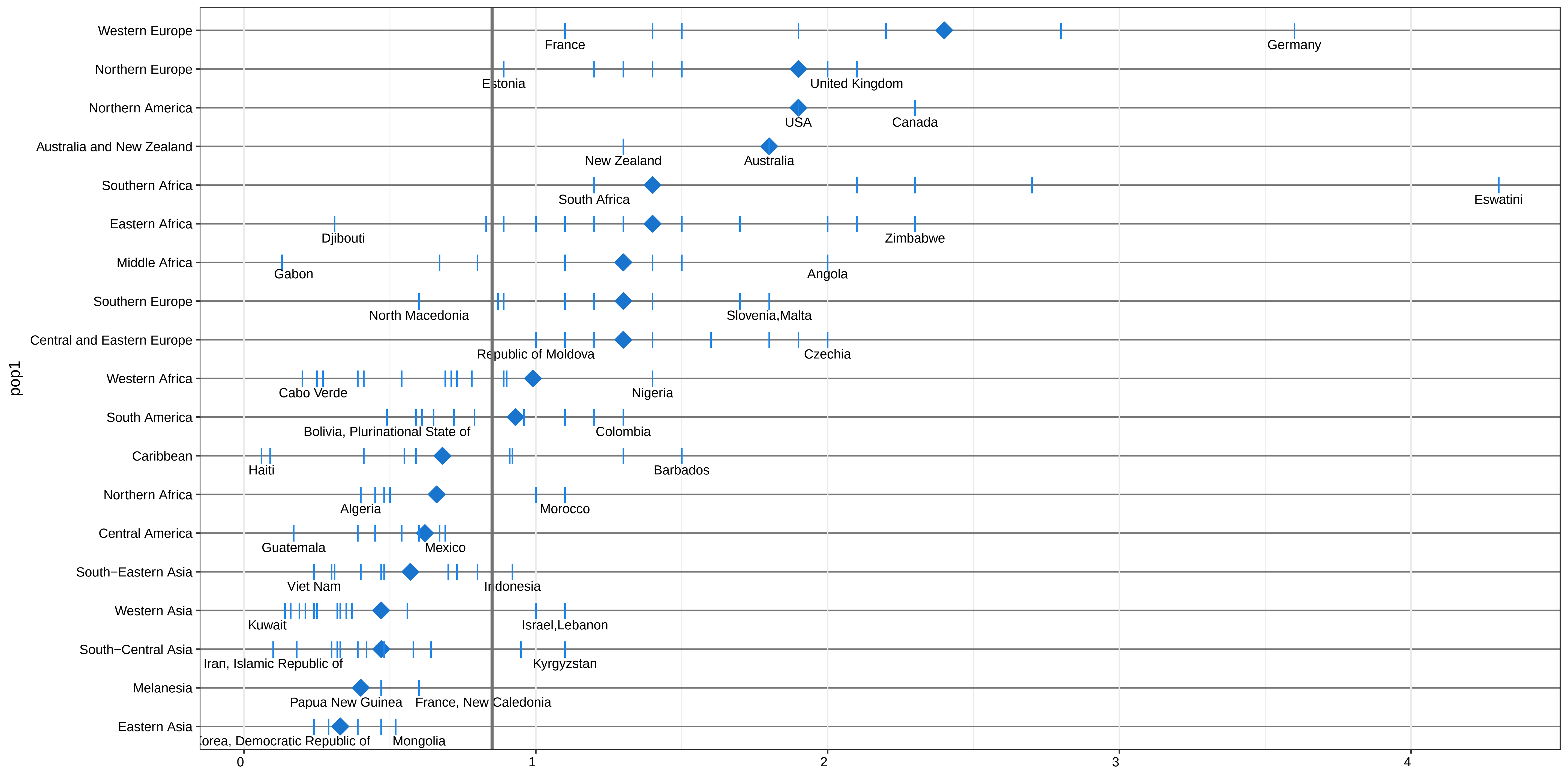

pop1
Western Europe
Northern Europe
Northern America
Australia and New Zealand
Southern Africa
Eastern Africa
Middle Africa
Southern Europe
Central and Eastern Europe
Western Africa
South America
Caribbean
Northern Africa
Central America
South−Eastern Asia
Western Asia
South−Central Asia
Melanesia
Eastern Asia
France
Germany
Estonia
United Kingdom
USA
Canada
New Zealand
Australia
South Africa
Eswatini
Djibouti
Zimbabwe
Gabon
Angola
North Macedonia
Slovenia,Malta
Republic of Moldova
Czechia
Cabo Verde
Nigeria
Bolivia, Plurinational State of
Colombia
Haiti
Barbados
Algeria
Morocco
Guatemala
Mexico
Viet Nam
Indonesia
Kuwait
Israel,Lebanon
Iran, Islamic Republic of
Kyrgyzstan
Papua New Guinea
France, New Caledonia
Korea, Democratic Republic of
Mongolia
0
1
2
3
4

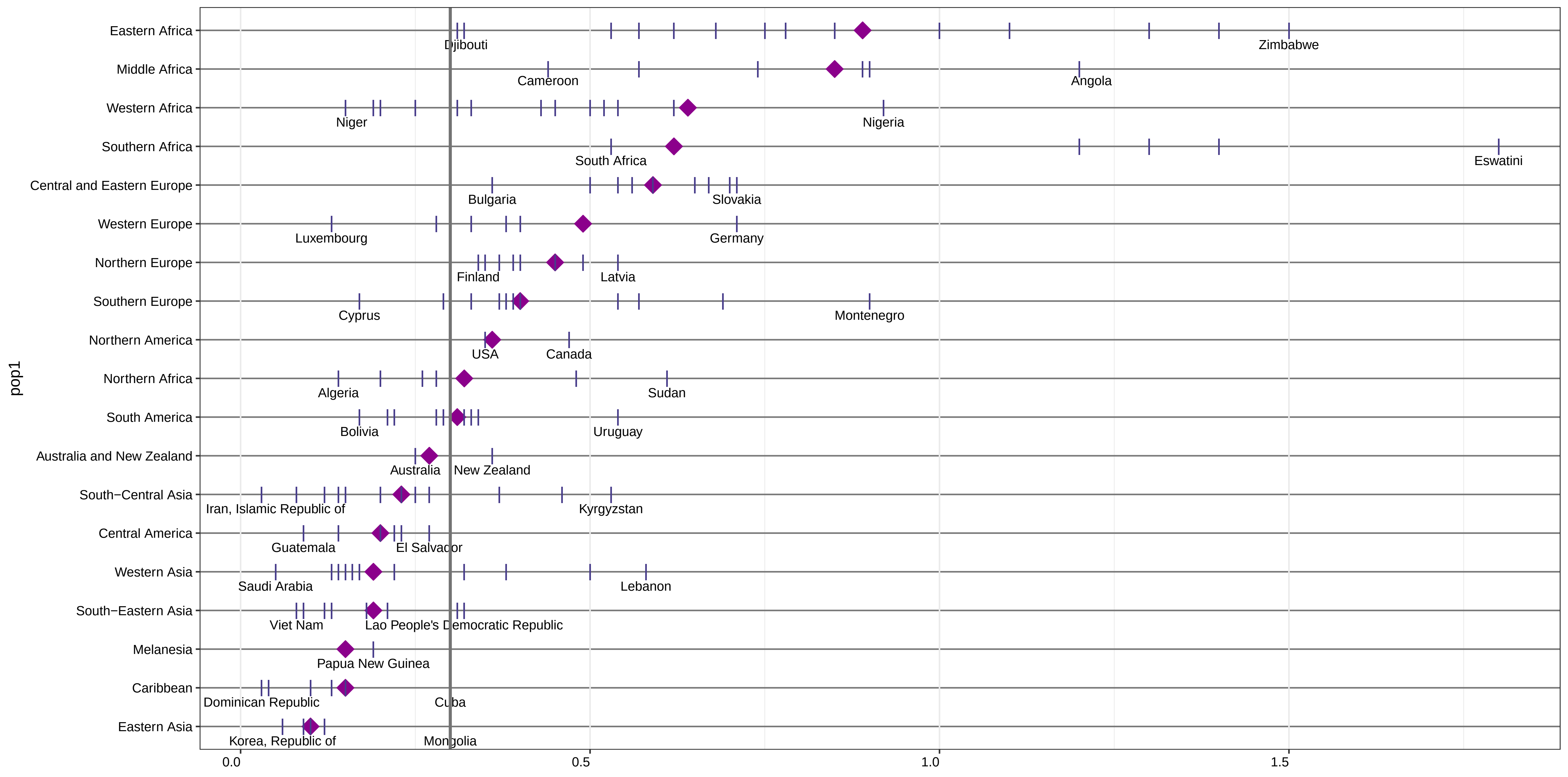

pop1
Eastern Africa
Middle Africa
Western Africa
Southern Africa
Central and Eastern Europe
Western Europe
Northern Europe
Southern Europe
Northern America
Northern Africa
South America
Australia and New Zealand
South−Central Asia
Central America
Western Asia
South−Eastern Asia
Melanesia
Caribbean
Eastern Asia
Djibouti
Zimbabwe
Cameroon
Angola
Niger
Nigeria
South Africa
Eswatini
Bulgaria
Slovakia
Luxembourg
Germany
Finland
Latvia
Cyprus
Montenegro
USA
Canada
Algeria
Sudan
Bolivia
Uruguay
Australia
New Zealand
Iran, Islamic Republic of
Kyrgyzstan
Guatemala
El Salvador
Saudi Arabia
Lebanon
Viet Nam
Lao People's Democratic Republic
Papua New Guinea
Dominican Republic
Cuba
Korea, Republic of
Mongolia
0.0
0.5
1.0
1.5

Estimated age-standardized incidence rates (World) in 2020, vulva, females, all ages

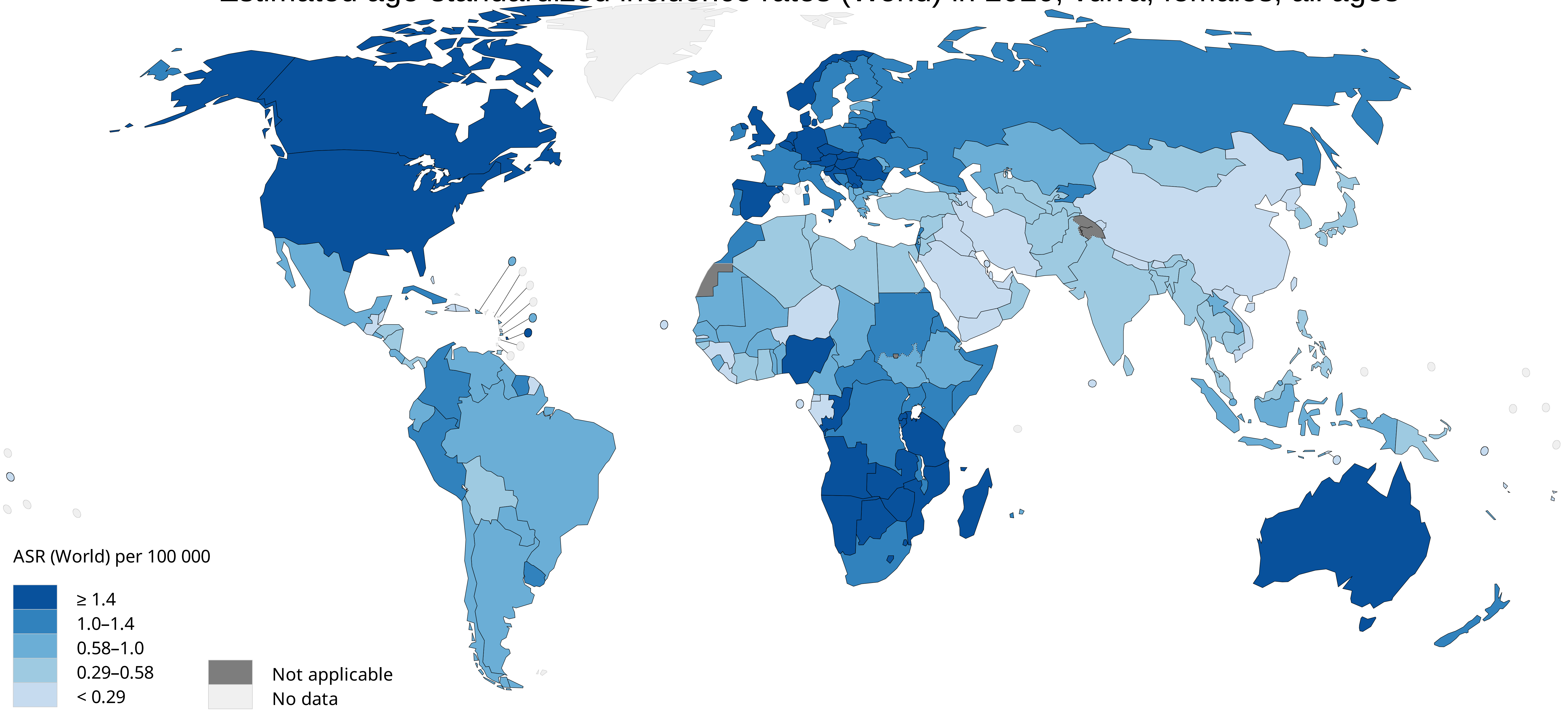

All rights reserved. The designations employed and the presentation of the material in this publication do not imply the expression of any opinion whatsoever on the part of the World Health Organization / International Agency for Research on Cancer concerning the legal status of any country, territory, city or area or of its authorities, or concerning the delimitation of its frontiers or boundaries. Dotted and dashed lines on maps represent approximate borderlines for which there may not yet be full agreement.

Data source: GLOBOCAN 2020
Map production: IARC
(http://gco.iarc.fr/today)
World Health Organization

World Health Organization

© International Agency for Research on Cancer 2020
All rights reserved

Estimated age-standardized mortality rates (World) in 2020, vulva, females, all ages

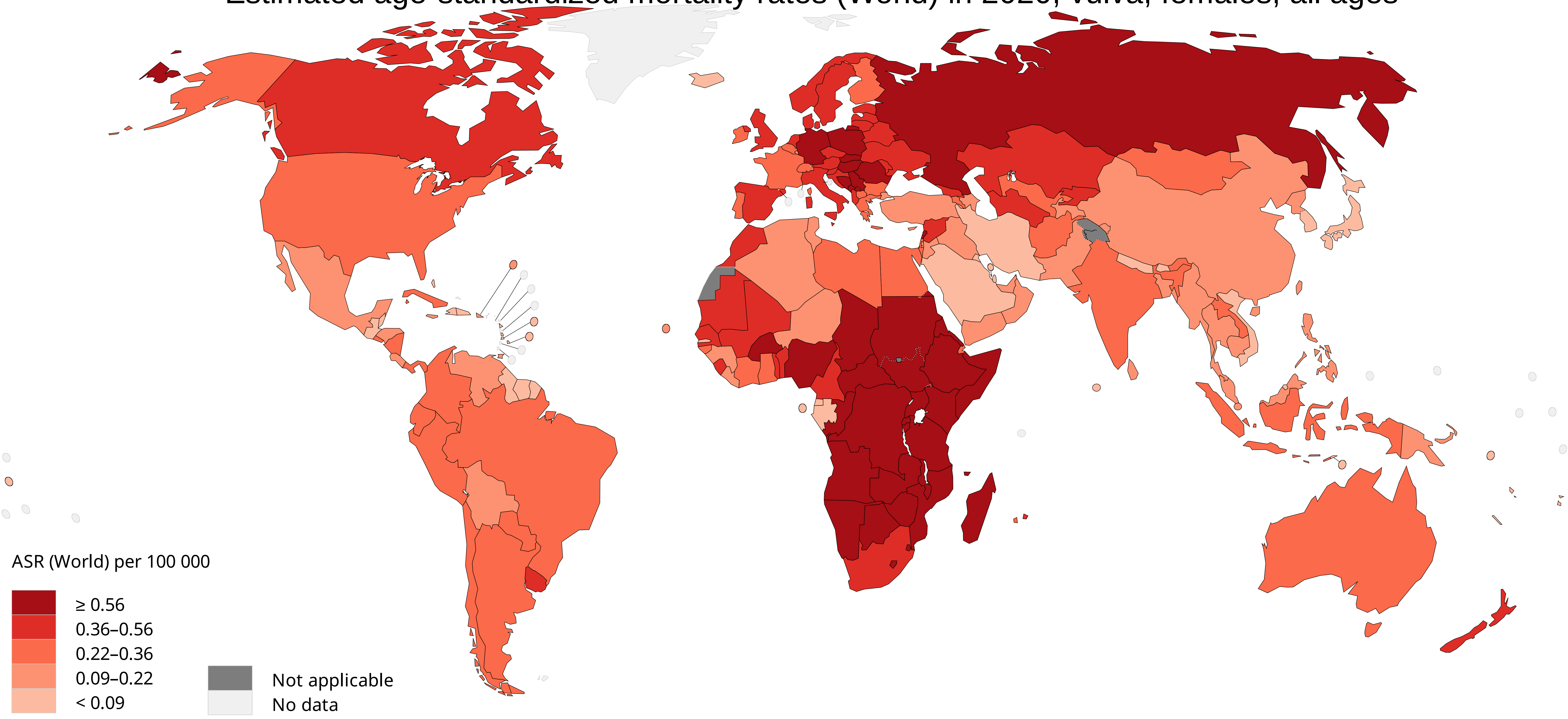

All rights reserved. The designations employed and the presentation of the material in this publication do not imply the expression of any opinion whatsoever on the part of the World Health Organization / International Agency for Research on Cancer concerning the legal status of any country, territory, city or area or of its authorities, or concerning the delimitation of its frontiers or boundaries. Dotted and dashed lines on maps represent approximate borderlines for which there may not yet be full agreement.

Data source: GLOBOCAN 2020
Map production: IARC
(http://gco.iarc.fr/today)
World Health Organization

World Health Organization

© International Agency for Research on Cancer 2020
All rights reserved
